# Access to thioethers from thiols and alcohols via homogeneous and heterogeneous catalysis

**DOI:** 10.1038/s41598-023-47938-4

**Published:** 2023-11-23

**Authors:** Martyna Markwitz, Klaudiusz Labrzycki, Laura Azcune, Aitor Landa, Krzysztof Kuciński

**Affiliations:** 1 Faculty of Chemistry, Adam Mickiewicz University, Uniwersytetu Poznanskiego St. 8, 61-614 Poznan, Poland; 2 Department of Organic Chemistry I, Faculty of Chemistry, University of the Basque Country UPV/EHU, Paseo Manuel Lardizábal 3, 20018 San Sebastián, Spain

**Keywords:** Synthetic chemistry methodology, Catalysis

## Abstract

A metal-free dehydrative thioetherification method has been reported, enabling the conversion of various alcohols and thiols into thioethers. By employing triflic acid as a catalyst or utilizing a recyclable NAFION® superacid catalyst, these methods significantly improve the efficiency and practicality of sulfide preparation.

## Introduction

Organosulfur compounds have significant industrial importance, ranging from polymer production to their use as agrochemicals and pharmacologically active compounds^1–3^. Among the S-containing derivatives with such broad applications, thioethers, which contain a C–S–C bonds in their structure, are certainly included. At first glance, their resemblance to ethers is highly deceptive. This is because they exhibit completely different chemical and biological properties, primarily due to the divalent sulfur center's greater polarizability, surpassing that of oxygen in ethers^1^. This, in turn, drives chemists to constantly seek new methods for synthesizing this group of chemical compounds^4–15^.

**Figure 1:**
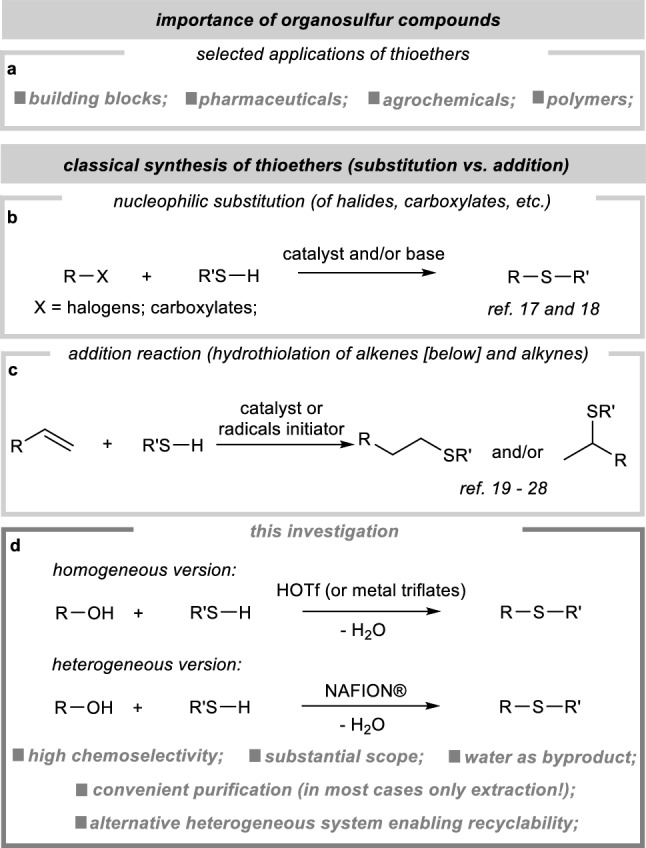
Context of the investigation.

Thioethers can be readily obtained by several methods (Fig. 1), but the main ones are those based on nucleophilic substitution or addition reactions (hydrothiolation^16^). The classical synthesis relies on the utilization of easily removable groups like halides^17^ or carboxylates^18^. However, these methods have a significant drawback as they produce substantial amounts of salt waste and pose challenges concerning process efficiency and chemoselectivity. On the other hand, we come across the aforementioned hydrothiolation. Here, it becomes paramount to ensure strict control over the process's selectivity, as it can result in the formation of various regioisomers^16,19,20^. So far, many methods have been developed primarily based on the use of free radicals^21^, transition metals^22–24^, and Lewis acids^25–28^. Taking into consideration the pros and cons discussed earlier and giving due importance to green chemistry aspects (e.g., the generation of non-toxic byproducts, the use of cost-effective and commercially available catalysts, the substitution of noble metal complexes, etc*.*), an ideal approach for synthesizing this compound involves a nucleophilic addition to alcohols, wherein the sole byproduct generated is water. In the existing literature, the procedures commonly describe the use of catalysts primarily composed of transition metal compounds^29–33^, particularly employing sub-stoichiometric amounts of their triflates^34–37^. From a synthetic perspective, the presence of residual metal impurities can create additional obstacles for industrial and pharmaceutical applications. Consequently, there is a strong need and desire to develop gentle and selective methods for constructing thioethers under metal-free conditions.

Building upon these studies and considering our previous successes in using metal triflates in catalysis^27,38,39^, we decided to thoroughly investigate their application in thioetherification. Initially, we hypothesized that simple HOTf^40,41^ should serve as an equally effective catalyst for this process, thereby excluding the need for d-block metals. This allowed us to develop a highly efficient method for thioether synthesis. Furthermore, this work addresses a significant practicality concern by presenting a heterogeneous alternative^42,43^ in the form of commercially available Nafion^44,45^. Additionally, the aspect of hidden Brønsted acid catalysis is discussed in the context of metal triflates application^46–49^.

## Methods

### General information

All reactions were carried out in the ambient atmosphere. Solvents used for all experiments were purchased from Honeyweel or Sigma Aldrich (Merck), and used as received. Triflic acid was purchased from ABCR GmBH. Metal triflates and NAFION® (in the form of pellets) were purchased from Sigma Aldrich (Merck). Commercially available thiols and alcohols were purchased from Sigma Aldrich (Merck), Angene or Ambeed, and used as received. The progress of reactions (conversion of thiols) was monitored by GC chromatography using Bruker Scion 460-GC and Agilent 5977B GC/MSD with Agilent 8860 GC System. The structures of products were determined by NMR spectroscopy, IR spectroscopy, and MS spectrometry. The ^1^H NMR (400 or 600 MHz), and ^13^C NMR (101 or 151 MHz) spectra were recorded on Bruker Avance III HD NanoBay spectrometer, using chloroform-d (CDCl_3_) as the solvent. Deuterated solvents were purchased from Sigma Aldrich (Merck) (CDCl_3_ 99.8 atom% D) and used as received. The enantiomeric purity was determined by HPLC analysis (Daicel Chiralcel OD-H). FT- IR spectra were taken on a Nicolet™ iS50 FTIR Spectrometer. In the case of IR spectroscopy in real-time, the measurements were made using a ReactIR 15 Mettler Tolledo spectrophotometer, equipped with a 9-reflection probe with a diamond window of 9.5 mm AgX DiComp Mettler Tolledo and an MCT detector cooled with nitrogen.

### General synthetic procedures

All procedures can be also found in Supporting Information. Here, we present representative procedures.

#### The synthesis of compounds 3a–3p and 3aa–3af

To a 10 mL vial equipped with a magnetic stirring bar, alcohol (**1**, 1 mmol), thiol (**2**, 1 mmol), nitromethane (1 mL), and HOTf (0.01 mmol) were added under an ambient atmosphere. Subsequently, the reaction mixture was stirred at 80 °C for 2 h. After the reaction was completed, in order to neutralize HOTf the potassium carbonate (0.01 mmol) was added. After this time, the solvent was evaporated under reduced pressure. Next, the crude products were separated via extraction (diethyl ether-water), to give corresponding products **3a**–**3p**, and **3aa**–**3af**. The pure products were identified by ^1^H NMR, ^13^C NMR, IR, and MS spectrometry.

#### The synthesis of compounds 3q and 3r

To a 10 mL vial equipped with a magnetic stirring bar, alcohol (**1**, 2 mmol), thiol (**2**, 1 mmol), nitromethane (1 mL), and HOTf (0.02 mmol) were added under an ambient atmosphere. Subsequently, the reaction mixture was stirred at 80 °C for 2 h. After the reaction was completed, in order to neutralize HOTf the potassium carbonate (0.02 mmol) was added. After this time, the solvent was evaporated under reduced pressure. Next, the crude products were separated via extraction (diethyl ether-water), to give corresponding products **3q**–**3r**. The pure products were identified by ^1^H NMR, ^13^C NMR, IR, and MS spectrometry.

#### The synthesis of compounds 3ag-3am, and 3ao-3ap

To a 10 mL vial equipped with a magnetic stirring bar, alcohol (**1**, 1 mmol), thiol (**2**, 1 mmol), nitromethane (1 mL), and HOTf (0.05 mmol) were added under an ambient atmosphere. Subsequently, the reaction mixture was stirred at 80 °C for a definite time (2–6 h). After the reaction was completed, in order to neutralize HOTf the potassium carbonate (0.01 mmol) was added. After this time, the solvent was evaporated under reduced pressure. Next, the crude products were separated via extraction (diethyl ether-water), to give corresponding products **3**ag-**3am, and 3ao-3ap**. The pure products were identified by ^1^H NMR, ^13^C NMR, IR, and MS spectrometry.

## Results and discussion

The optimization studies, presented in Table 1, involved an in-depth investigation of a dehydrative coupling reaction between thiophenol (**2a**) and tert-amyl alcohol (**1a**). Significantly, all experiments were carried out utilizing new vials and magnetic stirrers. This meticulous approach holds immense significance in eradicating any potential impact stemming from trace amounts of other transition metal impurities^50,51^. Since the substrates are not air-sensitive, we performed all reactions under an ambient atmosphere. Importantly, comprehensive details related to optimization studies (especially the use of metal triflates as the catalysts) can be found in the Supporting Information file (Table S1).

**Table 1:** Optimization studies for a metal-free dehydrative thioetherification of alcohols^a^.


Entry	Variation of standard condition	Conversion of **2a** (%)^b^	Selectivity (%)^d^ (**3a**): (**4a**)
1	No change	99 (90)^c^	100: 0
2	0.5 mol% of HOTf	93	100: 0
3	No catalyst	0	–
4	Under solvent-free conditions	75	85: 15^e^
5	At rt instead of 80 °C	25^f^	94: 6
6	At 60 °C instead of 80 °C	45^f^	95: 5
7	In(OTf)_3_^g^ instead of HOTf	99 (91)^c^	100: 0
8	Cu(OTf)_2_^g^ instead of HOTf	97 (90)^c^	100: 0
9	In acetonitrile	25^h^	100: 0^i^
10	In 2-MeTHF	5^h^	100: 0^i^
11	In water	10^h^	0: 100

^a^General reaction conditions: **1a** (1 eq.), **2a** (1 eq.), HOTf (1 mol%), CH_3_NO_2_ (1 mL), under an ambient atmosphere, 80 °C, 2 h.

^b^Conversion of **2a** determined by GC.

^c^Isolated yield.

^d^Selectivity of [sulfide]:[disulfide] products determined by GC.

^e^There was observed a mixture of three different thioethers, in ratio 15:53(**3a**):32.

^f^After 20 h.

^g^0.5 mol% of metal triflate.

^h^After 2 h.

^i^Mixture of isomers of three different thioethers.

Initially, we tested the activity and selectivity of simple HOTf in a dehydrative coupling reaction (Table 1). The optimal attempt was conducted in CH_3_NO_2_ as a solvent (80 °C, 2 h), in the presence of 1 mol% of HOTf. As a result, we obtained the desired product **3a** in 90% yield (entry 1). Attempt to decrease the loading of the catalyst gave a bit inferior conversion (entry 2). The control reaction revealed that the process does not proceed without the catalyst (entry 3). Instead, it was found that the reaction works under solvent-free conditions. However, in such a case we observed the formation of three different thioethers (entry 4). Finally, several solvents including H_2_O, 2-MeTHF, and CH_3_CN were utilized (entries 9–11). We demonstrated that nitromethane was the solvent of choice (other mediums were less efficient and provided noticeably lower chemoselectivity). The above-mentioned processes can also proceed in the presence of commercially available metal triflates (for details please see Table 1, entry 7 and 8, as well as SI, Table S1).

**Figure 2:**
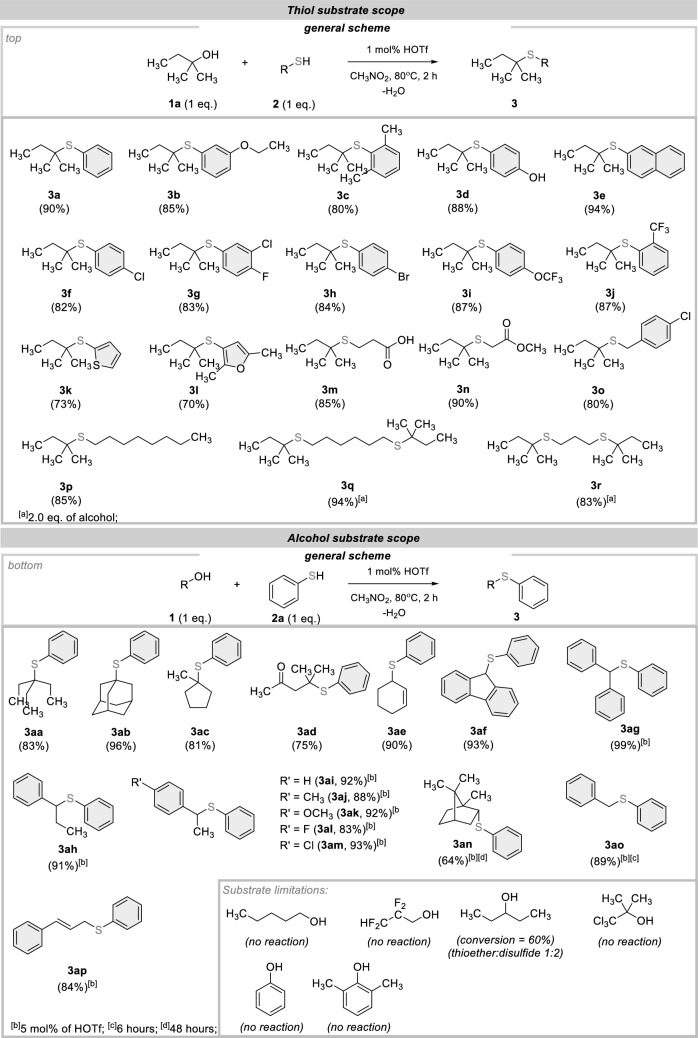
Substrate scope for dehydrative coupling reaction between alcohols and thiols.

Having the optimized conditions at our disposal, we conducted tests using various thiols to demonstrate the broad applicability of our protocol (Fig. 2, top). The reaction conditions proved to be effective for a diverse set of thiophenol derivatives (**3a**–**3j**). As an initial example **3a**, benzenethiols bearing electron-donating groups were readily alkylated (**3b**–**3e**), including difunctional 4-hydroxythiophenol **3d** (88% yield). Gratifyingly, halogenated thiophenols were readily adopted in this protocol (**3f–3h**, 82–84% yield), as were electron-deficient ones bearing trifluoromethyl and trifluoromethoxy functionalities (**3i**–**3j**, 87% yield). Motivated by these findings, we subsequently explored the utilization of heterocyclic thiols, which serve as biorelevant frameworks. Each of them yielded the desired products with moderate efficiencies (**3k**–**3l**, 70–73% yields). Next, we were pleased to find that HS-terminated carboxylic acids and esters were also successfully alkylated under standard conditions leading to products **3m**–**3n** in very good yields (85–90%).

Subsequently, we tested our methodology on variously substituted aliphatic mercaptans. Simple 1-octanethiol, as well as chloro-substituted benzylthiol reacted well, providing **3o** and **3p** in good yields (80–85%). Next, we sought to obtain alkylated bis(thioethers). Using our catalytic system, two dialkylated symmetrical variants were afforded (**3q** and **3r**, in 83% and 94% yields).

Following the high efficiency and chemoselectivity of the transformation, we proceeded to advance the exploration of additional applications for our catalytic system. Encouragingly, this strategy also enabled the dehydrative coupling of other alcohols with benzenethiol. As shown in Fig. 2 (bottom), another tertiary alcohol such as 3-ethyl-3-pentanol (**1b**), 1-adamantanol (**1c**), 1-methylcyclopentanol (**1d**), and 4-hydroxy-4-methyl-2-pentanone (**1e**) were exclusively converted into valuable sulfides (**3aa**–**3ad**). In the last example, the ketone group remains untouched, which is particularly noteworthy (**3ad**). When the reaction is catalyzed by Lewis acids, it results in the formation of a dithioacetal. Furthermore, commercial secondary alcohols also participated effectively in this reaction (**3ae**–**3an**, 64–99% yield), while in the case of 2-cyclohexen-1-ol (**1f**) preserving the ene-functionality untouched. Regarding the more complex compound, we tested enantiomerically pure (-)-borneol, which is used, among other things, in pharmaceuticals and fragrances. The reaction required a longer time (48 h), but led only to the expected *endo*-diastereoisomer (**3an**, 64% yield). Finally, two primary (benzylic and allylic) alcohols were also readily adopted in this protocol (**3ao**–**3ap**, 84–89% yield).

**Figure 3:**
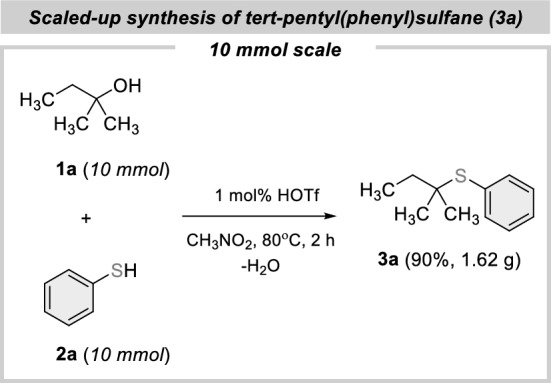
Scaled-up synthesis of **3a**.

Lastly, the reaction on a larger scale demonstrated the robustness and preparative scale utility of the process, under procedurally convenient conditions (Fig. 3).

**Figure 4:**
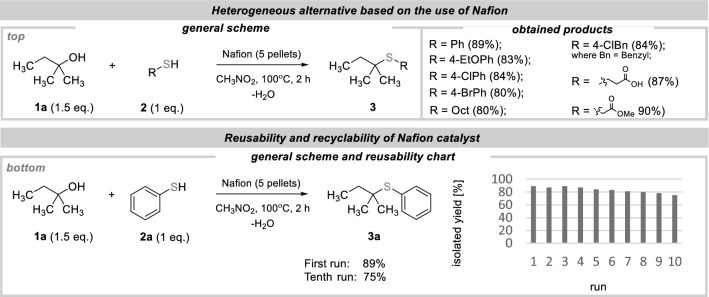
Dehydrative thioetherification of alcohols in the presence of Nafion catalyst.

The concept of Brønsted acid catalysis mentioned above motivated us to explore a heterogeneous system using Nafion®, a perfluorinated copolymer with sulfonic acid groups. This particular copolymer has proven useful in dehydration and esterification reactions^52–54^. In our experiment, we initially selected benzenethiol (1 mmol) and tert-amyl alcohol (1.5 mmol) as the coupling partners. We conducted the reaction using approximately 0.22 g of Nafion® NR50 pellets (approx. 5 pellets) (Fig. 4, top). To obtain a detailed understanding of the heterogeneous catalyst, we became intrigued to further probe its reusable nature (Fig. 4, bottom). We were hence delighted to observe that Nafion was successfully reused without a significant loss of performance over 10 cycles.

**Figure 5:**
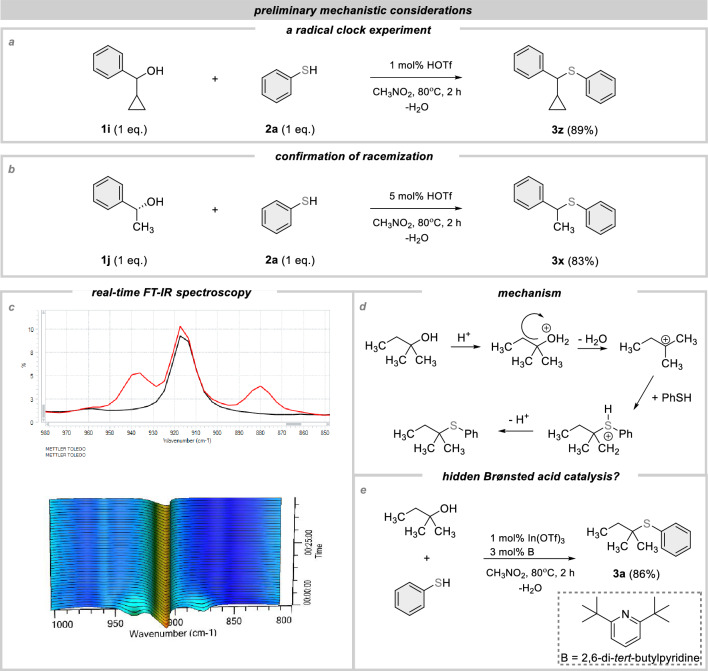
Mechanistic studies.

Next, to get some mechanistic insights into Brønsted acid catalysis, we carried out some of preliminary experiments (Fig. 5). As a first investigation, we conducted a radical clock experiment. It gives the desired product containing an untouched cyclopropyl ring (**3z**), thereby implying that radical pathways were likely, not operative (Fig. 5a). Therefore, the most probable mechanism involves the S_N_1-type nucleophilic substitution. With the formation of a planar *sp*^2^ hybridized carbocation, racemization occurs.

Confirmation of this reaction pathway is provided by using an R-enantiomer of 1-phenylethan-1-ol. As a result, a racemic mixture was observed (Fig. 5b). Next, we monitored the dehydration reaction using reactIR (Fig. 5c). The kinetic plots obtained for the coupling of tert-amyl alcohol (**1a**) to thiophenol (**2a**) confirmed a rapid disappearance of the distinguishing bands at 880 and 940 cm^−1^ (Fig. 5c). Thus, a mechanism is presented in Fig. 5d. Finally, we proceeded to find an answer to the question of whether this reaction can occur using both Brønsted and Lewis catalysis. In this case, the hydrolysis of metal triflates is known to occur with the formation of triflic acid. Therefore, we conducted a test reaction using indium(III) triflate as the catalyst. However, we carried it out in the presence of 2,6-di-tert-butylpyridine, which does not coordinate to Lewis acids but readily undergoes protonation. Despite the use of pyridine, the reaction proceeded unchanged in terms of efficiency and selectivity, suggesting that both Brønsted and Lewis acids can catalyze this reaction (Fig. 5e).

## Conclusions

In summary, we have successfully developed an innovative metal-free approach for thioesterification of alcohols utilizing thiols. Unlike previous methods that heavily relied on transition metals or metal triflates, our new method offers distinct advantages. Through our studies, we have demonstrated that a simple triflic acid serves as a highly efficient and selective catalyst. Moreover, we have addressed practicality concerns by exploring the option of heterogeneous catalysis using superacidic Nafion. This exciting development allows for the catalyst's repeated usage without significant loss in process efficiency. Furthermore, our research has provided valuable insights into the reaction mechanism. In addition, we conducted a comparison between Lewis acid and Brønsted acid catalysis, revealing that both approaches exhibit equally remarkable effectiveness. These significant advancements open up exciting possibilities for sustainable and green thioesterification processes, with reduced environmental impact and enhanced applicability in industrial and pharmaceutical contexts.

### Supplementary Information

Supplementary Information 1.

## Data Availability

The datasets used and/or analyzed during the current study are available from the corresponding author on reasonable request.
