# Supplementary material for: Access to thioethers from thiols and alcohols via homogeneous and heterogeneous catalysis
Source: Sci Rep. 2023 Nov 23;13:20624. doi: 10.1038/s41598-023-47938-4 (PMC10667213; doi:10.1038/s41598-023-47938-4)

**Access to thioethers from thiols and alcohols via homogeneous and heterogeneous catalysis**

Martyna Markwitz,^a^ Klaudiusz Labrzycki,^a^ Laura Azcune,^b^ Aitor Landa^b^ and Krzysztof Kuciński^a^,*

^a^Faculty of Chemistry, Adam Mickiewicz University, Poznan; Uniwersytetu Poznanskiego St. 8, 61-614 Poznan (Poland);

^b^Department of Organic Chemistry I, Faculty of Chemistry, University of the Basque Country UPV/EHU, Paseo Manuel Lardizábal 3, 20018 San Sebastián (Spain);

*email: kucinski.k@amu.edu.pl;

**Supporting Information**

**CONTENT**

[GENERAL INFORMATION 4](#_Toc147304279)

[OPTIMIZATION OF REACTION CONDITIONS 5](#_Toc147304280)

[SYNTHETIC PROCEDURES 6](#_Toc147304281)

[CHARACTERIZATION DATA FOR ALL PRODUCTS 8](#_Toc147304282)

[Tert-pentyl(phenyl)sulfane (3a) 8](#_Toc147304283)

[(3-Ethoxyphenyl)(tert-pentyl)sulfane (3b) 9](#_Toc147304284)

[(2,6-Dimethylphenyl)(tert-pentyl)sulfane (3c) 9](#_Toc147304285)

[4-(tert-Pentylthio)phenol (3d) 9](#_Toc147304286)

[Naphthalen-2-yl(tert-pentyl)sulfane (3e) 10](#_Toc147304287)

[(4-Chlorophenyl)(tert-pentyl)sulfane (3f) 10](#_Toc147304288)

[(3-Chloro-4-fluorophenyl)(tert-pentyl)sulfane (3g) 10](#_Toc147304289)

[(4-Bromophenyl)(tert-pentyl)sulfane (3h) 11](#_Toc147304290)

[tert-Pentyl(4-(trifluoromethoxy)phenyl)sulfane (3i) 11](#_Toc147304291)

[tert-Pentyl(2-(trifluoromethyl)phenyl)sulfane (3j) 11](#_Toc147304292)

[2-(tert-Pentylthio)thiophene (3k) 12](#_Toc147304293)

[2-(tert-Pentylthio)thiophene (3l) 12](#_Toc147304294)

[3-(tert-Pentylthio)propanoic acid (3m) 12](#_Toc147304295)

[Methyl 2-(tert-pentylthio)acetate (3n) 13](#_Toc147304296)

[(4-Chlorobenzyl)(tert-pentyl)sulfane (3o) 13](#_Toc147304297)

[Octyl(tert-pentyl)sulfane (3p) 13](#_Toc147304298)

[1,6-Bis(tert-pentylthio)hexane (3q) 14](#_Toc147304299)

[1,3-Bis(tert-pentylthio)propane (3r) 14](#_Toc147304300)

[(3-Ethylpentan-3-yl)(phenyl)sulfane (3aa) 14](#_Toc147304301)

[((3s,5s,7s)-adamantan-1-yl)(phenyl)sulfane (3ab) 15](#_Toc147304302)

[(1-Methylcyclopentyl)(phenyl)sulfane (3ac) 15](#_Toc147304303)

[4-Methyl-4-(phenylthio)pentan-2-one (3ad) 15](#_Toc147304304)

[Cyclohex-2-en-1-yl(phenyl)sulfane (3ae) 16](#_Toc147304305)

[(9H-fluoren-9-yl)(phenyl)sulfane (3af) 16](#_Toc147304306)

[Benzhydryl(phenyl)sulfane (3ag) 16](#_Toc147304307)

[Phenyl(1-phenylpropyl)sulfane (3ah) 17](#_Toc147304308)

[Phenyl(1-phenylethyl)sulfane (3ai) 17](#_Toc147304309)

[Phenyl(1-(p-tolyl)ethyl)sulfane (3aj) 18](#_Toc147304310)

[(1-(4-Methoxyphenyl)ethyl)(phenyl)sulfane (3ak) 18](#_Toc147304311)

[(1-(4-Fluorophenyl)ethyl)(phenyl)sulfane (3al) 18](#_Toc147304312)

[(1-(4-Chlorophenyl)ethyl)(phenyl)sulfane (3am) 19](#_Toc147304313)

[Phenyl((1S,2R,4S)-1,7,7-trimethylbicyclo[2.2.1]heptan-2-yl)sulfane (3an) 19](#_Toc147304314)

[Benzyl(phenyl)sulfane (3ao) 19](#_Toc147304315)

[Cinnamyl(phenyl)sulfane (3ap) 20](#_Toc147304316)

[(Cyclopropyl(phenyl)methyl)(phenyl)sulfane (3z) 20](#_Toc147304317)

[SPECTRA FOR ALL PRODUCTS 22](#_Toc147304318)

[Tert-pentyl(phenyl)sulfane (3a) 22](#_Toc147304319)

[(3-Ethoxyphenyl)(tert-pentyl)sulfane (3b) 23](#_Toc147304320)

[(2,6-Dimethylphenyl)(tert-pentyl)sulfane (3c) 24](#_Toc147304321)

[4-(tert-Pentylthio)phenol (3d) 25](#_Toc147304322)

[Naphthalen-2-yl(tert-pentyl)sulfane (3e) 26](#_Toc147304323)

[(4-Chlorophenyl)(tert-pentyl)sulfane (3f) 27](#_Toc147304324)

[(3-Chloro-4-fluorophenyl)(tert-pentyl)sulfane (3g) 28](#_Toc147304325)

[(4-Bromophenyl)(tert-pentyl)sulfane (3h) 30](#_Toc147304326)

[tert-Pentyl(4-(trifluoromethoxy)phenyl)sulfane (3i) 31](#_Toc147304327)

[tert-Pentyl(2-(trifluoromethyl)phenyl)sulfane (3j) 33](#_Toc147304328)

[2-(tert-Pentylthio)thiophene (3k) 35](#_Toc147304329)

[2-(tert-Pentylthio)thiophene (3l) 36](#_Toc147304330)

[3-(tert-Pentylthio)propanoic acid (3m) 37](#_Toc147304331)

[Methyl 2-(tert-pentylthio)acetate (3n) 38](#_Toc147304332)

[(4-Chlorobenzyl)(tert-pentyl)sulfane (3o) 39](#_Toc147304333)

[Octyl(tert-pentyl)sulfane (3p) 40](#_Toc147304334)

[1,6-Bis(tert-pentylthio)hexane (3q) 41](#_Toc147304335)

[1,3-Bis(tert-pentylthio)propane (3r) 42](#_Toc147304336)

[(3-Ethylpentan-3-yl)(phenyl)sulfane (3aa) 43](#_Toc147304337)

[((3s,5s,7s)-adamantan-1-yl)(phenyl)sulfane (3ab) 44](#_Toc147304338)

[(1-Methylcyclopentyl)(phenyl)sulfane (3ac) 45](#_Toc147304339)

[4-Methyl-4-(phenylthio)pentan-2-one (3ad) 46](#_Toc147304340)

[Cyclohex-2-en-1-yl(phenyl)sulfane (3ae) 47](#_Toc147304341)

[(9H-fluoren-9-yl)(phenyl)sulfane (3af) 48](#_Toc147304342)

[Benzhydryl(phenyl)sulfane (3ag) 49](#_Toc147304343)

[Phenyl(1-phenylpropyl)sulfane (3ah) 50](#_Toc147304344)

[Phenyl(1-phenylethyl)sulfane (3ai) 51](#_Toc147304345)

[Phenyl(1-(p-tolyl)ethyl)sulfane (3aj) 52](#_Toc147304346)

[(1-(4-Methoxyphenyl)ethyl)(phenyl)sulfane (3ak) 53](#_Toc147304347)

[(1-(4-Fluorophenyl)ethyl)(phenyl)sulfane (3al) 54](#_Toc147304348)

[(1-(4-Chlorophenyl)ethyl)(phenyl)sulfane (3am) 56](#_Toc147304349)

[Phenyl((1S,2R,4S)-1,7,7-trimethylbicyclo[2.2.1]heptan-2-yl)sulfane (3an) 57](#_Toc147304350)

[Benzyl(phenyl)sulfane (3ao) 58](#_Toc147304351)

[Cinnamyl(phenyl)sulfane (3ap) 59](#_Toc147304352)

[(Cyclopropyl(phenyl)methyl)(phenyl)sulfane (3z) 60](#_Toc147304353)

# GENERAL INFORMATION

All reactions were carried out in the ambient atmosphere. Solvents used for all experiments were purchased from Honeyweel or Sigma Aldrich (Merck), and used as received. Triflic acid was purchased from ABCR GmBH. Metal triflates and NAFION® (in the form of pellets) were purchased from Sigma Aldrich (Merck). Commercially available thiols and alcohols were purchased from Sigma Aldrich (Merck), Angene or Ambeed, and used as received. The progress of reactions (conversion of thiols) was monitored by GC chromatography using Bruker Scion 460-GC and Agilent 5977B GC/MSD with Agilent 8860 GC System. The structures of products were determined by NMR spectroscopy, IR spectroscopy, and MS spectrometry. The ^1^H NMR (400 or 600 MHz), and ^13^C NMR (101 or 151 MHz) spectra were recorded on Bruker Avance III HD NanoBay spectrometer, using chloroform-d (CDCl_3_) as the solvent. Deuterated solvents were purchased from Sigma Aldrich (Merck) (CDCl_3_ 99.8 atom% D) and used as received. The enantiomeric purity was determined by HPLC analysis (Daicel Chiralcel OD-H). FT- IR spectra were taken on a Nicolet™ iS50 FTIR Spectrometer. In the case of IR spectroscopy in real-time, the measurements were made using a ReactIR 15 Mettler Tolledo spectrophotometer, equipped with a 9-reflection probe with a diamond window of 9.5 mm AgX DiComp Mettler Tolledo and an MCT detector cooled with nitrogen.

# OPTIMIZATION OF REACTION CONDITIONS

**Table S1****.** Optimization studies for a metal-free dehydrative thioetherification of alcohols.^[a]^

| Entry | Variation of standard condition | Conversion of **2a** [%]^[b]^ | Selectivity [%]^[d]^  [**3a**] : [**4a**] |
| --- | --- | --- | --- |
| 1 | no change | 99 (90)^[c]^ | 100 : 0 |
| 2 | 0.5 mol% of HOTf | 93 | 100 : 0 |
| 3 | no catalyst | 0 | - |
| 4 | under solvent-free conditions | 75 | 85:15^[e]^ |
| 5 | at rt instead of 80°C | 25^[f]^ | 94:6 |
| 6 | at 60°C instead of 80°C | 45^[f]^ | 95:5 |
| 7 | In(OTf)_3_^[g]^ instead of HOTf | 99 (91)^[c]^ | 100:0 |
| 8 | Cu(OTf)_2_^[g]^ instead of HOTf | 97 (90)^[c]^ | 100:0 |
| 9 | Ni(OTf)_2_^[g]^ instead of HOTf | 70^[c]^ | 100:0 |
| 10 | AgOTf^[g]^ instead of HOTf | 94^[c]^ | 100:0 |
| 11 | Sc(OTf)_3_^[g]^ instead of HOTf | 95^[c]^ | 100:0 |
| 12 | Zn(OTf)_2_^[g]^ instead of HOTf | 49^[c]^ | 100:0 |
| 13 | in acetonitrile | 25^[h]^ | 100 : 0^[i]^ |
| 14 | in 2-MeTHF | 5^[h]^ | 100 : 0^[i]^ |
| 15 | in water | 10^[h]^ | 0 : 100 |

^[a]^General reaction conditions: **1a** (1 eq.), **2a** (1 eq.), HOTf (1 mol%), CH_3_NO_2_ (1 mL), under an ambient atmosphere, 80°C, 2 h. ^[b]^Conversion of **2a** determined by GC. ^[c]^Isolated yield. ^[d]^Selectivity of [sulfide]:[disulfide] products determined by GC. ^[e]^There was observed a mixture of three different thioethers, in ratio 15:53(**3a**):32. ^[f]^After 20 h. ^[g]^0.5 mol% of metal triflate. ^[h]^After 2 h. ^[i]^Mixture of isomers of three different thioethers.

# SYNTHETIC PROCEDURES

**The synthesis of compounds 3a-3p, 3aa-3af, and 3ap**

To a 10 mL vial equipped with a magnetic stirring bar, alcohol (**1**, 1 mmol), thiol (**2**, 1 mmol), nitromethane (1 mL), and triflic acid (0.01 mmol) were added under an ambient atmosphere. Subsequently, the reaction mixture was stirred at 80°C for 2 h. After the reaction was completed, in order to neutralize triflic acid the potassium carbonate (0.01 mmol) was added. After this time, the solvent was evaporated under reduced pressure. Next, the crude products were separated *via* extraction (diethyl ether-water), to give corresponding products **3a**-**3p**, **3aa**-**3af,** and **3ap**. The pure products were identified by ^1^H NMR, ^13^C NMR, IR, and MS spectrometry.

**The synthesis of compounds 3q and 3r**

To a 10 mL vial equipped with a magnetic stirring bar, alcohol (**1**, 2 mmol), thiol (**2**, 1 mmol), nitromethane (1 mL), and triflic acid (0.02 mmol) were added under an ambient atmosphere. Subsequently, the reaction mixture was stirred at 80°C for 2 h. After the reaction was completed, in order to neutralize triflic acid the potassium carbonate (0.02 mmol) was added. After this time, the solvent was evaporated under reduced pressure. Next, the crude products were separated *via* extraction (diethyl ether-water), to give corresponding products **3q**-**3r**. The pure products were identified by ^1^H NMR, ^13^C NMR, IR, and MS spectrometry.

**The synthesis of compounds 3ag-3am, and 3ao**

To a 10 mL vial equipped with a magnetic stirring bar, alcohol (**1**, 1 mmol), thiol (**2**, 1 mmol), nitromethane (1 mL), and triflic acid (0.05 mmol) were added under an ambient atmosphere. Subsequently, the reaction mixture was stirred at 80°C for a definite time (2-6 h). After the reaction was completed, in order to neutralize triflic acid the potassium carbonate (0.01 mmol) was added. After this time, the solvent was evaporated under reduced pressure. Next, the crude products were separated *via* extraction (diethyl ether-water), to give corresponding products **3ag-3am, and 3ao**. The pure products were identified by ^1^H NMR, ^13^C NMR, IR, and MS spectrometry.

**The synthesis of compounds 3an**

To a 10 mL vial equipped with a magnetic stirring bar, (-)-borneol (**1o**, 1 mmol), thiophenol (**2a**, 1 mmol), nitromethane (1 mL), and triflic acid (0.05 mmol) were added under an ambient atmosphere. Subsequently, the reaction mixture was stirred at 80°C for a definite time (48 h). After the reaction was completed, in order to neutralize triflic acid the potassium carbonate (0.01 mmol) was added. After this time, the solvent was evaporated under reduced pressure. The final crude product **3an** was purified by silica gel flash column chromatography (eluent: Hex 100%). The pure product was identified by ^1^H NMR, ^13^C NMR, IR, and MS spectrometry.

**Scaled-up synthesis of 3a**

To a 10 mL vial equipped with a magnetic stirring bar, tert-amyl alcohol (**1a**, 10 mmol), thiophenol (**2a**, 10 mmol), nitromethane (10 mL), and triflic acid (0.01 mmol) were added under an ambient atmosphere. Subsequently, the reaction mixture was stirred at 80°C for 2 h. After the reaction was completed, in order to neutralize triflic acid the potassium carbonate (0.01 mmol) was added. After this time, the solvent was evaporated under reduced pressure. Next, the crude product was separated *via* extraction (diethyl ether-water), to give corresponding product **3z** (1.62 g (90%). The pure product was identified by ^1^H NMR, ^13^C NMR, IR, and MS spectrometry.

**The synthesis of compounds 3a, 3b, 3f, 3h, and 3m-3p by using Nafion**

To a 10 mL vial equipped with a magnetic stirring bar, alcohol (**1**, 1.5 mmol), thiophenol (**2a**, 1 mmol), nitromethane (1 mL), and Nafion pellets (5 pellets, ~0.22 g) were added under an ambient atmosphere. Subsequently, the reaction mixture was stirred at 100°C for 2 h. After this time, the reaction mixture was separated from the catalyst and all volatiles were evaporated under reduced pressure. Next, the crude products were separated *via* extraction (diethyl ether-water), to give corresponding products **3a, 3b, 3f, and 3h**. The pure products were identified by ^1^H NMR, ^13^C NMR, IR, and MS spectrometry.

**The synthesis of compounds 3z**

To a 10 mL vial equipped with a magnetic stirring bar, α-cyclopropylbenzyl alcohol (**1i**, 1 mmol), thiophenol (**2a**, 1 mmol), nitromethane (1 mL), and triflic acid (0.01 mmol) were added under an ambient atmosphere. Subsequently, the reaction mixture was stirred at 80°C for 2 h. After the reaction was completed, in order to neutralize triflic acid the potassium carbonate (0.01 mmol) was added. After this time, the solvent was evaporated under reduced pressure. Next, the crude product was separated *via* extraction (diethyl ether-water), to give corresponding product **3z**. The pure product was identified by ^1^H NMR, ^13^C NMR, IR, and MS spectrometry.

**The racemization test**

(R)-1-phenylethan-1-ol **1j** (1 mmol, 122.6 mg, 0.12 ml) and benzenethiol **2a** (1 mmol, 110.2 mg, 0.10 ml) were added to 1 mL of CH_3_NO_2_. To the corresponding solution, 5 mol% of HOTf (0.05 mmol, 4.4 µl) was added and the mixture was stirred at 80 ºC for 2 hours (the reaction was monitored by NMR). The crude product was purified by silica gel flash column chromatography (eluent: Hex 100%) to obtain phenyl(1-phenylethyl)sulfane **3x** as a colourless liquid (yield 83%, 177.9 mg). The enantiomeric purity was determined by HPLC analysis (Daicel Chiralcel OD-H, hexane/isopropanol 100:0, flow rate= 0.6 mL/min, retention times: 24.4 min (major) and 28.3 min (minor).

# CHARACTERIZATION DATA FOR ALL PRODUCTS

## Tert-pentyl(phenyl)sulfane (3a)

Tert-pentyl(phenyl)sulfane was obtained as a colorless oil in 90% yield.

**^1^H NMR:** (400 MHz, CDCl_3_) δ 7.57 – 7.51 (m, 2H), 7.36 – 7.30 (m, 3H), 1.53 (q, *J* = 7.4 Hz, 2H), 1.24 (s, 6H), 1.03 (t, *J* = 7.4 Hz, 3H).

**^13^C NMR:** (101 MHz, CDCl_3_) δ 137.3, 132.2, 128.3, 128.2, 49.5, 34.6, 28.0, 9.0.

**IR: (neat)** vmax cm^-1^:2965, 2920, 1473, 1437, 1362, 1156, 1066, 1007, 799, 692.

**EI-MS m/z (rel. int.):** 180 (M^+^, 10%), 110 (100), 71 (10), 55 (20).

## (3-Ethoxyphenyl)(tert-pentyl)sulfane (3b)

(3-Ethoxyphenyl)(tert-pentyl)sulfane was obtained as a colorless oil in 85% yield.

**^1^H NMR:** (400 MHz, CDCl_3_) δ 7.13 (t, *J* = 7.9 Hz, 1H), 7.03 – 6.97 (m, 2H), 6.81 (ddd, *J* = 8.3, 2.6, 1.1 Hz, 1H), 3.95 (q, *J* = 7.0 Hz, 2H), 1.45 (q, *J* = 7.4 Hz, 2H), 1.34 (t, *J* = 7.0 Hz, 3H), 1.15 (s, 6H), 0.93 (t, *J* = 7.4 Hz, 3H).

**^13^C NMR:** (101 MHz, CDCl_3_) δ 159.1, 133.9, 130.1, 129.5, 123.6, 115.6, 64.0, 50.3, 35.3, 28.8, 15.3, 9.7.

**IR: (neat)** vmax cm^-1^: 2969, 2877, 1587, 1462, 1388, 1280, 1008, 940, 878, 603.

**EI-MS m/z (rel. int.):** 224 (M^+^, 35%), 154 (100), 126 (55), 97 (40).

## (2,6-Dimethylphenyl)(tert-pentyl)sulfane (3c)

(2,6-Dimethylphenyl)(tert-pentyl)sulfane was obtained as a colorless oil in 80% yield.

**^1^H NMR:** (400 MHz, Chloroform-d) δ 7.13 (s, 3H), 2.58 (s, 6H), 1.66 (q, *J* = 7.4 Hz, 2H), 1.20 (d, *J* = 2.0 Hz, 6H), 1.02 (t, *J* = 7.5 Hz, 3H).

**^13^C NMR:** (101 MHz, CDCl_3_) δ 145.57, 132.2, 128.4, 128.1, 53.1, 36.7, 28.6, 23.3, 9.5.

**IR: (neat)** vmax cm^-1^: 3054, 2963, 1578, 1456, 1155, 1054, 1130, 1009, 798, 724.

**EI-MS m/z (rel. int.):** 208 (M^+^, 5%), 138 (100), 105 (40), 91 (20).

## 4-(tert-Pentylthio)phenol (3d)

4-(tert-Pentylthio)phenol was obtained as a colorless oil in 88% yield.

**^1^H NMR:** (400 MHz, CDCl_3_) δ 7.35 – 7.22 (m, 2H), 6.74 – 6.66 (m, 2H), 5.44 (s, 1H), 1.40 (q, *J* = 7.4 Hz, 2H), 1.11 (s, 6H), 0.91 (t, *J* = 7.4 Hz, 3H).

**^13^C NMR:** (101 MHz, CDCl_3_) δ 156.4, 139.2, 133.0, 123.4, 116.3, 115.7, 49.7, 34.7, 28.1, 9.3.

**IR: (neat)** vmax cm^-1^: 3321, 2965, 1597, 1459, 1424, 1251, 1129, 1056, 1011, 640.

**EI-MS m/z (rel. int.):** 196 (M^+^, 20%), 126 (100), 97 (30), 55 (20).

## Naphthalen-2-yl(tert-pentyl)sulfane (3e)

Naphthalen-2-yl(tert-pentyl)sulfane was obtained as a solid in 94% yield.

**^1^H NMR:** (400 MHz, CDCl_3_) δ 8.09 – 8.03 (m, 1H), 7.89 – 7.74 (m, 3H), 7.58 (dd, *J* = 8.5, 1.8 Hz, 1H), 7.54 – 7.48 (m, 2H), 1.57 (q, *J* = 7.4 Hz, 2H), 1.27 (s, 6H), 1.05 (t, *J* = 7.4 Hz, 3H).

**^13^C NMR:** (101 MHz, CDCl_3_) δ 137.3, 134.5, 133.5, 133.2, 130.1, 128.0, 127.8, 127.7, 126.8, 126.4, 50.4, 35.1, 28.5, 9.4.

**IR: (neat)** vmax cm^-1^: 3321, 2965, 1597, 1459, 1424, 1251, 1129, 1056, 1011, 640.

**EI-MS m/z (rel. int.):** 230(M^+^, 20%), 160 (100), 115 (40), 55 (10).

## (4-Chlorophenyl)(tert-pentyl)sulfane (3f)

(4-Chlorophenyl)(tert-pentyl)sulfane was obtained as a colorless oil in 82% yield.

**^1^H NMR:** (400 MHz, CDCl_3_) δ 7.35 (d, *J* = 8.5 Hz, 2H), 7.21 (d, *J* = 8.4 Hz, 2H), 1.41 (q, *J* = 7.4 Hz, 2H), 1.12 (s, 6H), 0.92 (t, *J* = 7.4 Hz, 3H).

**^13^C NMR:** (101 MHz, CDCl_3_) δ 138.8, 135.2, 131.1, 128.8, 50.2, 34.9, 28.3, 9.3.

**IR: (neat)** vmax cm^-1^: 2966, 2922, 1571, 1474, 1385, 1156, 1093, 1055, 821, 746.

**EI-MS m/z (rel. int.):** 214 (M^+^, 10%), 144 (100), 108 (75), 71 (25).

## (3-Chloro-4-fluorophenyl)(tert-pentyl)sulfane (3g)

(3-Chloro-4-fluorophenyl)(tert-pentyl)sulfane was obtained as a colorless oil in 83% yield.

**^1^H NMR:** (400 MHz, CDCl_3_) δ 7.55 (dd, *J* = 7.1, 2.2 Hz, 1H), 7.37 (ddd, *J* = 8.5, 4.7, 2.2 Hz, 1H), 7.08 (t, *J* = 8.7 Hz, 1H), 1.50 (q, *J* = 7.4 Hz, 2H), 1.20 (s, 6H), 1.00 (t, *J* = 7.4 Hz, 3H).

**^13^C NMR:** (101 MHz, CDCl_3_) δ 158.79 (d, *J* = 251.4 Hz), 139.3, 137.47 (d, *J* = 7.2 Hz), 129.31 (d, *J* = 4.3 Hz), 120.85 (d, *J* = 17.9 Hz), 116.60 (d, *J* = 21.1 Hz), 50.4, 34.9, 28.2, 9.3.

**^19^F NMR**: (377 MHz, CDCl_3_) δ -115.04.

**IR: (neat)** vmax cm^-1^: 2968, 1581, 1374, 1258, 1156, 1095, 885, 717, 648, 558.

**EI-MS m/z (rel. int.):** 232 (M^+^, 20%), 162 (100), 126 (70), 71 (70).

## (4-Bromophenyl)(tert-pentyl)sulfane (3h)

(4-Bromophenyl)(tert-pentyl)sulfane was obtained as a colorless oil in 84% yield.

**^1^H NMR:** (400 MHz, CDCl_3_) δ 7.36 (d, *J* = 8.4 Hz, 2H), 7.28 (d, *J* = 8.4 Hz, 2H), 1.41 (q, *J* = 7.4 Hz, 2H), 1.12 (s, 6H), 0.92 (t, *J* = 7.4 Hz, 3H).

**^13^C NMR:** (101 MHz, CDCl_3_) δ 139.1, 131.8, 131.7, 123.5, 50.2, 34.9, 28.3, 9.3.

**IR: (neat)** vmax cm^-1^: 2965, 2921, 1567, 1466, 1382, 1174, 1130, 1070, 730, 567.

**EI-MS m/z (rel. int.):** 260 (M^+^,10%), 188 (100), 108 (80), 82 (20).

## tert-Pentyl(4-(trifluoromethoxy)phenyl)sulfane (3i)

tert-Pentyl(4-(trifluoromethoxy)phenyl)sulfane was obtained as a colorless oil in 87% yield.

**^1^H NMR:** (400 MHz, CDCl_3_) δ 7.57 – 7.49 (m, 2H), 7.18 – 7.12 (m, 2H), 1.50 (q, *J* = 7.4 Hz, 2H), 1.21 (s, 6H), 1.01 (t, *J* = 7.4 Hz, 3H).

**^13^C NMR:** (101 MHz, CDCl_3_) δ

**^19^F NMR:** (377 MHz, CDCl_3_) δ -57.8.

**IR: (neat)** vmax cm^-1^: 2970, 1591, 1461, 1382, 1092, 1018, 922, 850, 667, 561.

**EI-MS m/z (rel. int.):** 264 (M^+^,10%), 235 (5), 194 (100), 69 (40).

## tert-Pentyl(2-(trifluoromethyl)phenyl)sulfane (3j)

tert-Pentyl(2-(trifluoromethyl)phenyl)sulfane was obtained as a colorless oil in 87% yield.

**^1^H NMR:** (400 MHz, CDCl_3_) δ 7.77 – 7.64 (m, 2H), 7.52 – 7.39 (m, 2H), 1.68 (q, *J* = 7.4 Hz, 2H), 1.27 (s, 6H), 1.05 (t, *J* = 7.4 Hz, 3H).

**^13^C NMR:** (101 MHz, CDCl_3_) δ 139.5, 134.8 (q, *J* = 28.7 Hz), 133.05 (d, *J* = 1.6 Hz), 131.2, 128.3, 127.0 (q, *J* = 5.8 Hz), 123.5 (q), 52.1, 36.0, 28.8, 9.3.

**^19^F NMR:** (377 MHz, CDCl_3_) δ -58.3.

**IR: (neat)** vmax cm^-1^: 2968, 1592, 1469, 1379, 1256, 1110, 958, 765, 707, 598.

**EI-MS m/z (rel. int.):** 248 (M^+^, 40%), 177 (80), 157 (90), 71 (100).

## 2-(tert-Pentylthio)thiophene (3k)

2-(tert-Pentylthio)thiophene was obtained as a pale-yellow oil in 73% yield.

**^1^H NMR:** (400 MHz, CDCl_3_) δ 7.31 (dd, *J* = 5.4, 1.3 Hz, 1H), 7.05 (dd, *J* = 3.5, 1.4 Hz, 1H), 6.94 (dd, *J* = 5.4, 3.5 Hz, 1H), 1.45 (q, *J* = 7.4 Hz, 2H), 1.16 (s, 6H), 0.92 (t, *J* = 7.4 Hz, 3H).

**^13^C NMR:** (101 MHz, CDCl_3_) δ 137.3, 132, 131.1, 127.9, 51.0, 34.9, 28.2, 9.7.

**IR: (neat)** vmax cm^-1^: 2965, 2920, 1715, 1458, 1336, 1215, 1007, 848, 799, 575.

**EI-MS m/z (rel. int.):** 186 (M^+^, 15%), 116 (100), 71 (90), 55 (20).

## 2-(tert-Pentylthio)thiophene (3l)

2-(tert-Pentylthio)thiophene was obtained as a pale-yellow oil in 70% yield.

**^1^H NMR:** (400 MHz, CDCl_3_) δ 5.81 (s, 1H), 2.23 (s, 3H), 2.16 (s, 3H), 1.43 (q, *J* = 7.5 Hz, 2H), 1.11 (s, 6H), 0.90 (t, *J* = 7.4 Hz, 3H).

**^13^C NMR:** (101 MHz, CDCl_3_) δ 155.7, 149.5, 113.0, 108.7, 49.9, 34.8, 28.1, 13.6, 12.1, 9.4.

**IR: (neat)** vmax cm^-1^: 2966, 2920, 1713, 1569, 1362, 1334, 1158, 1005, 800, 655

## 3-(tert-Pentylthio)propanoic acid (3m)

3-(tert-Pentylthio)propanoic acid was obtained as a colorless oil in 85% yield.

**^1^H NMR:** (400 MHz, CDCl_3_) δ 2.77 – 2.67 (m, 2H), 2.60 (t, *J* = 7.3 Hz, 2H), 1.54 (q, *J* = 7.4 Hz, 2H), 1.25 (s, 6H), 0.93 (t, *J* = 7.4 Hz, 3H).

**^13^C NMR:** (101 MHz, CDCl_3_) δ 178.6, 46.4, 34.9, 34.8, 28.3, 22.5, 9.2.

**IR: (neat)** vmax cm^-1^: 2966, 2933, 1707, 1412, 1364, 1260, 1057, 1007, 930, 655.

## Methyl 2-(tert-pentylthio)acetate (3n)

Methyl 2-(tert-pentylthio)acetate was obtained as a colorless oil in 90% yield.

**^1^H NMR:** (400 MHz, CDCl_3_) δ 3.66 (s, 3H), 3.17 (s, 2H), 1.49 (q, *J* = 7.4 Hz, 2H), 1.20 (s, 6H), 0.88 (t, *J* = 7.4 Hz, 3H).

**^13^C NMR:** (101 MHz, CDCl_3_) δ 171.8, 52.5, 47.0, 34.7, 30.7, 28.1, 9.1.

**IR: (neat)** vmax cm^-1^: 2966, 1736, 1459, 1406, 1193, 1058, 1009, 896, 805, 584.

## (4-Chlorobenzyl)(tert-pentyl)sulfane (3o)

(4-Chlorobenzyl)(tert-pentyl)sulfane was obtained as a colorless oil in 80% yield.

**^1^H NMR:** (400 MHz, CDCl_3_) δ 7.41 – 7.17 (m, 4H), 3.68 (s, 2H), 1.60 (q, *J* = 7.4 Hz, 2H), 1.31 (s, 6H), 0.98 (t, *J* = 7.4 Hz, 3H).

**^13^C NMR:** (101 MHz, CDCl_3_) δ 137.3, 132.6, 130.4, 129.5, 128.9, 128.7, 46.9, 34.9, 32.3, 28.4, 28.4, 9.2.

**IR: (neat)** vmax cm^-1^: 2966, 2925, 1596, 1459, 1405, 1282, 1155, 1015, 833, 654.

**EI-MS m/z (rel. int.):** 228 (M^+^, 35%), 158 (30), 125 (100), 71 (55).

## Octyl(tert-pentyl)sulfane (3p)

Octyl(tert-pentyl)sulfane was obtained as a colorless oil in 85% yield.

**^1^H NMR:** (400 MHz, CDCl_3_) δ 2.51 – 2.35 (m, 2H), 1.60 – 1.47 (m, 4H), 1.42 – 1.32 (m, 2H), 1.32 – 1.19 (m, 14H), 0.93 (t, *J* = 7.5 Hz, 3H), 0.86 (t, *J* = 6.6 Hz, 3H).

**^13^C NMR:** (101 MHz, CDCl_3_) δ 45.5, 35.0, 32.0, 30.0, 29.5, 29.4, 29.3, 28.4, 27.8, 22.8, 14.2, 9.3.

**IR: (neat)** vmax cm^-1^: 2957, 2853, 1460, 1377, 1283, 1156, 1137, 1007, 806, 723.

**EI-MS m/z (rel. int.):** 216 (M^+^, 20%), 187 (30), 71 (100), 55 (45).

## 1,6-Bis(tert-pentylthio)hexane (3q)

1,6-Bis(tert-pentylthio)hexane was obtained as a colorless oil in 94% yield.

**^1^H NMR:** (400 MHz, CDCl_3_) δ 2.43 (t, *J* = 7.4 Hz, 4H), 1.63 – 1.46 (m, 8H), 1.38 (p, *J* = 3.5 Hz, 4H), 1.22 (s, 12H), 0.92 (t, *J* = 7.5 Hz, 6H).

**^13^C NMR:** (101 MHz, CDCl_3_) δ 45.6, 35. 0, 29.7, 29.1, 28.4, 27.7, 9.3.

**IR: (neat)** vmax cm^-1^: 2965, 2853, 1459, 1376, 1174, 1136, 1007, 911, 805, 771.

**EI-MS m/z (rel. int.):** 258 (50%), 125 (60), 105 (100), 72 (50).

## 1,3-Bis(tert-pentylthio)propane (3r)

1,3-Bis(tert-pentylthio)propane was obtained as a colorless oil in 83% yield.

**^1^H NMR:** (400 MHz, CDCl_3_) δ 2.53 (t, *J* = 7.2 Hz, 4H), 1.78 (p, *J* = 7.2 Hz, 2H), 1.51 (q, *J* = 7.5 Hz, 4H), 1.22 (s, 12H), 0.91 (t, *J* = 7.5 Hz, 6H).

**^13^C NMR:** (101 MHz, CDCl_3_) δ 45.8, 35.0, 30.1, 28.4, 27.1, 9.3.

**IR: (neat)** vmax cm^-1^: 2964, 2877, 1458, 1363, 1283, 1155, 1057, 805, 630, 584.

**EI-MS m/z (rel. int.):** 248 (M^+^, 20%), 177 (30), 71 (100), 55 (40).

## (3-Ethylpentan-3-yl)(phenyl)sulfane (3aa)

(3-Ethylpentan-3-yl)(phenyl)sulfane was obtained as a colorless oil in 83% yield.

**^1^H NMR:** (400 MHz, CDCl_3_) δ 7.43 – 7.36 (m, 2H), 7.26 – 7.17 (m, 3H), 1.31 (qd, *J* = 7.4, 1.2 Hz, 6H), 0.89 (td, *J* = 7.3, 1.2 Hz, 9H).

**^13^C NMR:** (101 MHz, CDCl_3_) δ 137.4, 132.4, 128.5, 128.5, 58.2, 28.0, 8.2.

**IR: (neat)** vmax cm^-1^: 3074, 2936, 2876, 1583, 1454, 1327, 1067, 922, 843, 692.

**EI-MS m/z (rel. int.):** 208 (M^+^, 20), 109 (100), 69 (40), 57 (50).

## ((3s,5s,7s)-adamantan-1-yl)(phenyl)sulfane (3ab)

((3s,5s,7s)-adamantan-1-yl)(phenyl)sulfane was obtained as a white solid in 96% yield.

**^1^H NMR:** (400 MHz, CDCl_3_) δ 7.54 – 7.48 (m, 2H), 7.40 – 7.27 (m, 3H), 2.05 – 1.95 (m, 3H), 1.82 (d, *J* = 3.0 Hz, 6H), 1.70 – 1.56 (m, 6H).

**^13^C NMR:** (101 MHz, CDCl_3_) δ 137.8, 130.7, 128.7, 128.4, 48.0, 43.8, 36.3, 30.1.

**IR: (neat)** vmax cm^-1^: 2900, 2847, 1441, 1340, 1295, 1037, 826, 752, 705, 693.

**EI-MS m/z (rel. int.):** 244 (M^+^, 30), 135 (100), 109 (95), 79 (50).

## (1-Methylcyclopentyl)(phenyl)sulfane (3ac)

(1-Methylcyclopentyl)(phenyl)sulfane was obtained as a colorless oil in 81% yield.

**^1^H NMR:** (400 MHz, CDCl_3_) δ 7.49 – 7.42 (m, 2H), 7.29 – 7.19 (m, 3H), 1.84 – 1.70 (m, 4H), 1.67 – 1.53 (m, 2H), 1.50 – 1.40 (m, 2H), 1.27 (s, 3H).

**^13^C NMR:** (101 MHz, CDCl_3_) δ 137.1, 134.1, 128.9, 128.8, 57.0, 40.8, 29.1, 24.6.

**IR: (neat)** vmax cm^-1^: 3072, 2861, 1582, 1437, 1303, 1089, 1024, 917, 747, 692.

**EI-MS m/z (rel. int.):** 192 (M^+^, 10), 110 (100), 65 (50), 55 (45).

## 4-Methyl-4-(phenylthio)pentan-2-one (3ad)

4-Methyl-4-(phenylthio)pentan-2-one was obtained as a colorless oil in 75% yield.

**^1^H NMR:** (400 MHz, CDCl_3_) δ 7.49 – 7.41 (m, 2H), 7.31 – 7.23 (m, 3H), 2.58 (s, 2H), 2.06 (s, 3H), 1.31 (s, 6H).

**^13^C NMR:** (101 MHz, CDCl_3_) δ 206.7, 137.7, 137.0, 131.5, 129.2, 128.7, 128.6, 54.5, 47.1, 32.2, 28.3.

**IR: (neat)** vmax cm^-1^: 2967, 2928, 1708, 1438, 1356, 1118, 1024, 749, 693, 552.

**EI-MS m/z (rel. int.):** 208 (M^+^, 10), 151 (5), 110 (100), 65 (20).

## Cyclohex-2-en-1-yl(phenyl)sulfane (3ae)

Cyclohex-2-en-1-yl(phenyl)sulfane was obtained as a colorless oil in 90% yield.

**^1^H NMR:** (400 MHz, CDCl_3_) δ 7.38 – 7.28 (m, 2H), 7.26 – 7.05 (m, 3H), 5.96 – 5.53 (m, 2H), 3.78 (s, 1H), 2.04 – 1.91 (m, 2H), 1.91 – 1.76 (m, 2H), 1.76 – 1.65 (m, 1H), 1.61 – 1.43 (m, 1H).

**^13^C NMR:** (101 MHz, CDCl_3_) δ 135.7, 131.1, 130.2, 128.6, 126.7, 126.4, 43.7, 28.6, 24.7, 19.2.

**IR: (neat)** vmax cm^-1^: 3056, 2933, 1645, 1479, 1255, 1089, 985, 835, 754, 689.

**EI-MS m/z (rel. int.):** 190 (M^+^, 40%), 109 (80), 81 (100), 65 (50).

## (9H-fluoren-9-yl)(phenyl)sulfane (3af)

(9H-fluoren-9-yl)(phenyl)sulfane was obtained as a solid in 93% yield.

**^1^H NMR:** (400 MHz, CDCl_3_) δ 7.75 – 7.63 (m, 4H), 7.50 – 7.31 (m, 6H), 7.27 – 7.17 (m, 3H), 5.35 (s, 1H).

**^13^C NMR:** (101 MHz, CDCl_3_) δ 144.5, 140.6, 133.3, 128.6, 127.7, 127.4, 125.5, 120.0, 51.8.

**EI-MS m/z (rel. int.):** 274 (M^+^, 15%), 165 (100), 139 (10), 109 (10).

## Benzhydryl(phenyl)sulfane (3ag)

Benzhydryl(phenyl)sulfane was obtained as a white solid in 99% yield.

**^1^H NMR:** (400 MHz, CDCl_3_) δ 7.49 – 7.37 (m, 4H), 7.35 – 7.28 (m, 4H), 7.28 – 7.23 (m, 4H), 7.23 – 7.13 (m, 3H), 5.56 (s, 1H).

**^13^C NMR:** (101 MHz, CDCl_3_) δ 141.4, 136.6, 130.9, 129.2, 129.0, 128.8, 127.7, 127.0, 57.8.

**IR: (neat)** vmax cm^-1^: 3025, 1581, 1489, 1479, 1024, 786, 694, 626, 614, 588.

**EI-MS m/z (rel. int.):** 276 (M^+^, 5%), 167 (100), 152 (15), 108 (5).

## Phenyl(1-phenylpropyl)sulfane (3ah)

Phenyl(1-phenylpropyl)sulfane was obtained as a colorless oil in 91% yield.

**^1^H NMR:** (400 MHz, CDCl_3_) δ 7.34 – 7.27 (m, 6H), 7.26 – 7.18 (m, 4H), 4.10 (dd, *J* = 8.8, 6.1 Hz, 1H), 2.21 – 1.78 (m, 2H), 0.96 (t, *J* = 7.3 Hz, 3H).

**^13^C NMR:** (101 MHz, CDCl_3_) δ 142.0, 135.3, 132.4, 128.7, 128.4, 128.0, 127.1, 127.0, 55.4, 29.5, 12.4.

**IR: (neat)** vmax cm^-1^: 3059, 2963, 2872, 1583, 1479, 1451, 1438, 1377, 1025, 689.

**EI-MS m/z (rel. int.):** 228 (M^+^, 15%), 197 (5), 119 (50), 91 (100).

## Phenyl(1-phenylethyl)sulfane (3ai)

Phenyl(1-phenylethyl)sulfane was obtained as a colorless oil in 92% yield.

**^1^H NMR:** (400 MHz, CDCl_3_) δ 7.45 – 7.20 (m, 10H), 4.42 (q, *J* = 7.0 Hz, 1H), 1.71 (d, *J* = 7.1 Hz, 3H).

**^13^C NMR:** (101 MHz, CDCl_3_) δ 143.6, 135.6, 132.9, 129.1, 128.8, 127.7, 127.5, 127.5, 48.4, 22.7.

**IR: (neat)** vmax cm^-1^: 3060, 2970, 1874, 1804, 1583, 1449, 1221, 840, 696.

**EI-MS m/z (rel. int.):** 214 (M^+^, 15%), 109 (45), 105 (100), 77 (40).

## Phenyl(1-(p-tolyl)ethyl)sulfane (3aj)

Phenyl(1-(p-tolyl)ethyl)sulfane was obtained as a yellow oil in 88% yield.

**^1^H NMR:** (400 MHz, CDCl_3_) δ 7.43 – 7.33 (m, 2H), 7.30 – 7.22 (m, 4H), 7.17 – 7.09 (m, 3H), 4.39 (q, *J* = 7.0 Hz, 1H), 2.36 (s, 3H), 1.67 (d, *J* = 7.0 Hz, 3H).

**^13^C NMR:** (101 MHz, CDCl_3_) δ 140.6, 137.2, 135.8, 132.7, 129.5, 129.1, 127.6, 127.4, 48.0, 22.9, 21.5.

**IR: (neat)** vmax cm^-1^: 2966, 2921, 1583, 1512, 1479, 1438, 1371, 816, 737, 690.

**EI-MS m/z (rel. int.):** 228 (M^+^, 10%), 119 (100), 109 (10), 91 (15).

## (1-(4-Methoxyphenyl)ethyl)(phenyl)sulfane (3ak)

(1-(4-Methoxyphenyl)ethyl)(phenyl)sulfane was obtained as a yellow oil in 92% yield.

**^1^H NMR:** (400 MHz, CDCl_3_) δ 7.37 – 7.31 (m, 2H), 7.30 – 7.19 (m, 5H), 6.90 – 6.74 (m, 2H), 4.37 (q, *J* = 7.0 Hz, 1H), 3.80 (s, 3H), 1.64 (d, *J* = 7.0 Hz, 3H).

**^13^C NMR:** (101 MHz, CDCl_3_) δ 158.7, 135.3, 132.5, 128.8, 128.7, 128.4, 127.1, 113.8, 55.3, 55.3, 47.4, 22.5.

**IR: (neat)** vmax cm^-1^: 2962, 2926, 2833, 1609, 1583, 1509, 1438, 1243, 1175, 829.

**EI-MS m/z (rel. int.):** 244 (M^+^, 5%), 135 (100), 119 (10), 91 (35).

## (1-(4-Fluorophenyl)ethyl)(phenyl)sulfane (3al)

(1-(4-Fluorophenyl)ethyl)(phenyl)sulfane was obtained as an orange oil in 83% yield.

**^1^H NMR:** (400 MHz, CDCl_3_) δ 7.36 – 7.13 (m, 7H), 6.96 – 6.84 (m, 2H), 4.74 – 3.99 (m, 1H), 1.93 – 1.37 (m, 3H).

**^13^C NMR:** (101 MHz, CDCl_3_) δ 161.89 (d, *J* = 245.4 Hz), 139.10 (d, *J* = 3.0 Hz), 134.86 (d, *J* = 2.5 Hz), 132.8, 128.9, 128.8, 127.4, 115.25 (d, *J* = 21.4 Hz), 47.4, 22.5.

**^19^F NMR:** (377 MHz, CDCl_3_) δ -115.4.

**IR: (neat)** vmax cm^-1^: 3058, 2969, 2924, 1603, 1584, 1507, 1221, 1157, 833, 690.

**EI-MS m/z (rel. int.):** 232 (M^+^, 5%), 123 (100), 109 (35), 96 (10).

## (1-(4-Chlorophenyl)ethyl)(phenyl)sulfane (3am)

(1-(4-Chlorophenyl)ethyl)(phenyl)sulfane was obtained as a colorless oil in 93% yield.

**^1^H NMR:** (400 MHz, CDCl_3_) δ 7.35 – 7.29 (m, 2H), 7.29 – 7.18 (m, 7H), 4.33 (q, *J* = 7.0 Hz, 1H), 1.63 (d, *J* = 7.1 Hz, 3H).

**^13^C NMR:** (101 MHz, CDCl_3_) δ 141.8, 134.5, 132.6, 132.6, 128.7, 128.5, 128.4, 127.3, 47.3, 22.1.

**IR: (neat)** vmax cm^-1^: 3057, 2968, 2923, 1583, 1490, 1438, 1406, 1091, 1013, 827.

**EI-MS m/z (rel. int.):** 248 (M^+^, 10%), 139 (100), 103 (50), 77 (30).

## Phenyl((1S,2R,4S)-1,7,7-trimethylbicyclo[2.2.1]heptan-2-yl)sulfane (3an)

Phenyl((1S,2R,4S)-1,7,7-trimethylbicyclo[2.2.1]heptan-2-yl)sulfane was obtained as a colorless oil in 64% yield.

**^1^H NMR:** (300 MHz, CDCl_3_) δ 7.59 – 7.10 (m, 5H), 3.28 (t, *J* = 7.5 Hz, 1H), 2.09 – 2.02 (m, 2H), 1.85 – 1.73 (m, 3H), 1.36 – 1.20 (m, 2H), 1.10 (s, 3H), 1.07 (s, 3H), 0.91 (s, 3H).

**^13^C NMR:** (75 MHz, CDCl_3_) δ 129.4, 129.2, 128.8, 127.6, 127.2, 125.6, 56.3, 50.0, 47.7, 46.0, 41.1, 38.7, 27.5, 20.7, 20.3, 14.1.

**IR: (neat)** vmax cm^-1^: 2949, 1582, 1477, 1388, 1088, 1024, 907, 723, 688.

**EI-MS m/z (rel. int.):** 247 (M^+^, 10%), 109 (100).

## Benzyl(phenyl)sulfane (3ao)

Benzyl(phenyl)sulfane was obtained as a colorless oil in 89% yield.

**^1^H NMR:** (400 MHz, CDCl_3_) δ 7.37 – 7.25 (m, 8H), 7.25 – 7.18 (m, 2H), 4.15 (s, 2H).

**^13^C NMR:** (101 MHz, CDCl_3_) δ 137.6, 136.5, 129.9, 128.9, 128.9, 128.6, 127.3, 126.4, 39.1.

**IR: (neat)** vmax cm^-1^: 2830, 1227, 1221, 1089, 920, 910, 735.

**EI-MS m/z (rel. int.):** 214 (M^+^, 45%), 164 (5), 109 (30), 91 (100).

## Cinnamyl(phenyl)sulfane (3ap)

Cinnamyl(phenyl)sulfane was obtained as a beige solid in 84% yield.

**^1^H NMR:** (400 MHz, CDCl_3_) δ 7.43 – 7.37 (m, 2H), 7.35 – 7.16 (m, 8H), 6.58 – 6.39 (m, 1H), 6.35 – 6.14 (m, 1H), 3.72 (dd, *J* = 7.1, 1.2 Hz, 2H).

**^13^C NMR:** (101 MHz, CDCl_3_) δ 137.2, 136.3, 133.2, 130.7, 129.3, 129.0, 128.0, 126.9, 126.8, 125.5, 37.6.

**IR: (neat)** vmax cm^-1^: 3054, 3030, 1580, 1488, 1435, 1304, 1215, 1088, 1022, 688.

**EI-MS m/z (rel. int.):** 226 (M^+^, 10%), 197 (5), 117 (100), 91 (5).

## (Cyclopropyl(phenyl)methyl)(phenyl)sulfane (3z)

(Cyclopropyl(phenyl)methyl)(phenyl)sulfane was obtained as a colorless oil in 89% yield.

**^1^H NMR:** (400 MHz, CDCl_3_) δ 7.15 – 7.04 (m, 6H), 7.06 – 6.94 (m, 4H), 3.33 (d, *J* = 9.5 Hz, 1H), 1.24 – 1.03 (m, 1H), 0.57 – 0.29 (m, 2H), 0.21 – 0.00 (m, 2H).

**^13^C NMR:** (101 MHz, CDCl_3_) δ 142.3, 135.0, 133.1, 128.6, 128.3, 128.0, 127.2, 127.1, 59.1, 17.3, 6.6, 5.2.

**IR: (neat)** vmax cm^-1^: 3059, 3002, 1582, 1489, 1451, 1438, 1023, 953, 840, 742, 689.

**EI-MS m/z (rel. int.):** 240 (M^+^, 5%), 210 (5), 131 (100), 109 (40).

# SPECTRA FOR ALL PRODUCTS

## Tert-pentyl(phenyl)sulfane (3a)


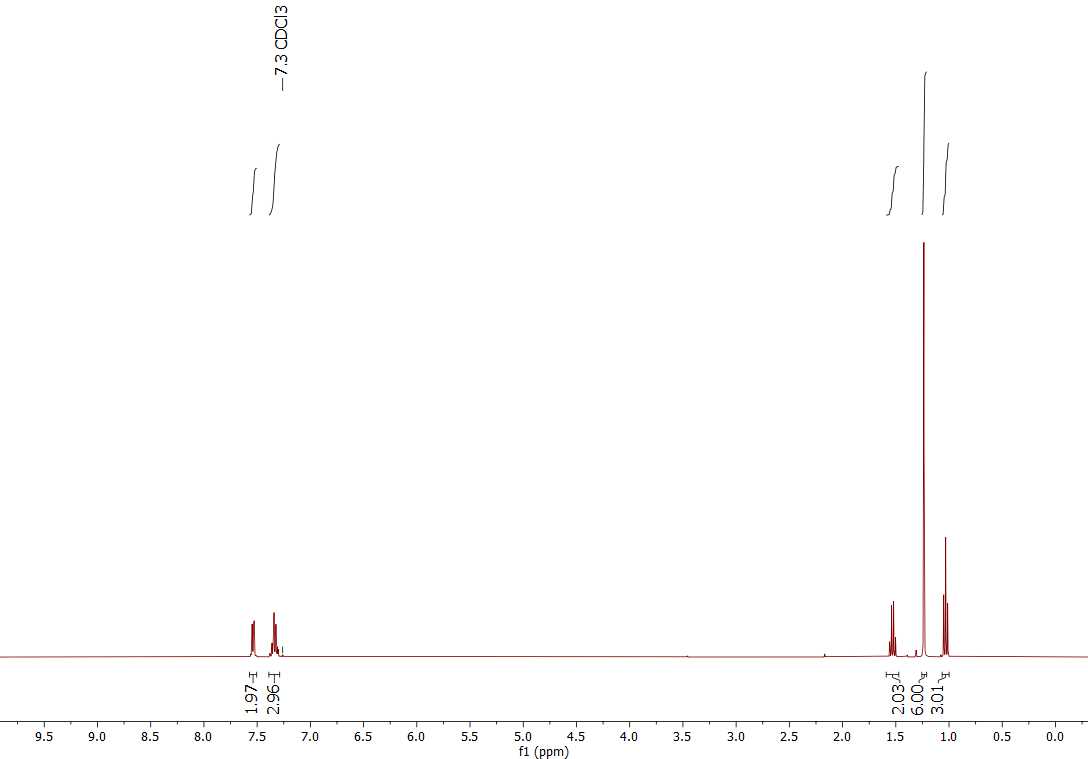


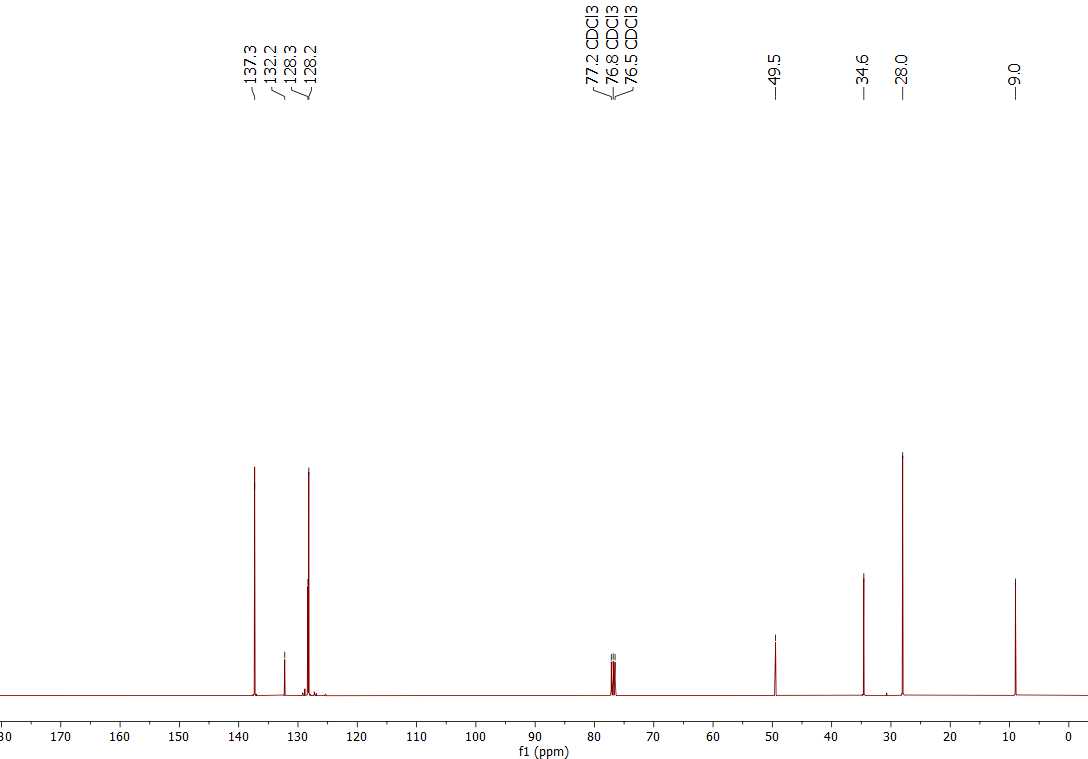


## (3-Ethoxyphenyl)(tert-pentyl)sulfane (3b)

**
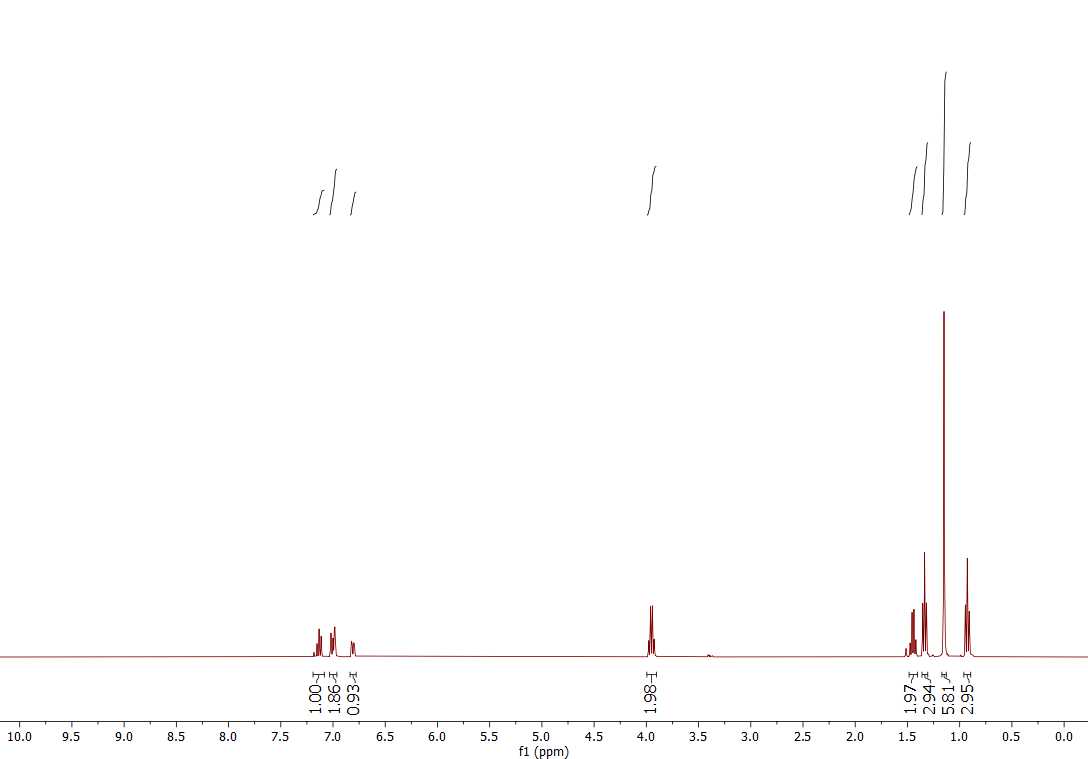
**


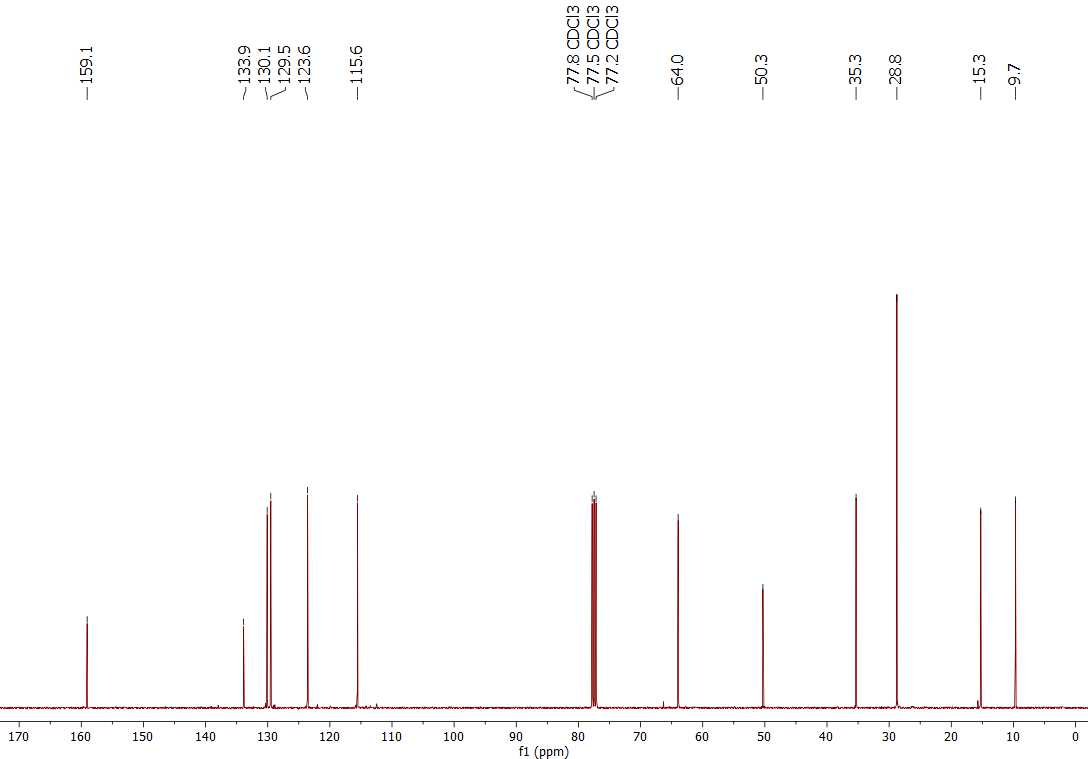


## (2,6-Dimethylphenyl)(tert-pentyl)sulfane (3c)


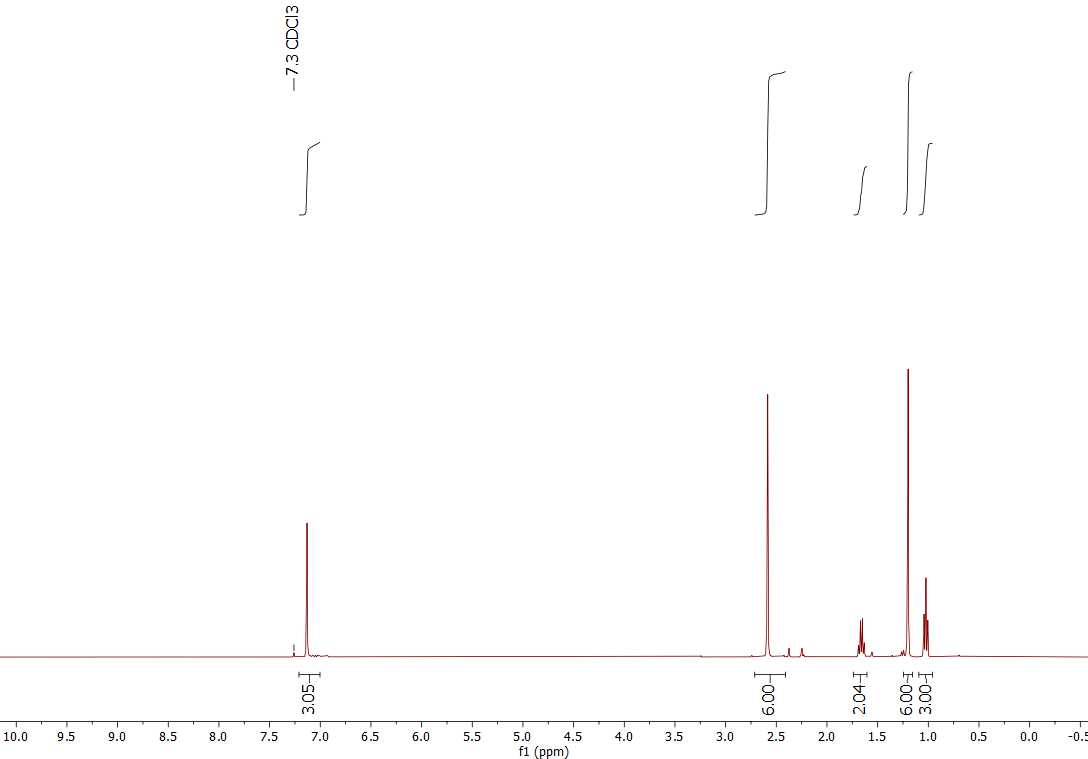

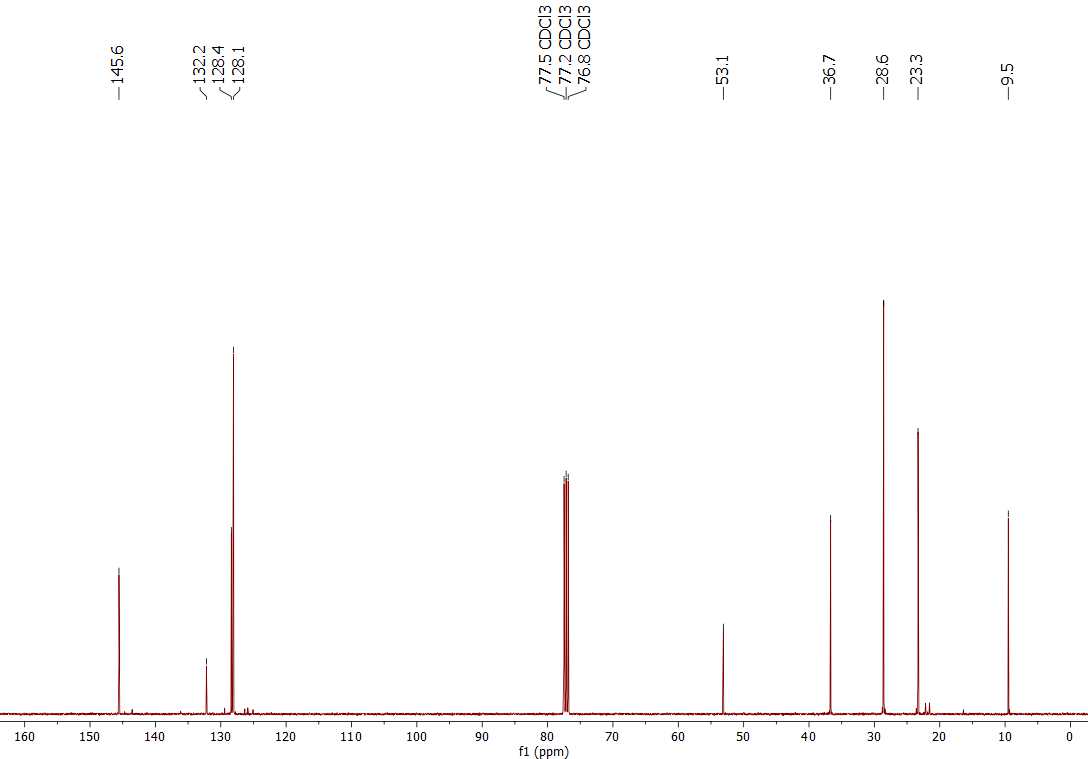


## 4-(tert-Pentylthio)phenol (3d)


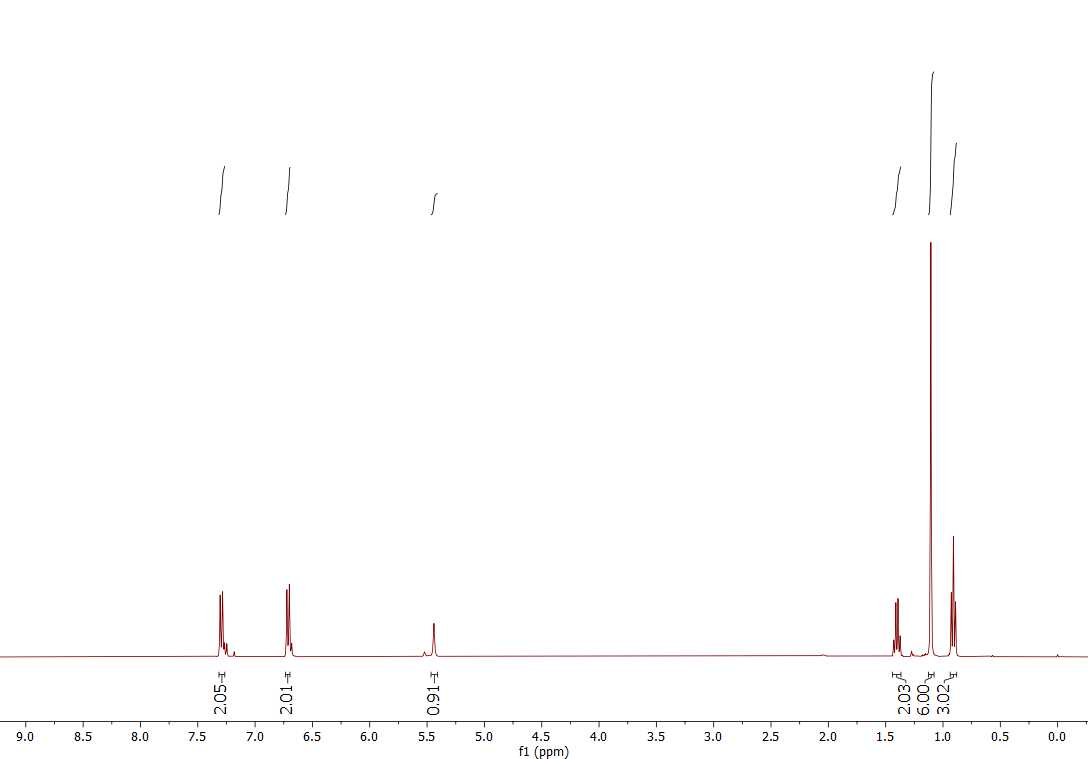


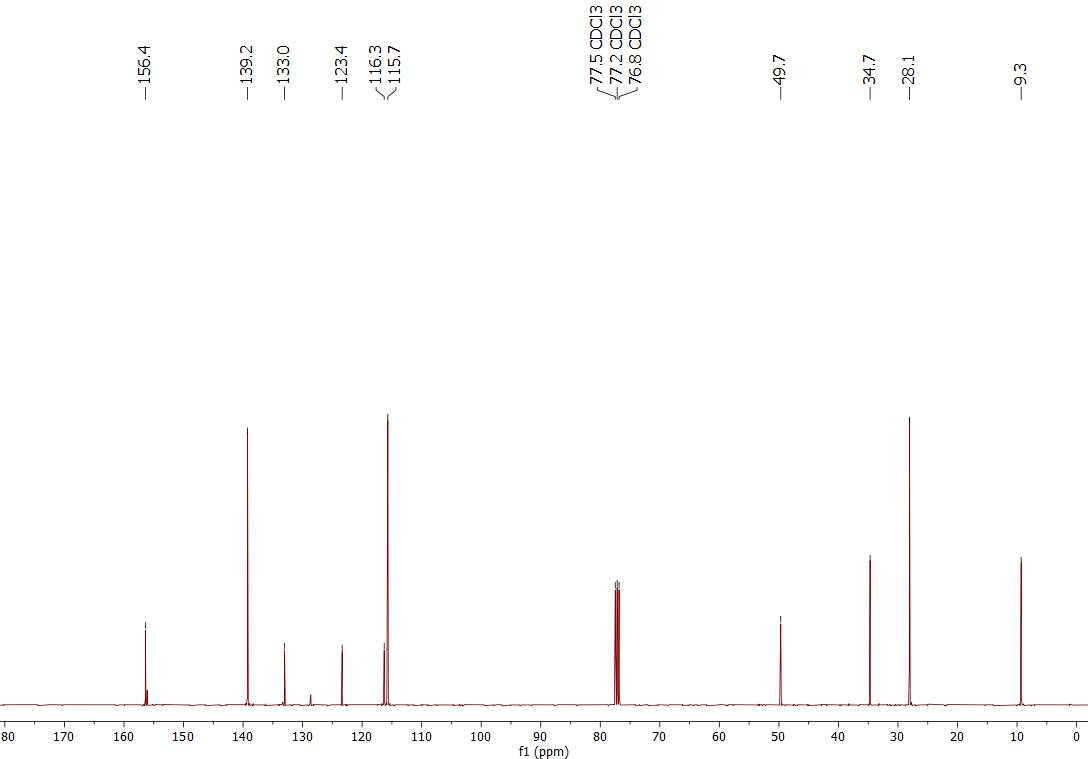


## Naphthalen-2-yl(tert-pentyl)sulfane (3e)


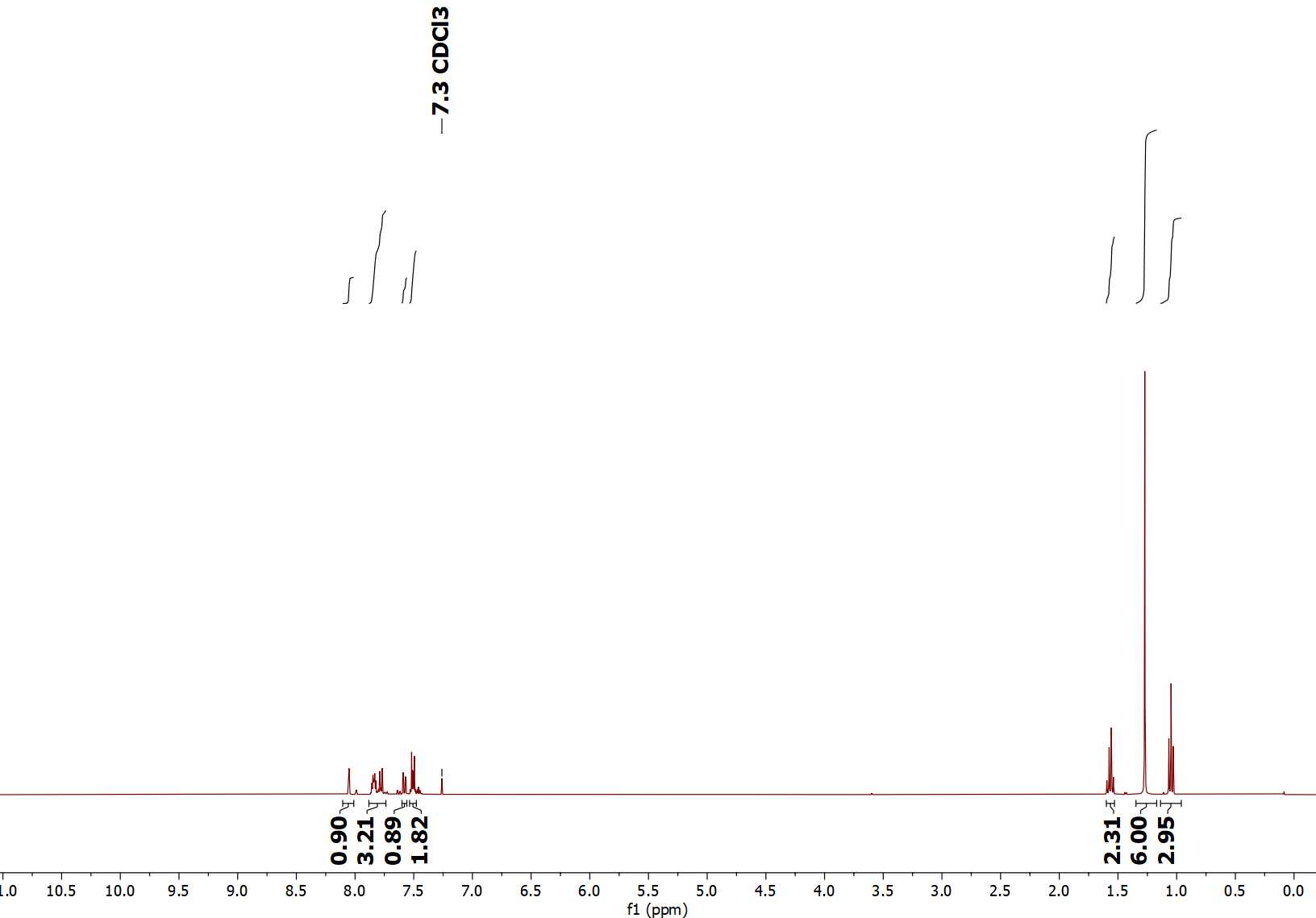


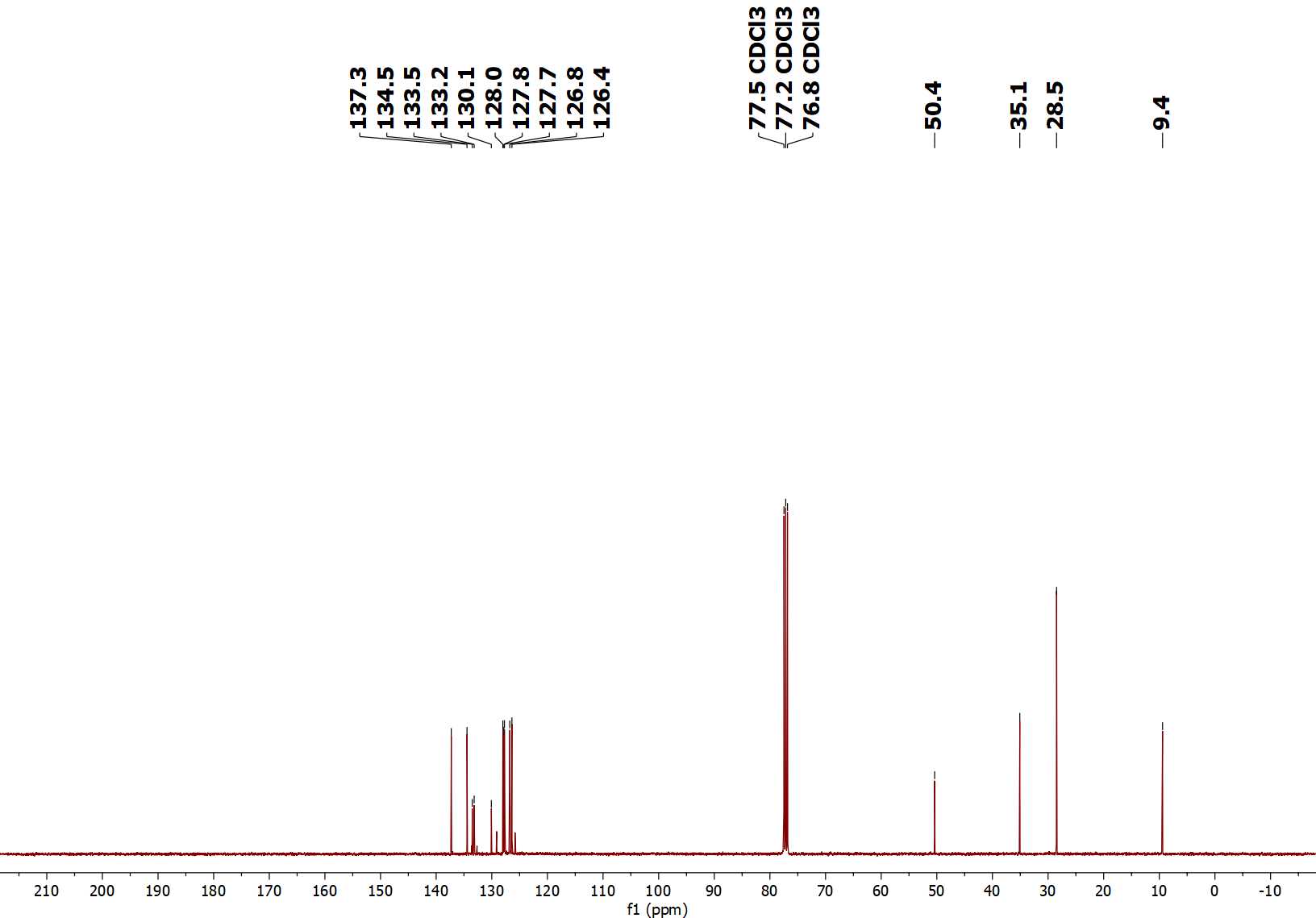


## (4-Chlorophenyl)(tert-pentyl)sulfane (3f)


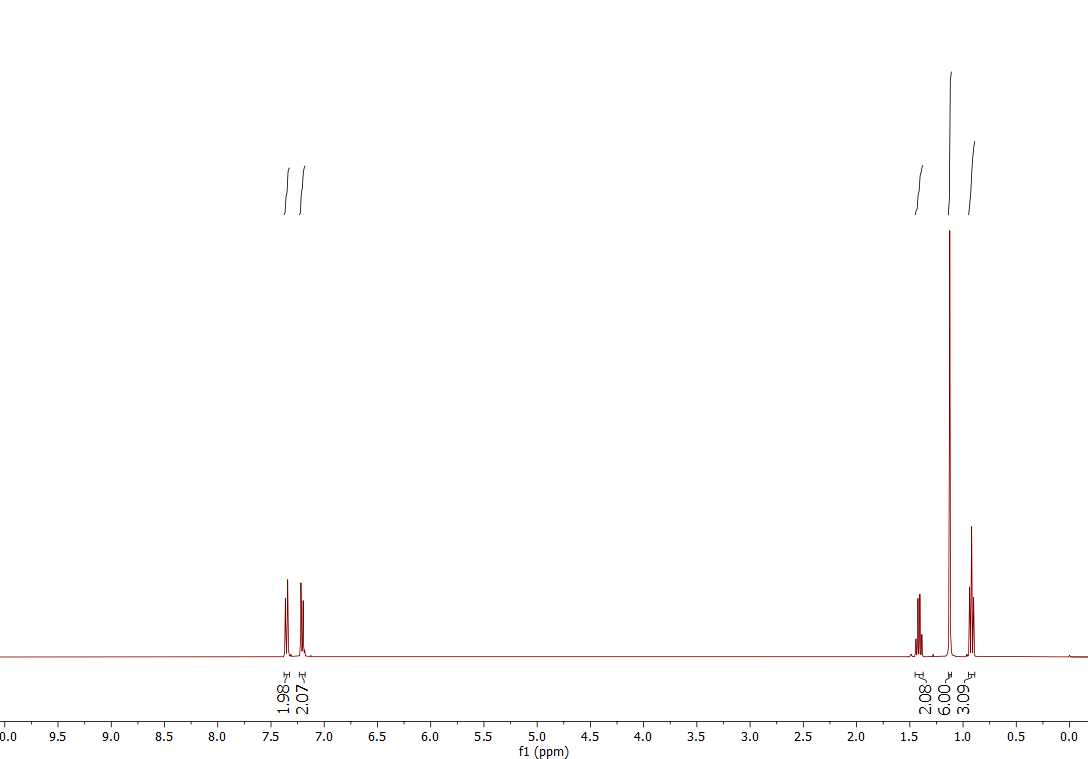

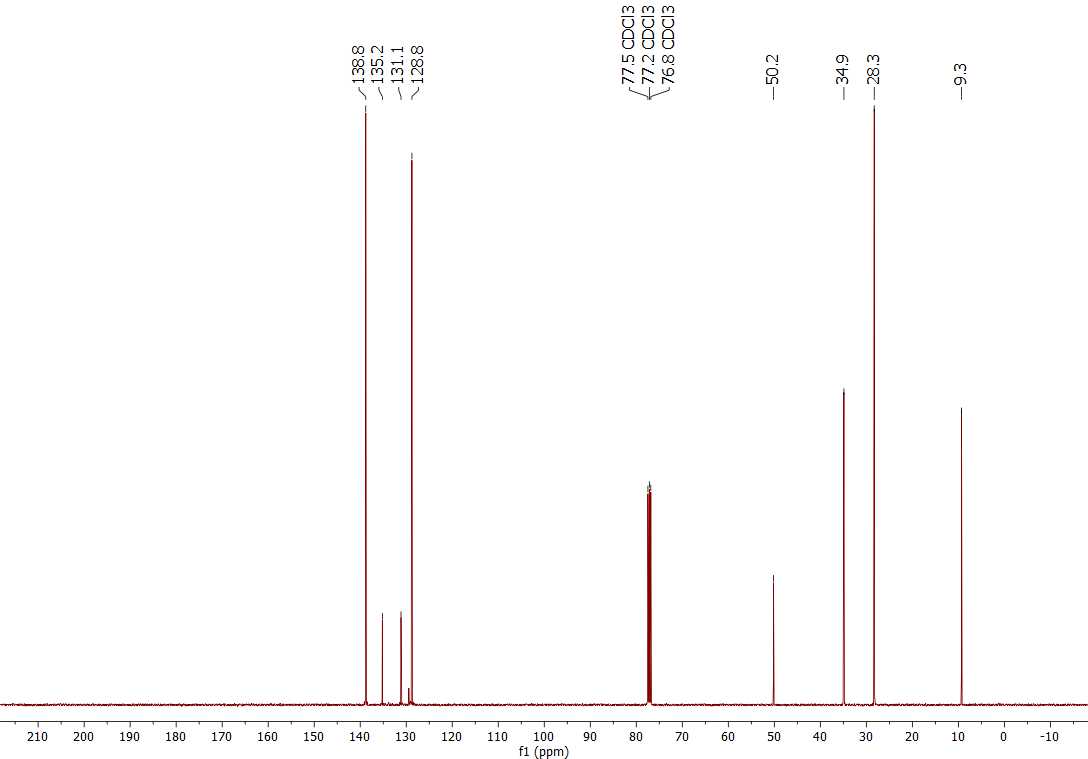


## (3-Chloro-4-fluorophenyl)(tert-pentyl)sulfane (3g)


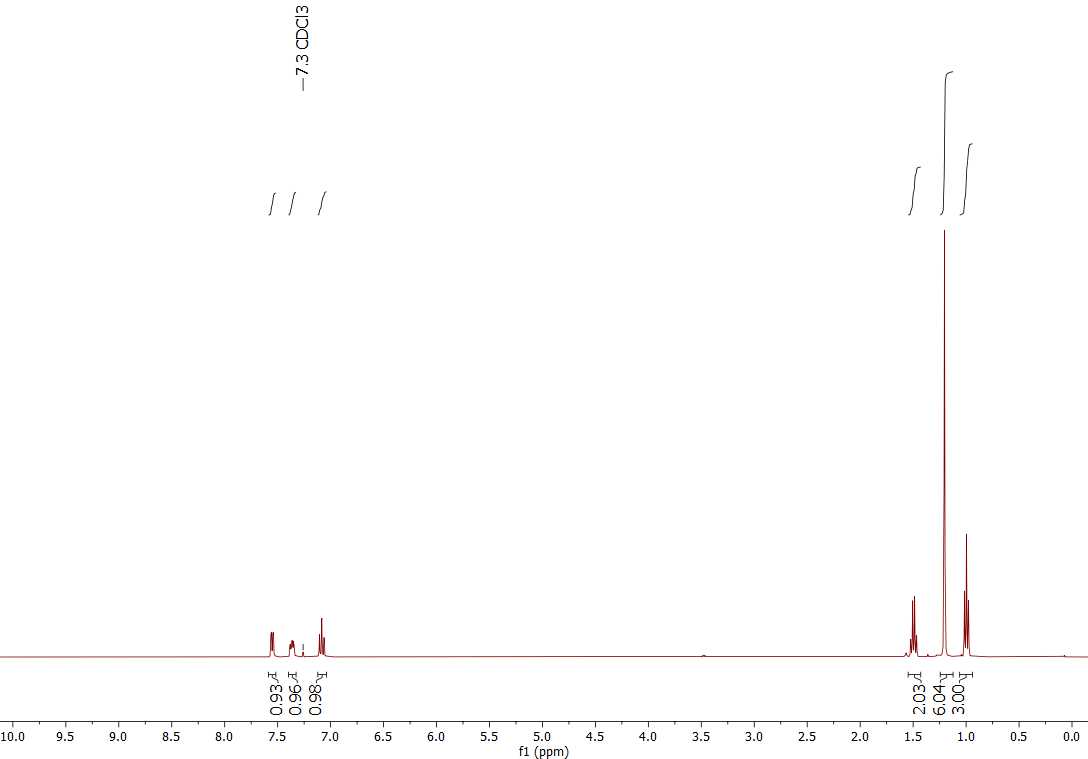


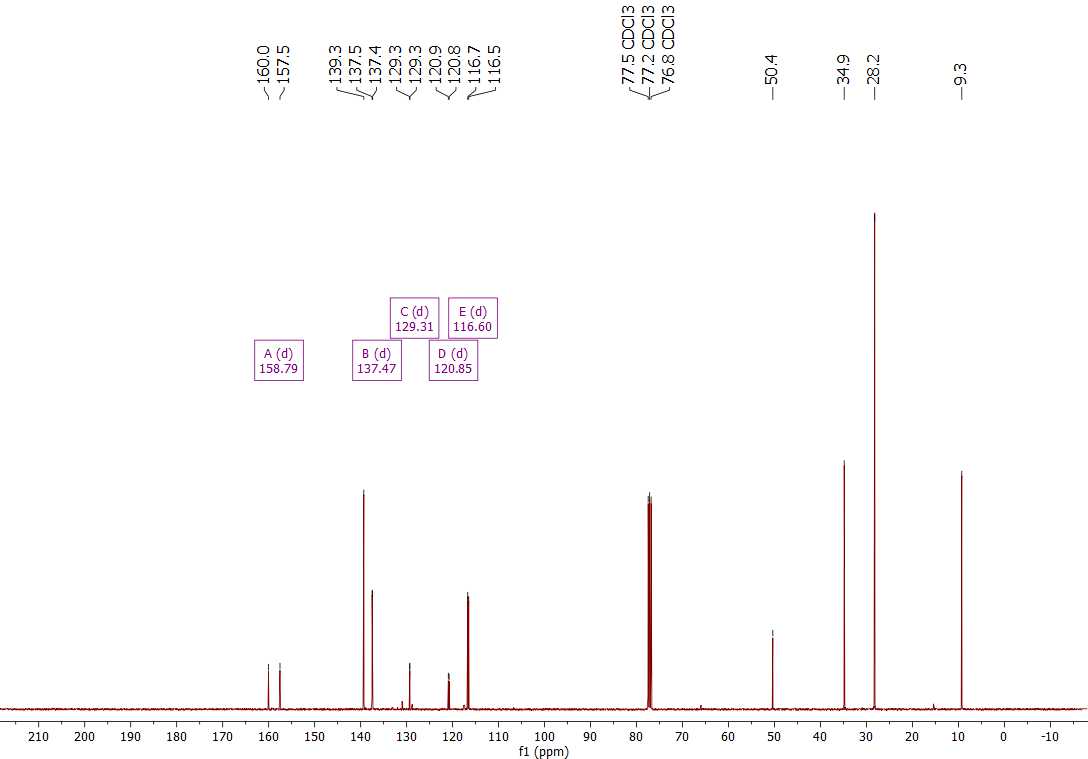


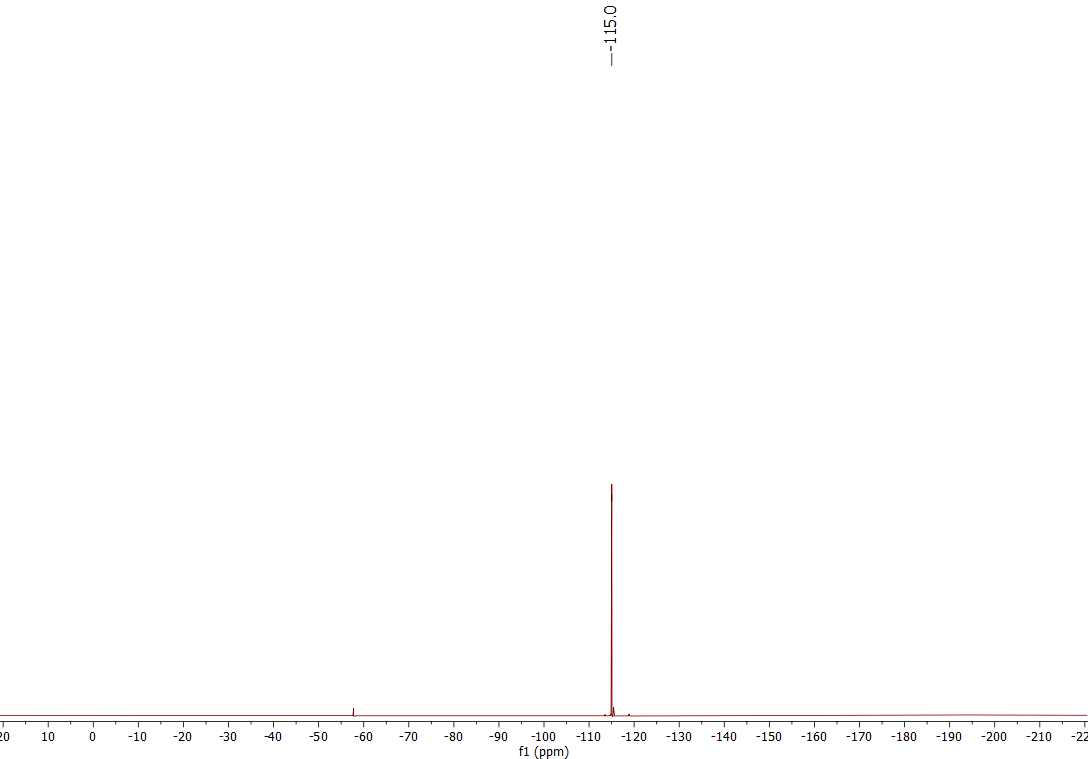


## (4-Bromophenyl)(tert-pentyl)sulfane (3h)


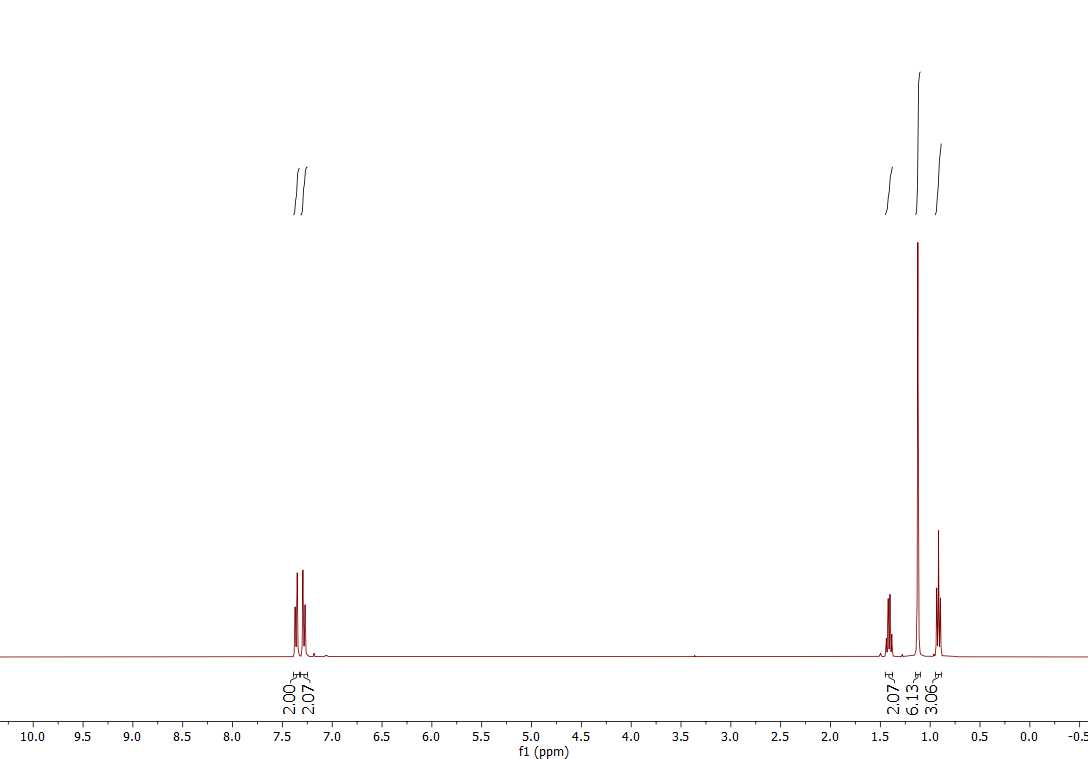


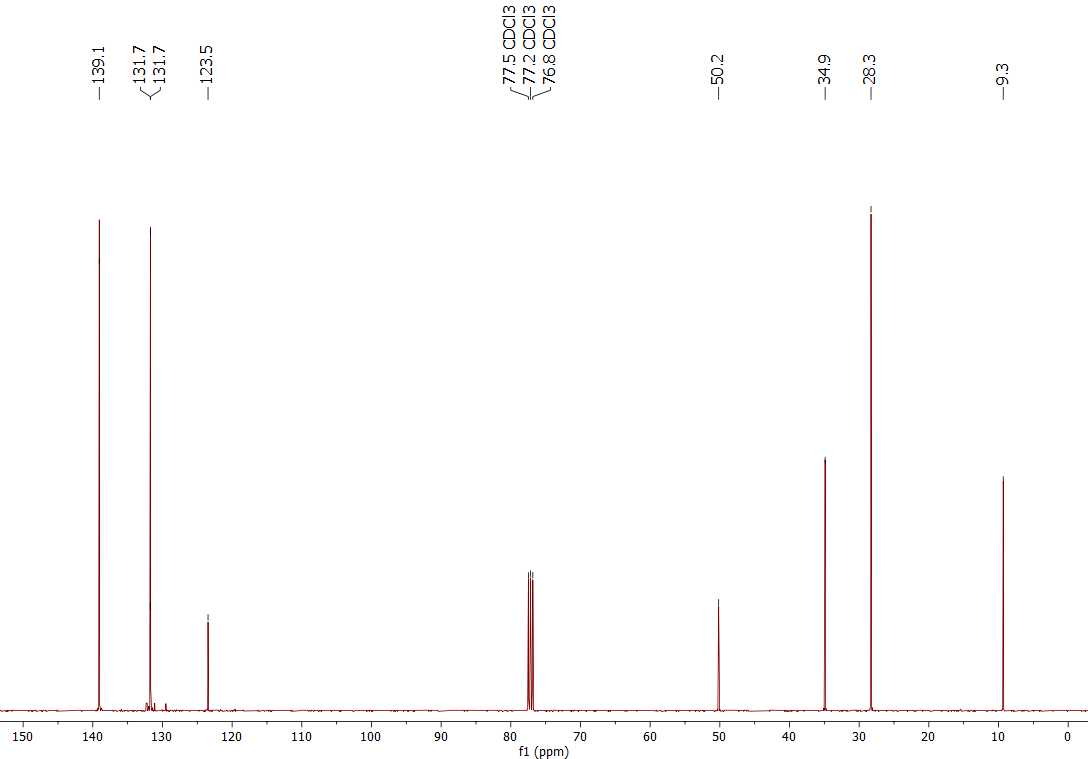


## tert-Pentyl(4-(trifluoromethoxy)phenyl)sulfane (3i)


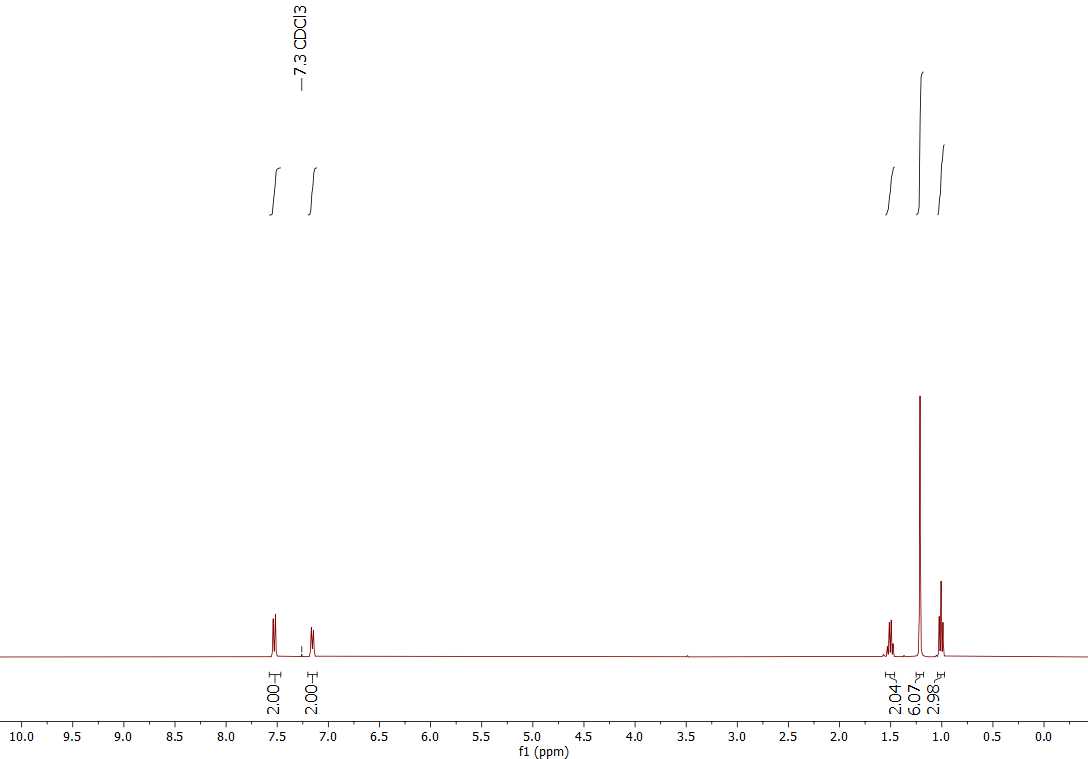


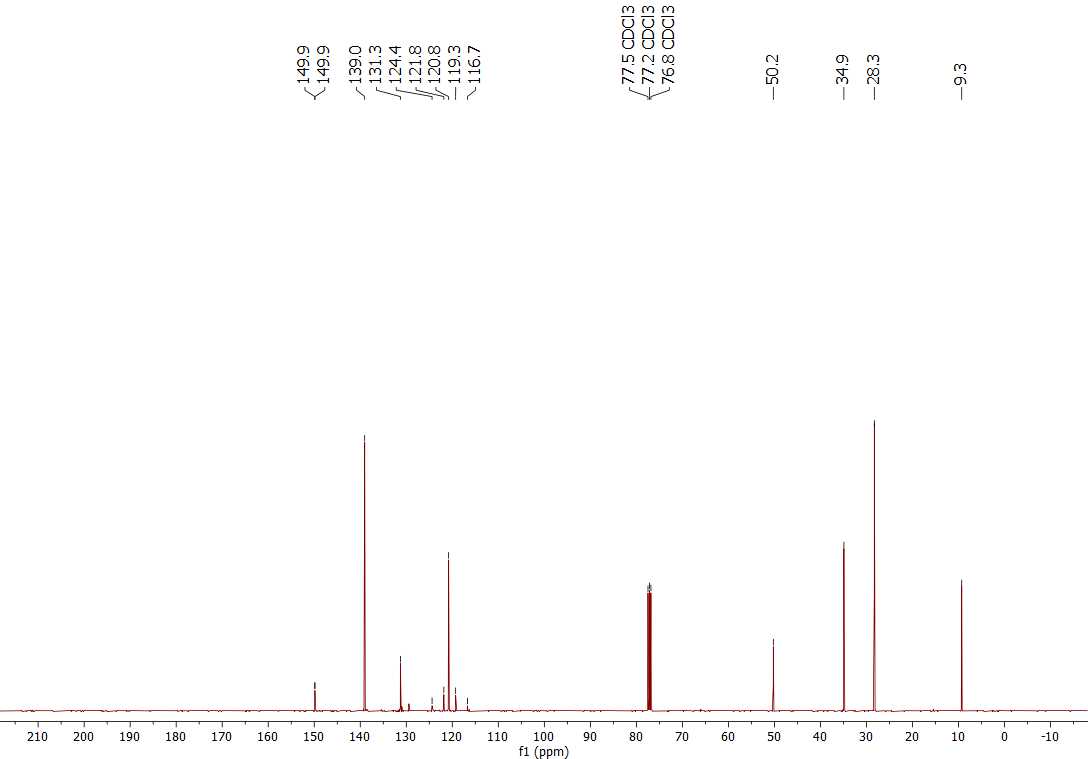


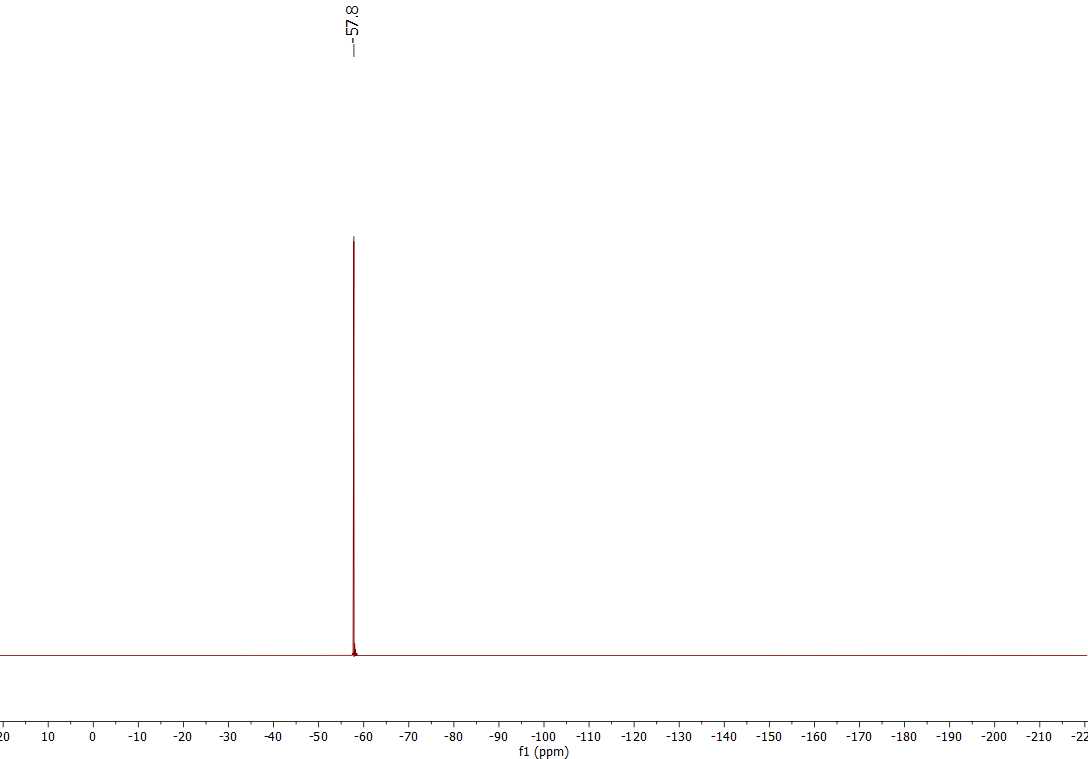


## tert-Pentyl(2-(trifluoromethyl)phenyl)sulfane (3j)


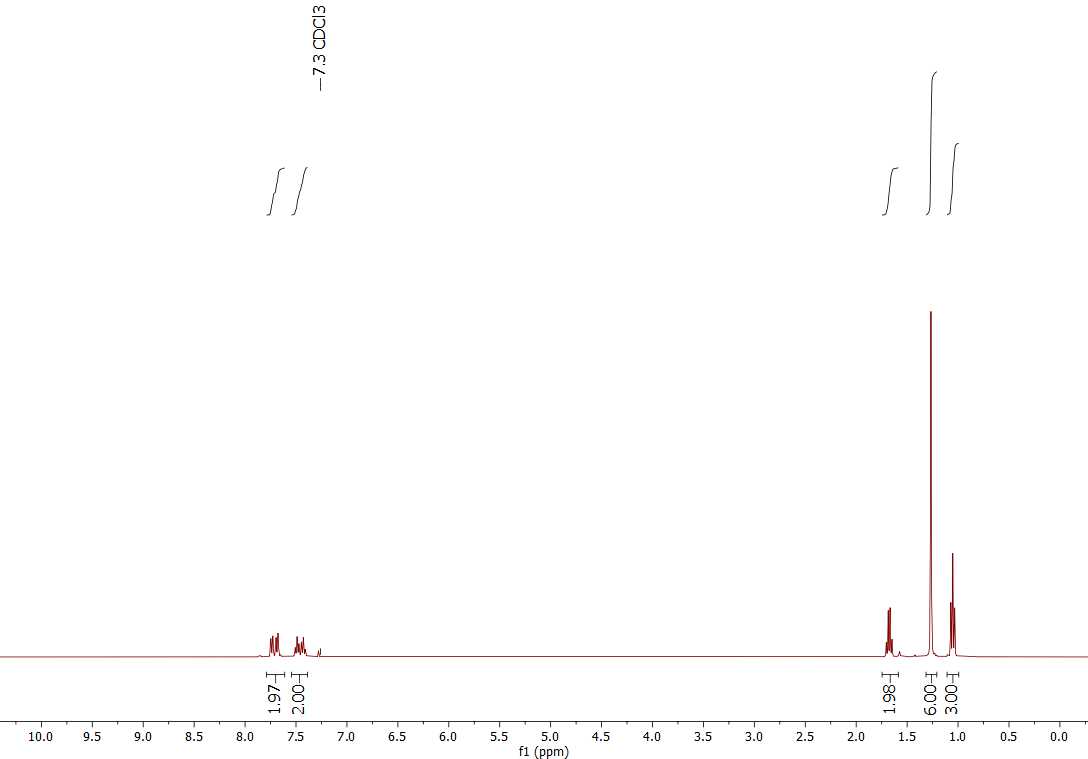


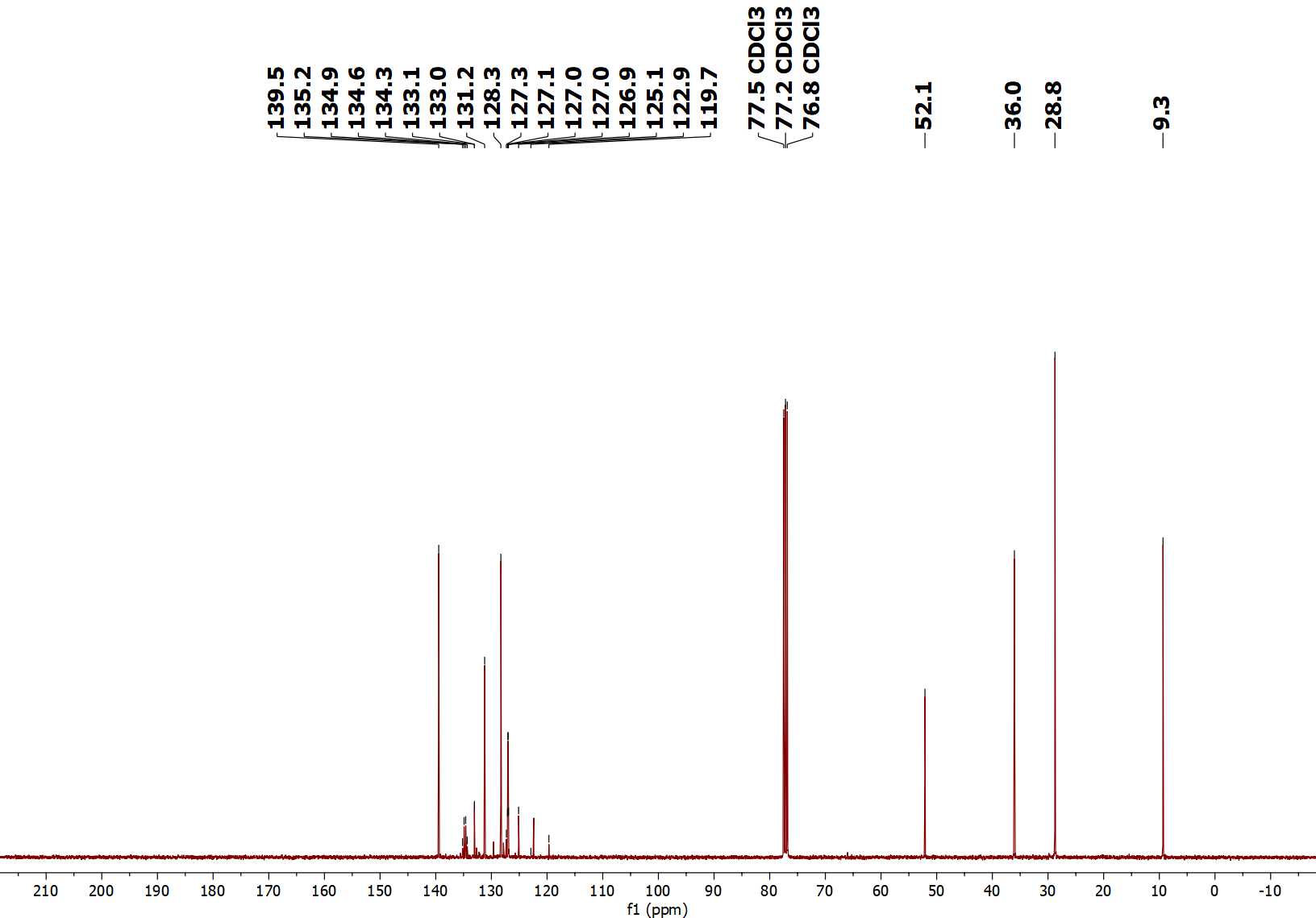


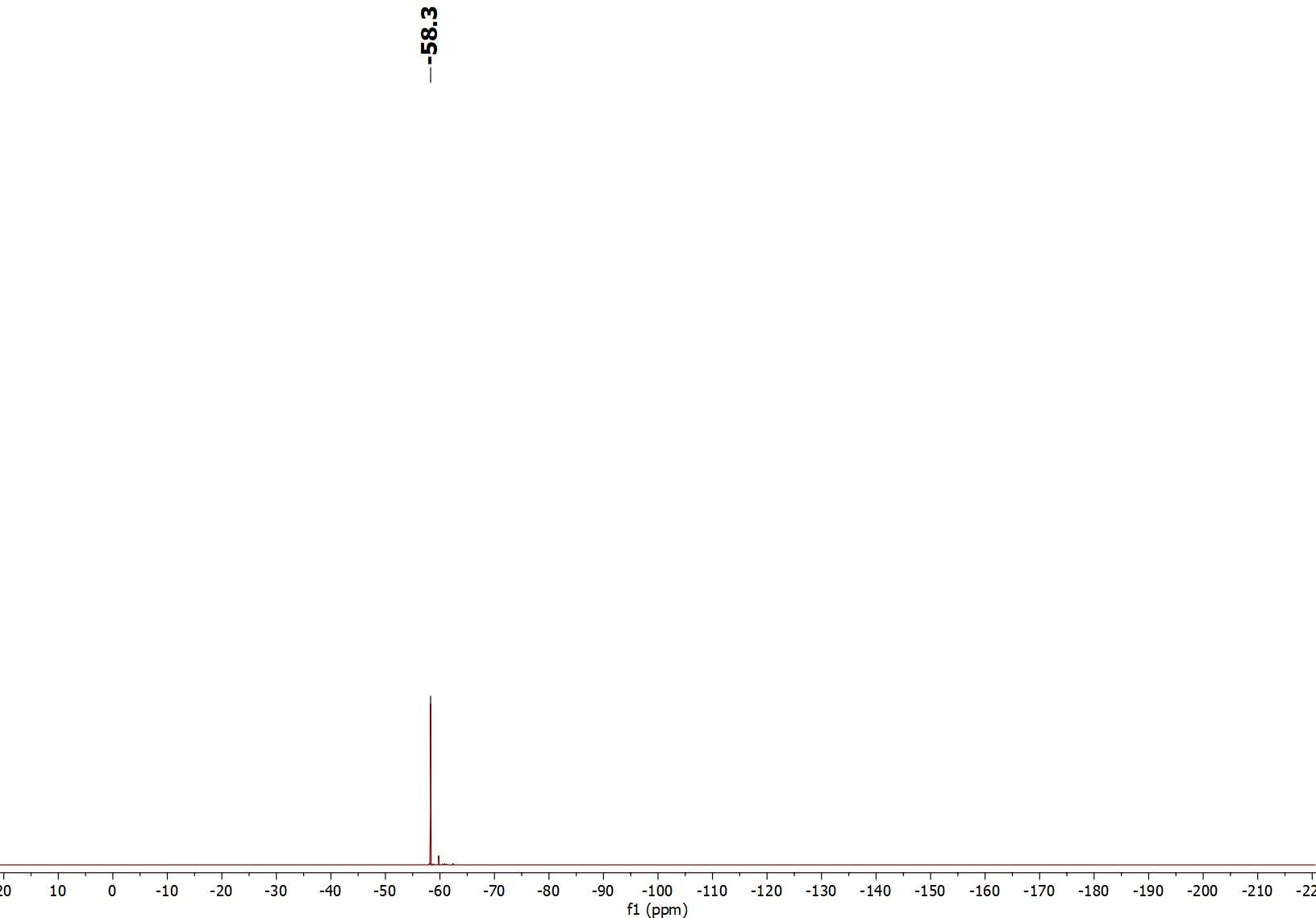


## 2-(tert-Pentylthio)thiophene (3k)


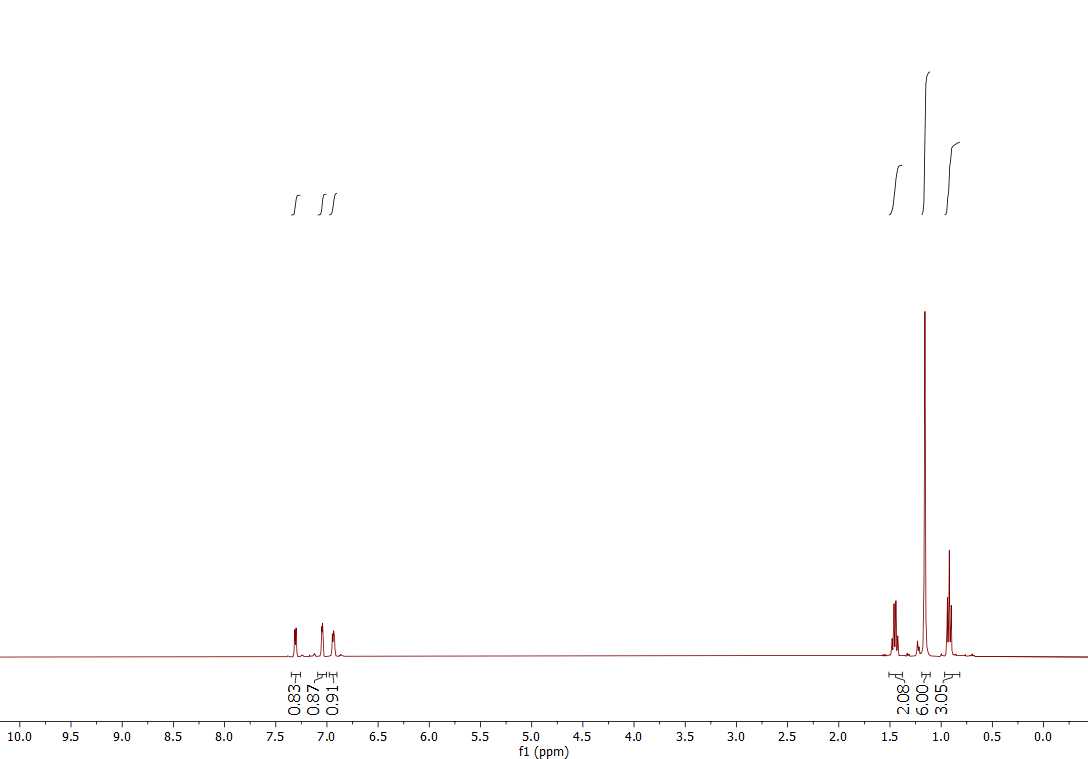


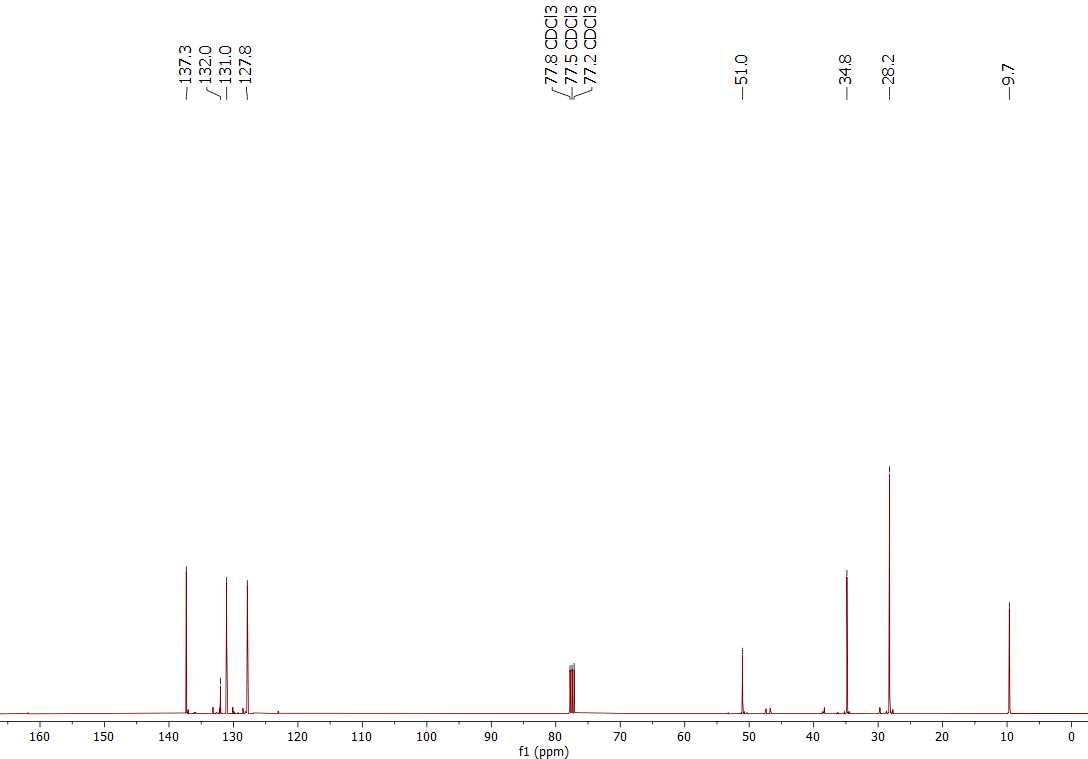


## 2-(tert-Pentylthio)thiophene (3l)


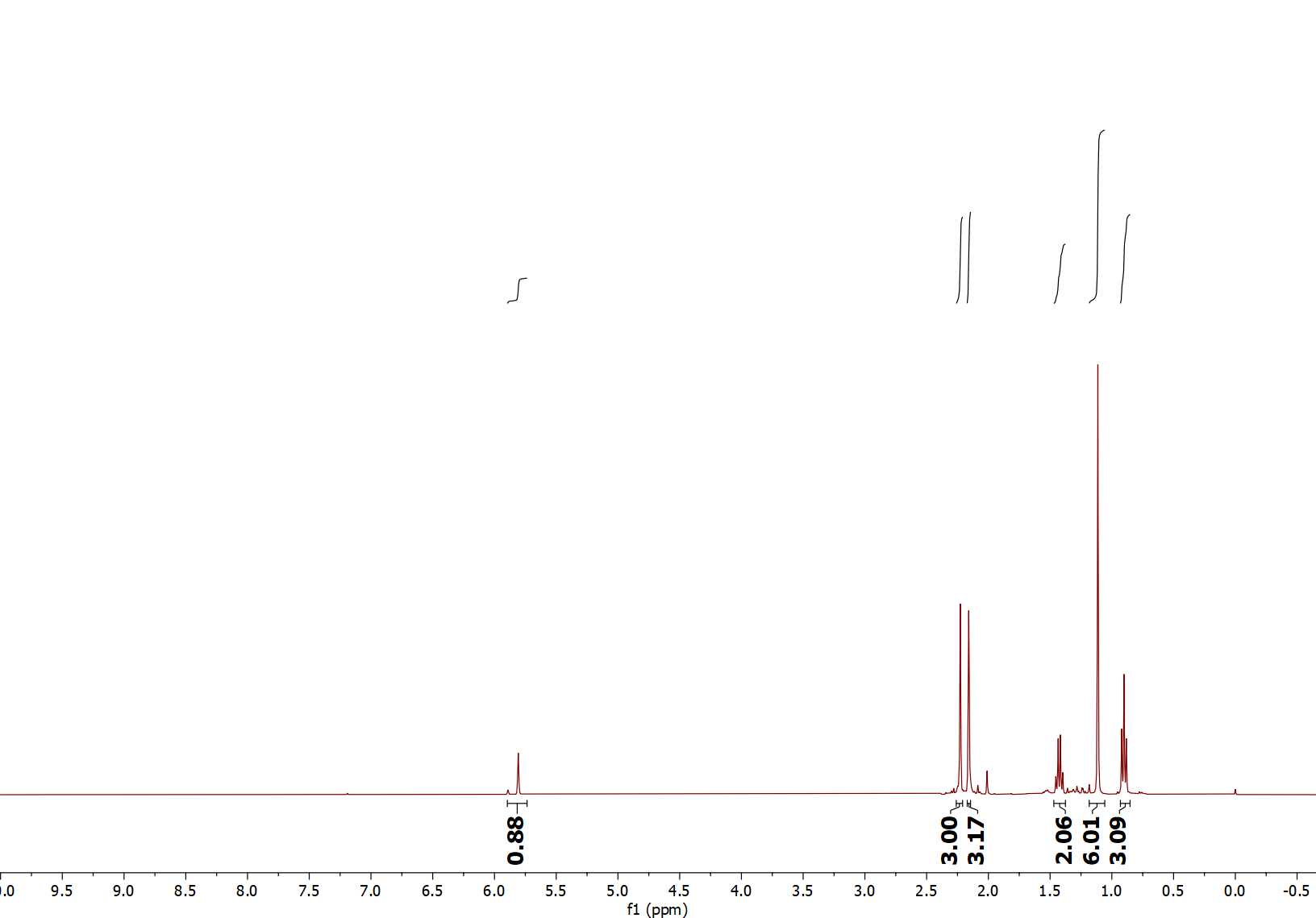


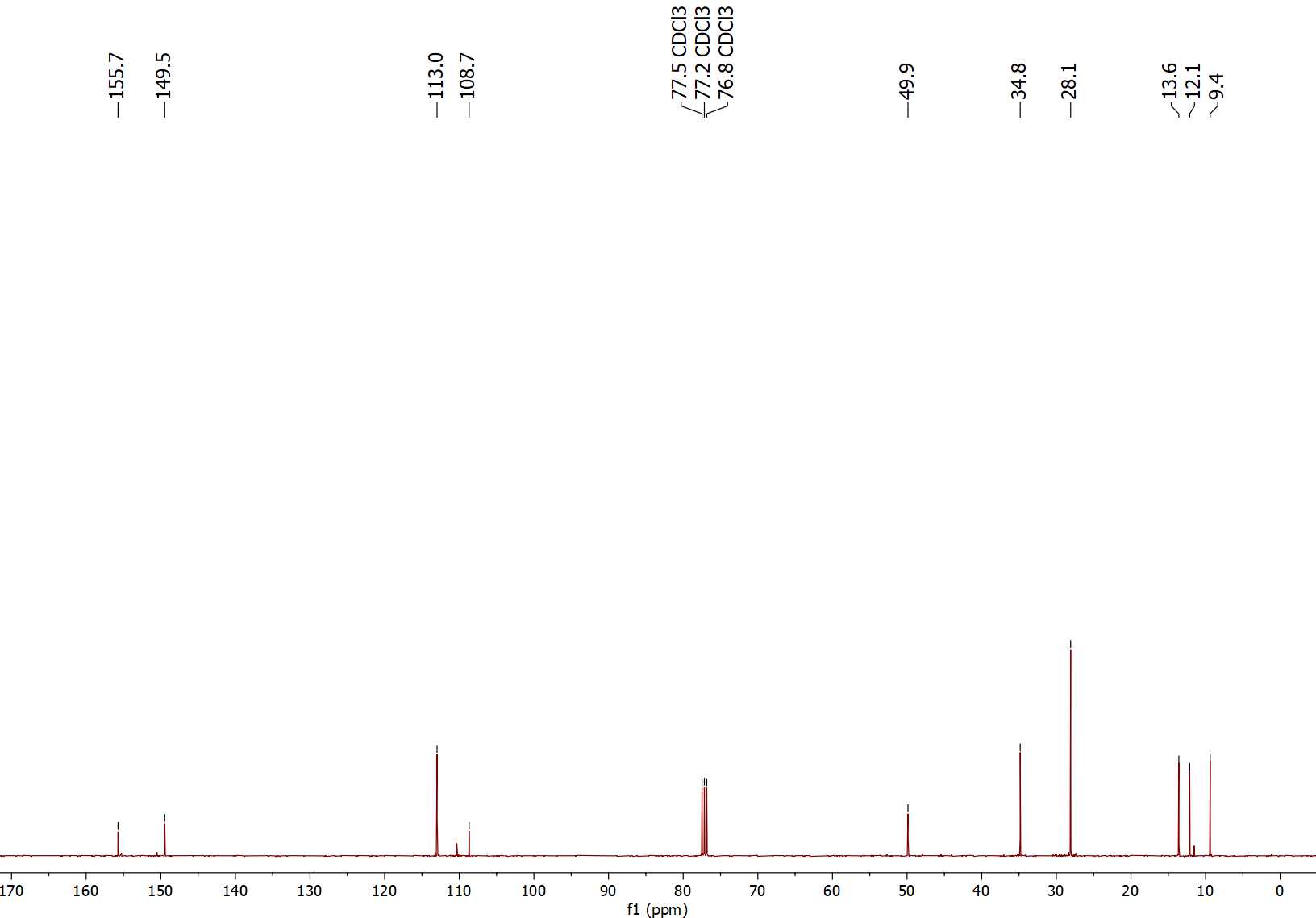


## 3-(tert-Pentylthio)propanoic acid (3m)


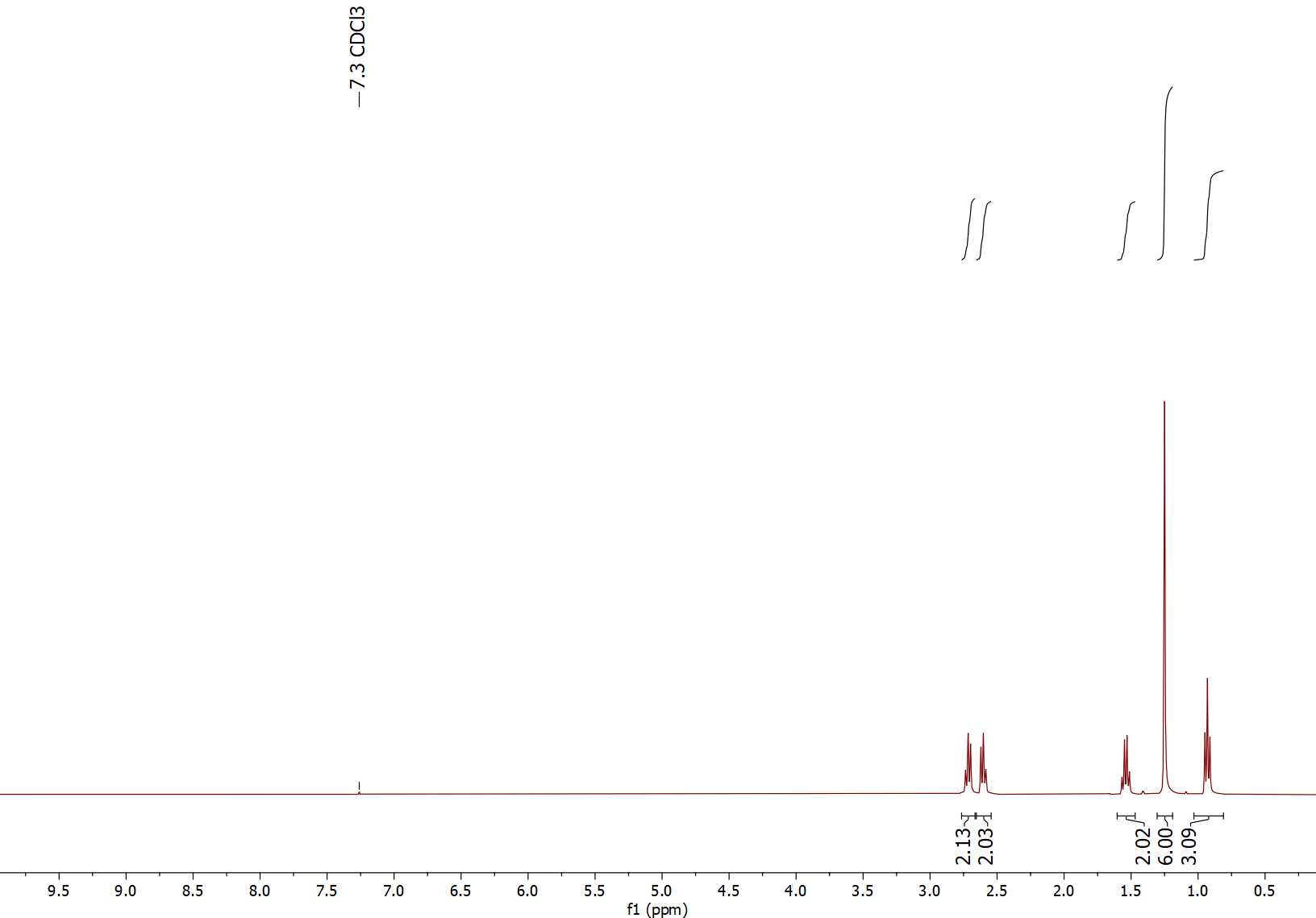


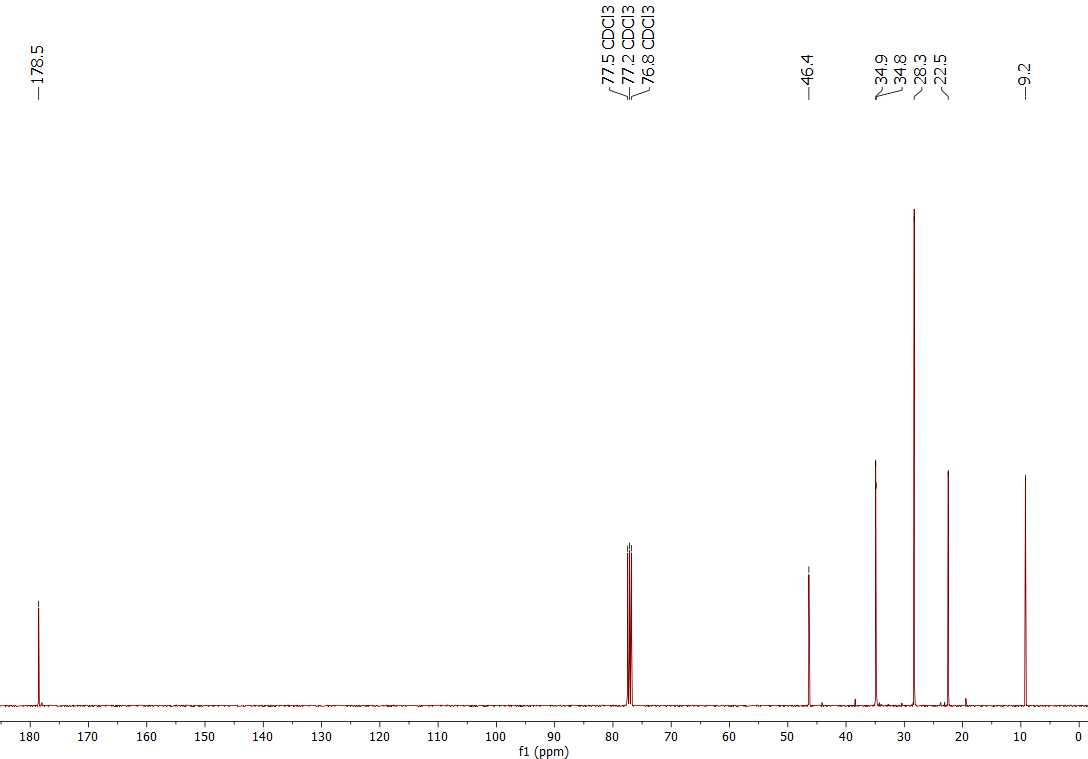


## Methyl 2-(tert-pentylthio)acetate (3n)


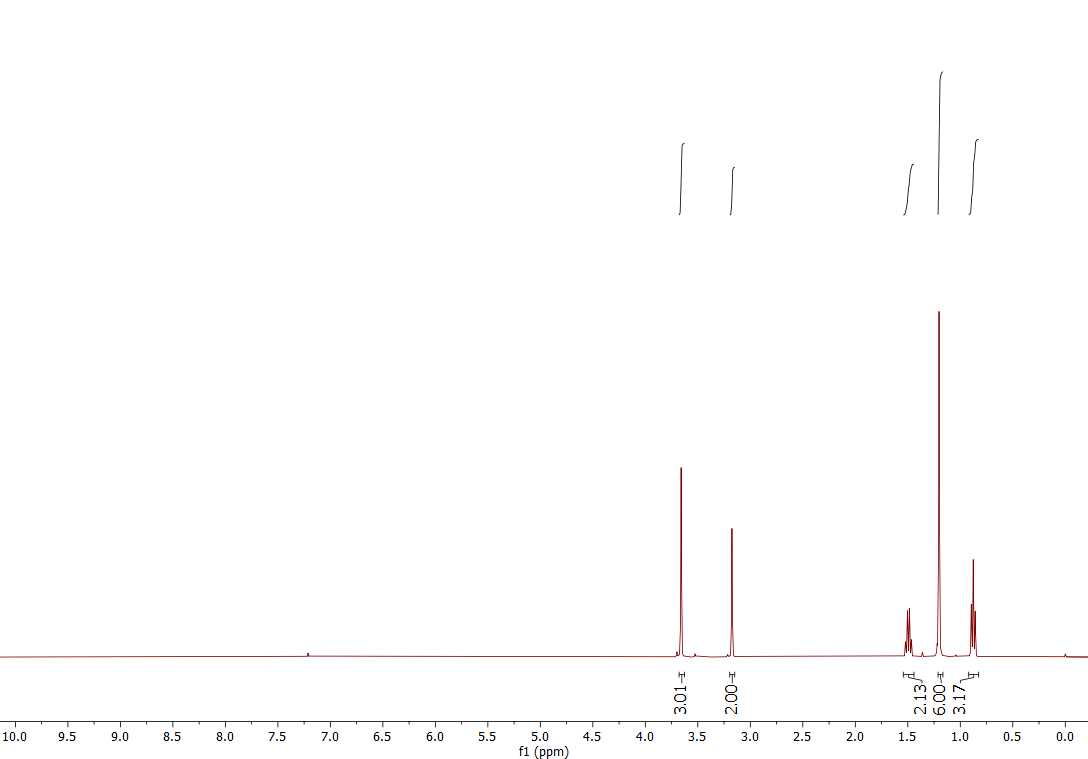


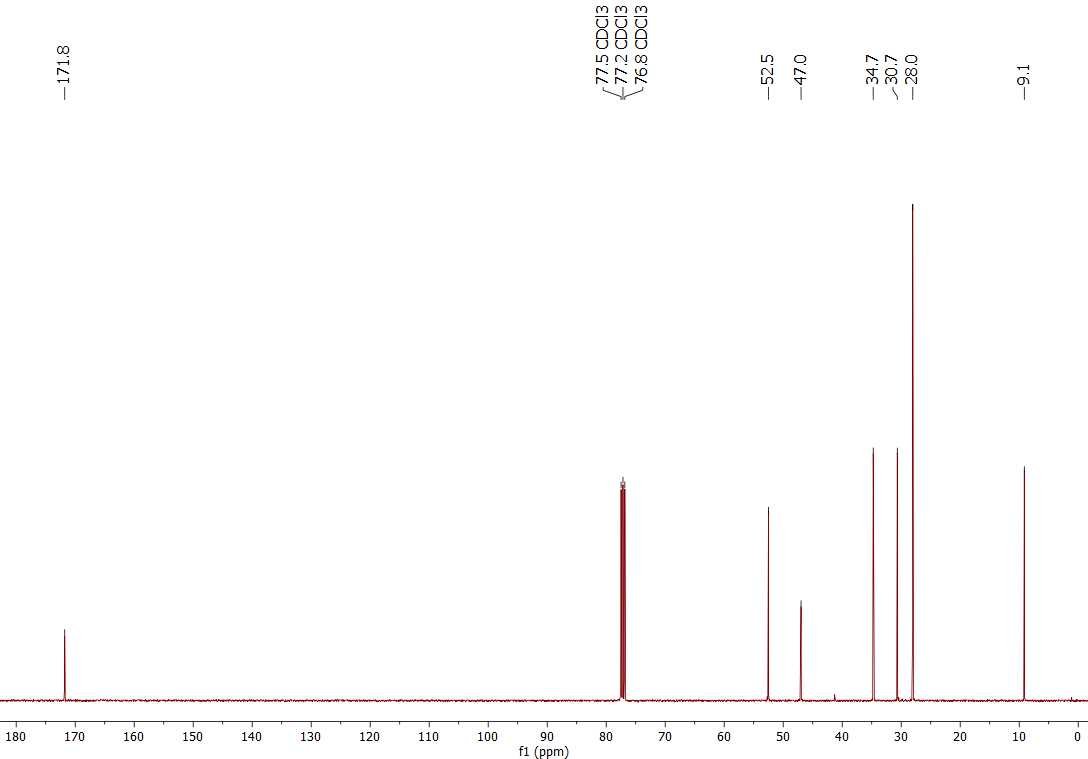


## (4-Chlorobenzyl)(tert-pentyl)sulfane (3o)


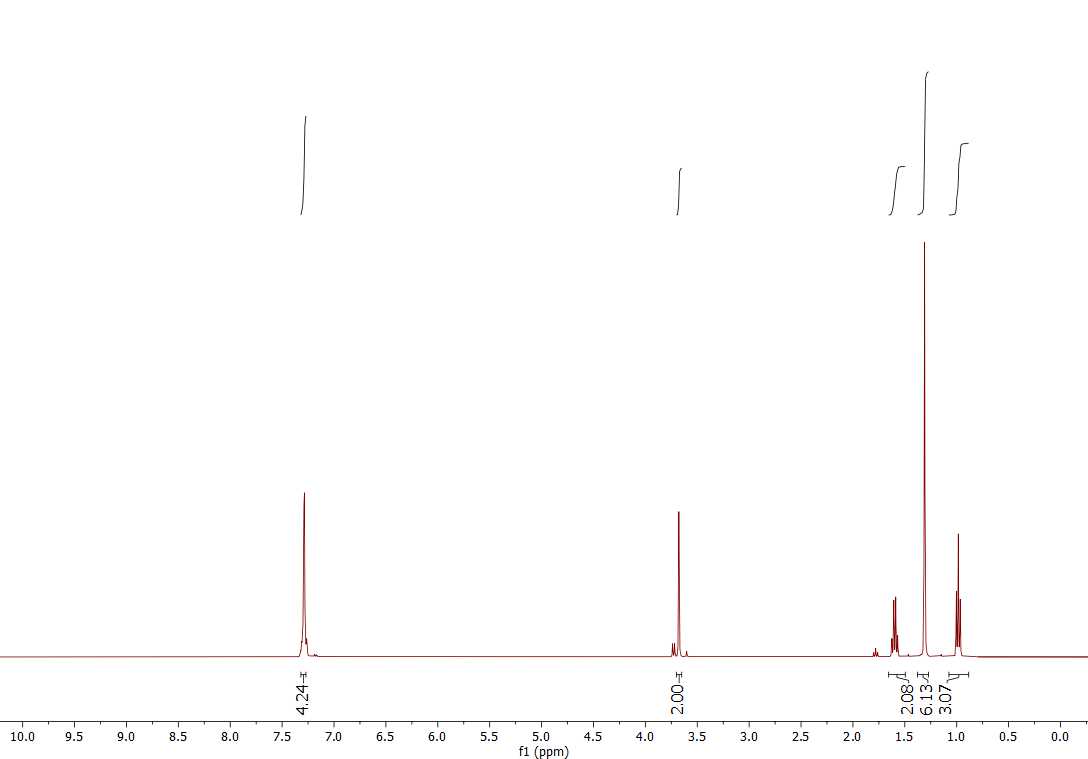


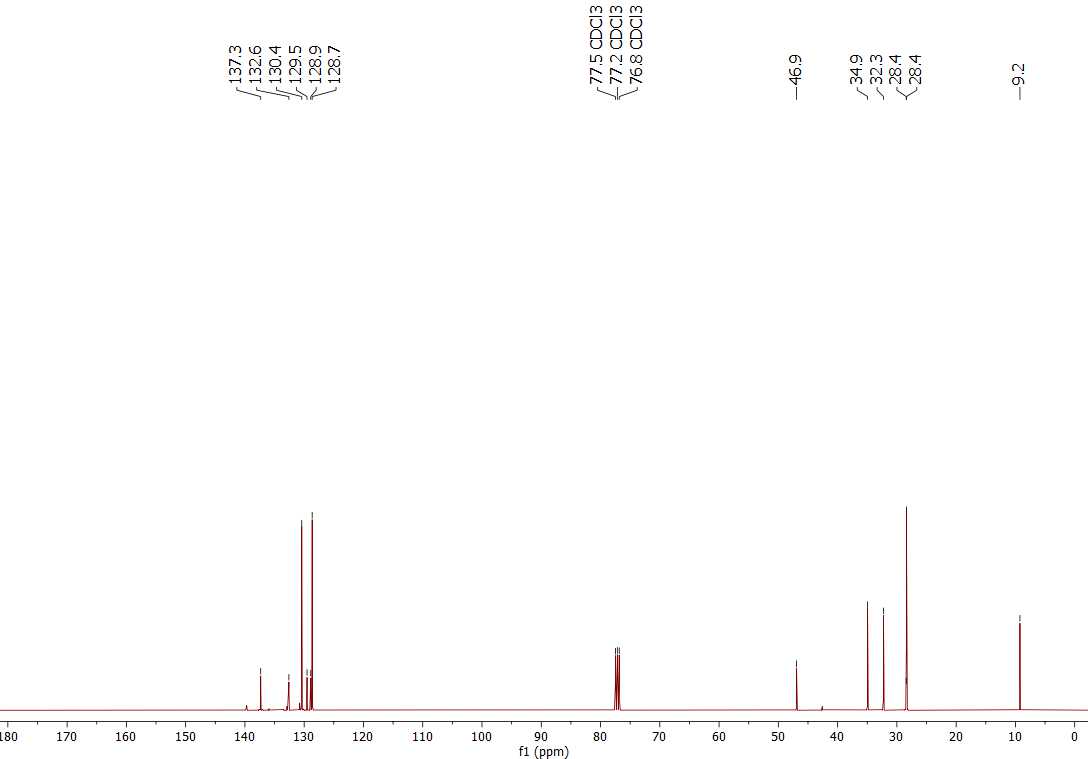


## Octyl(tert-pentyl)sulfane (3p)


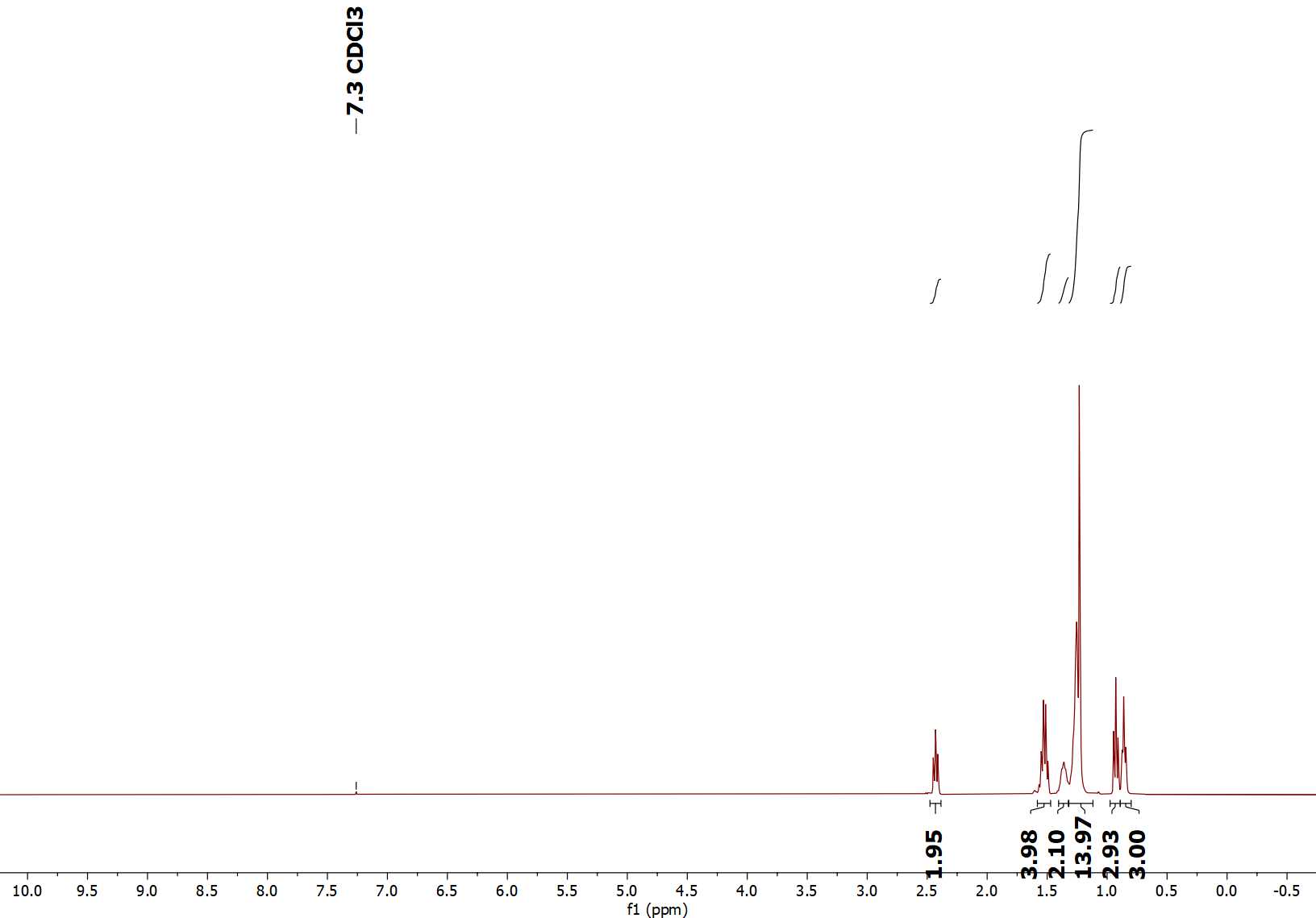


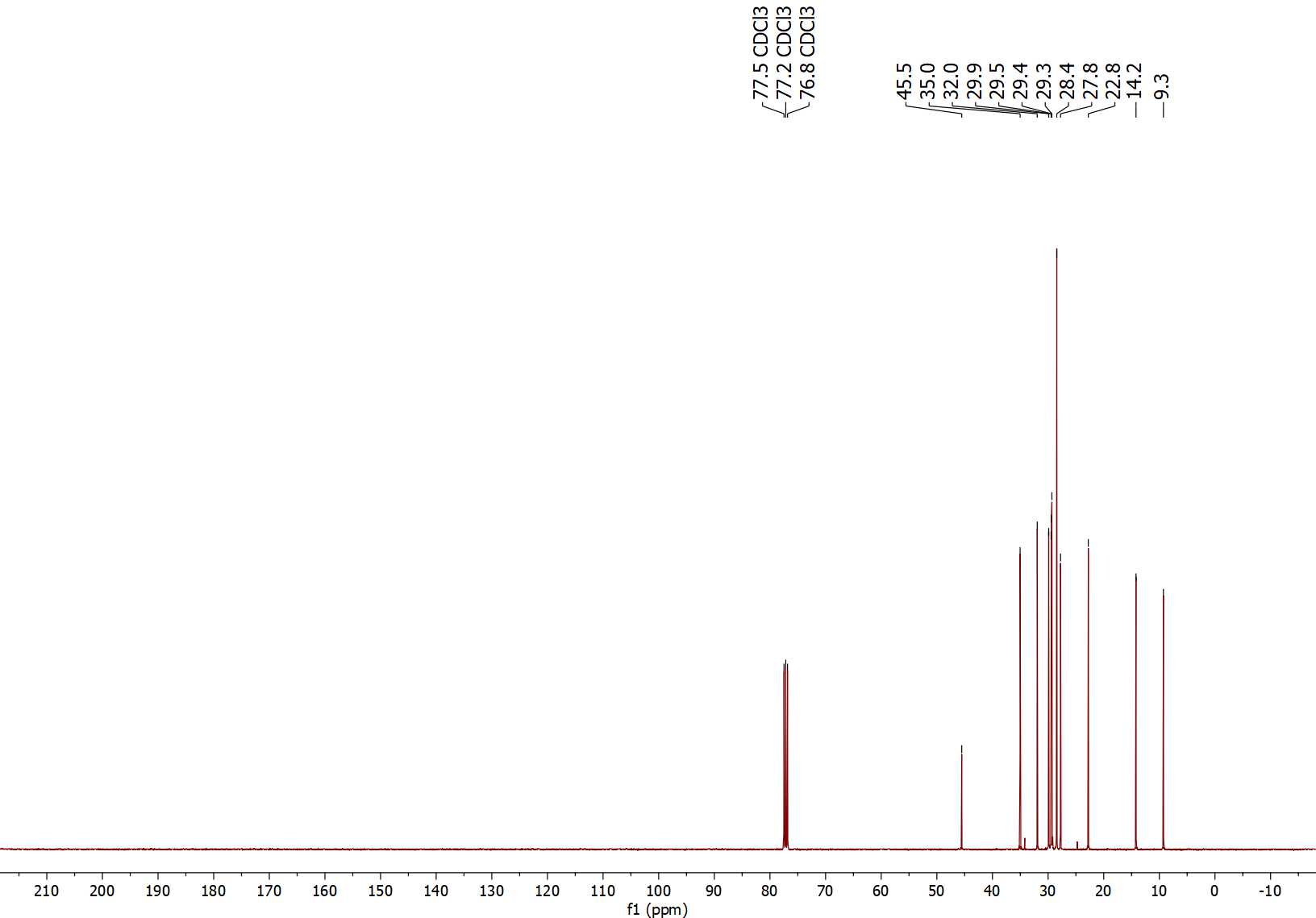


## 1,6-Bis(tert-pentylthio)hexane (3q)


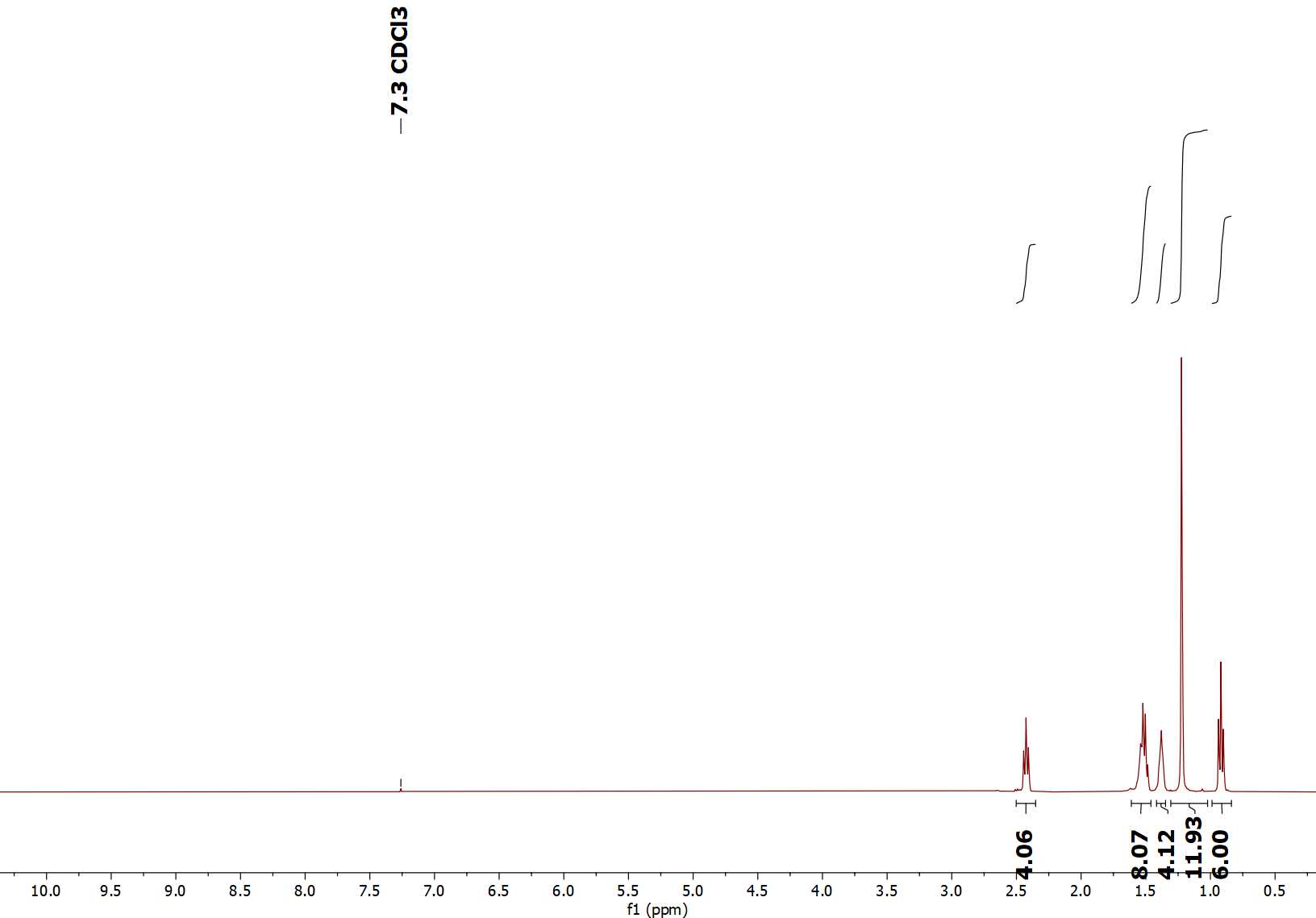


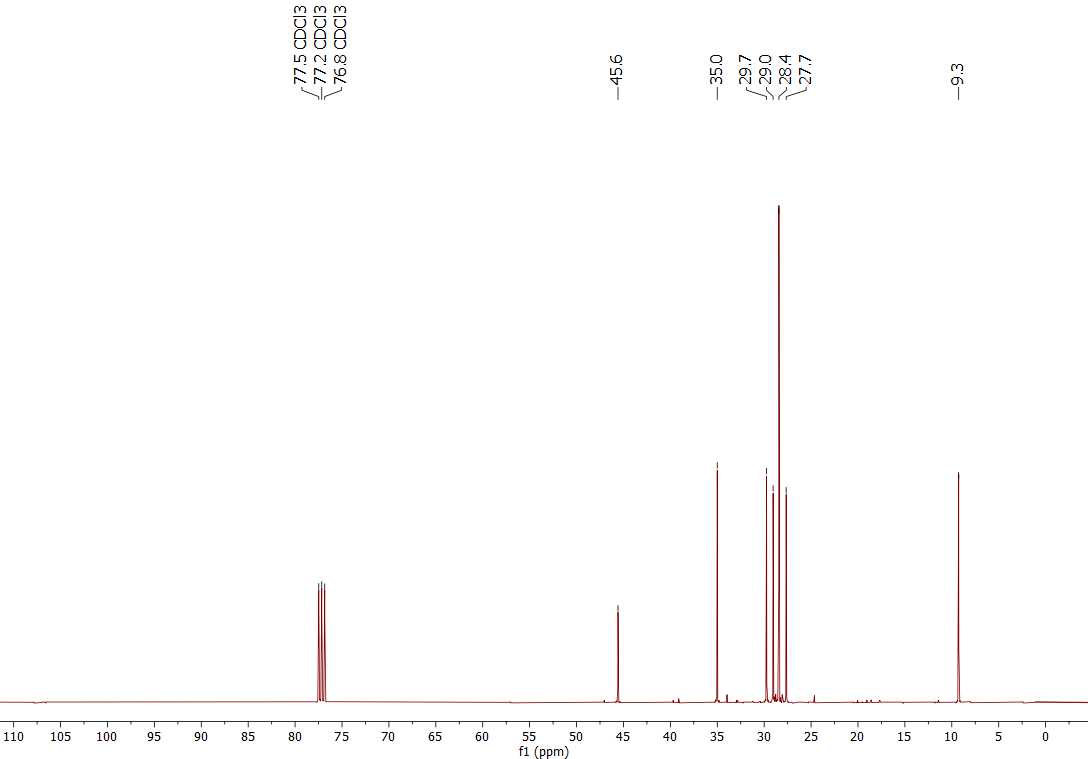


## 1,3-Bis(tert-pentylthio)propane (3r)


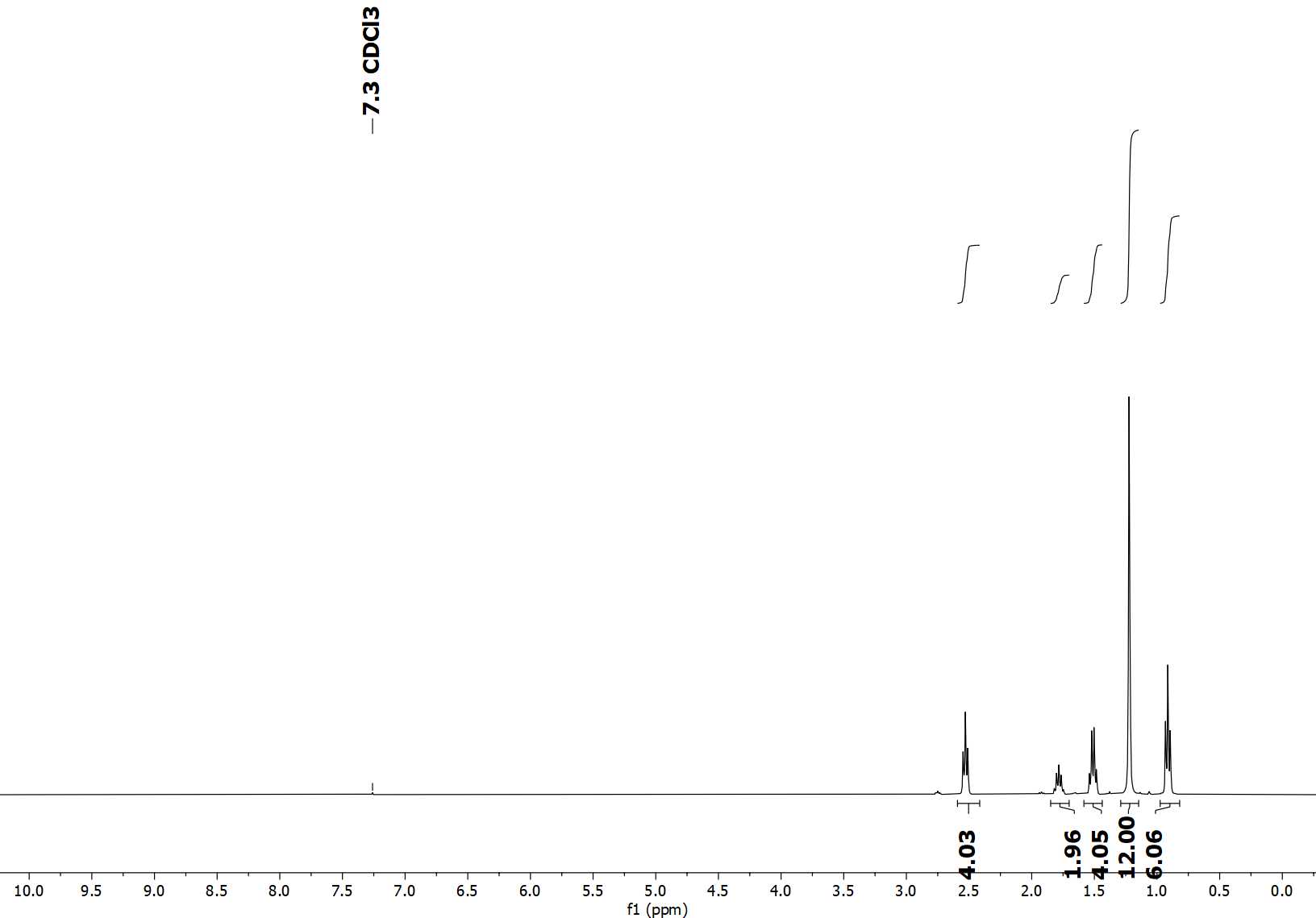


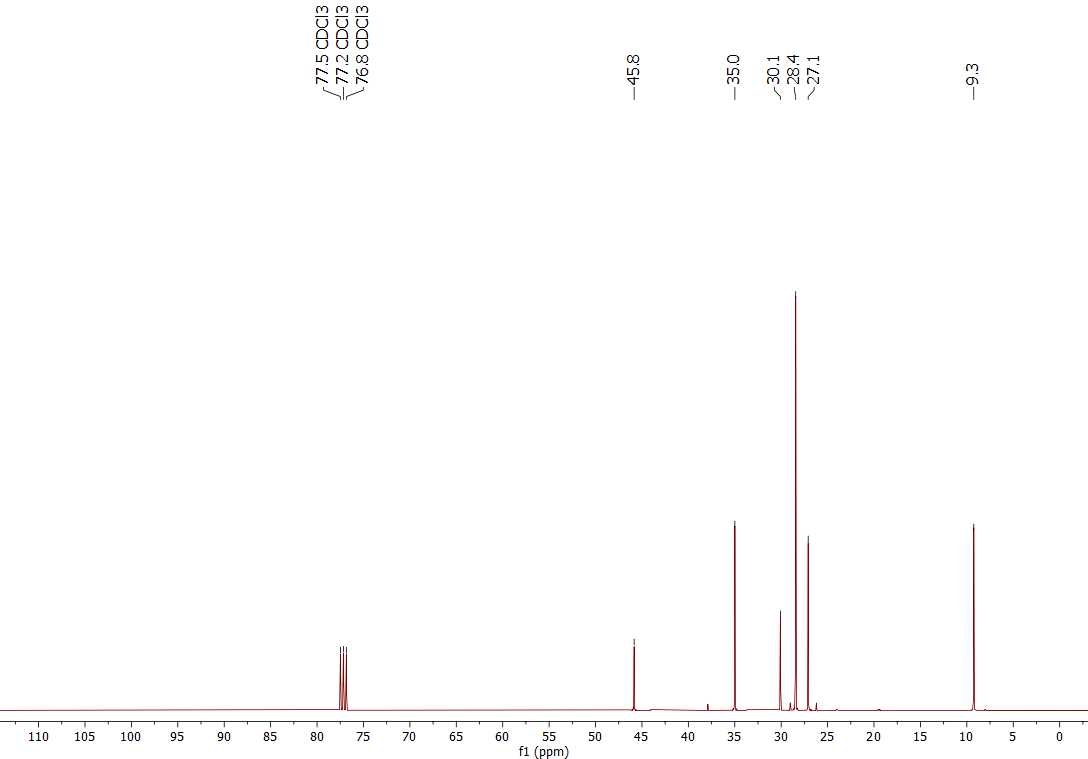


## (3-Ethylpentan-3-yl)(phenyl)sulfane (3aa)


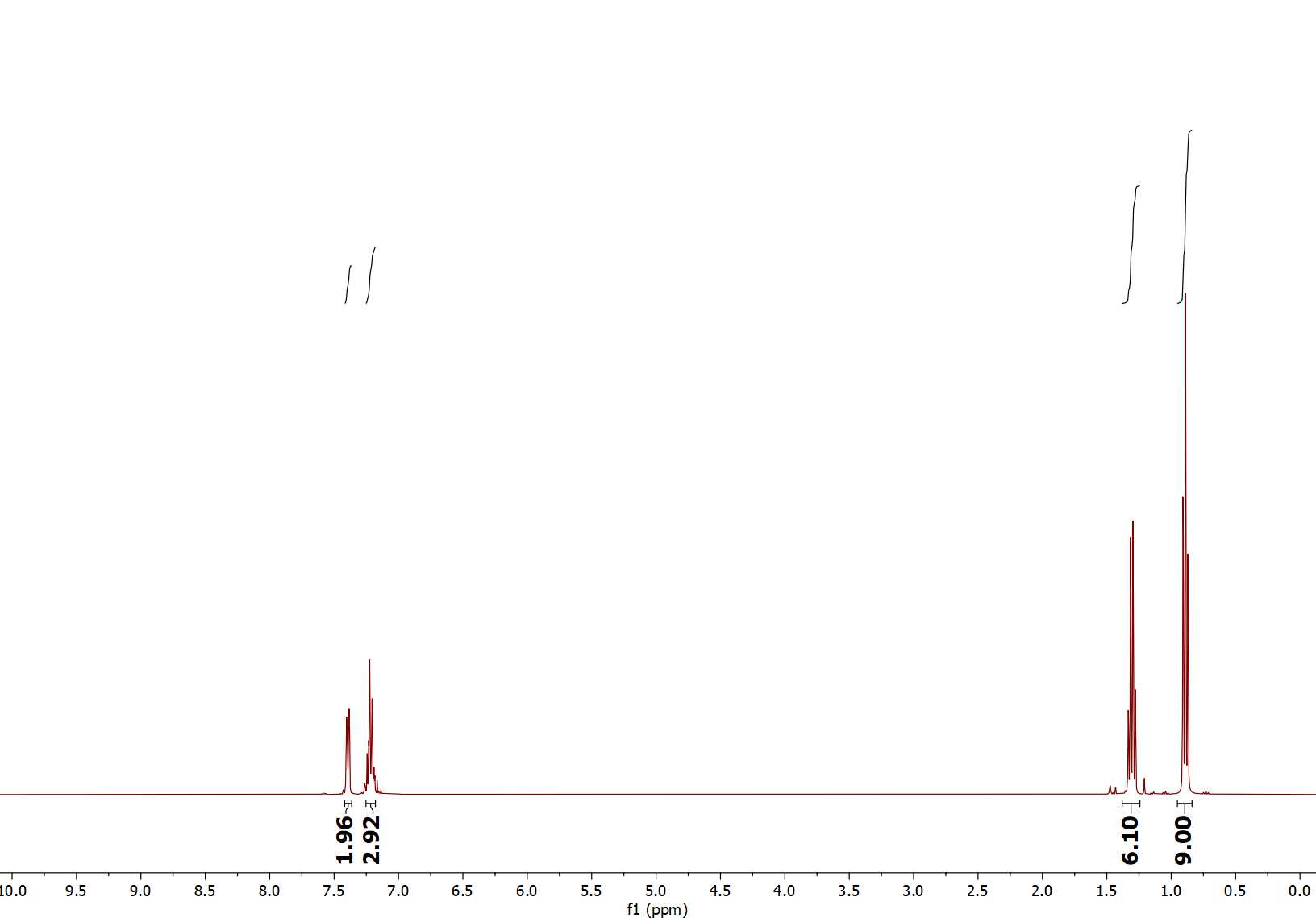


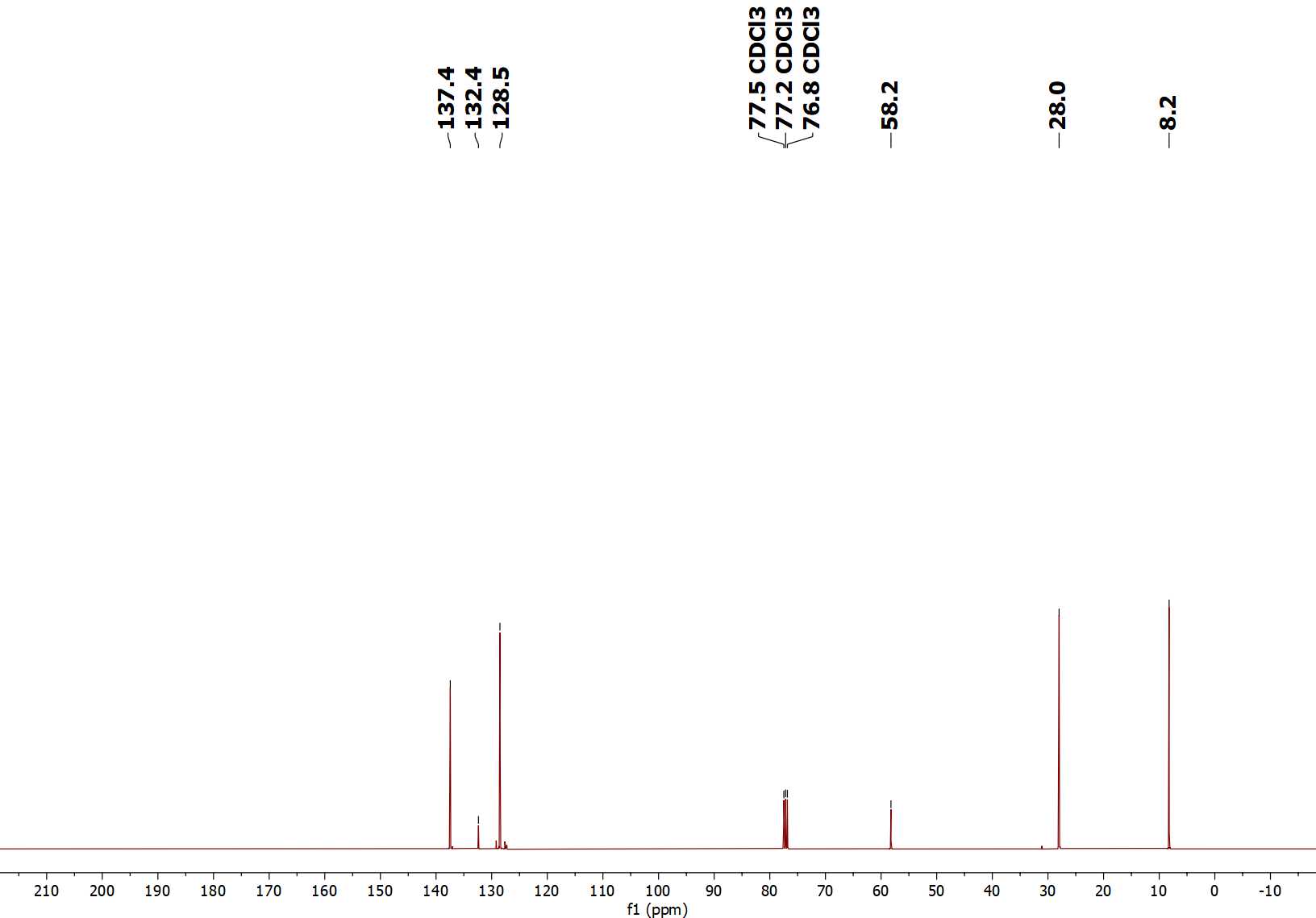


## ((3s,5s,7s)-adamantan-1-yl)(phenyl)sulfane (3ab)


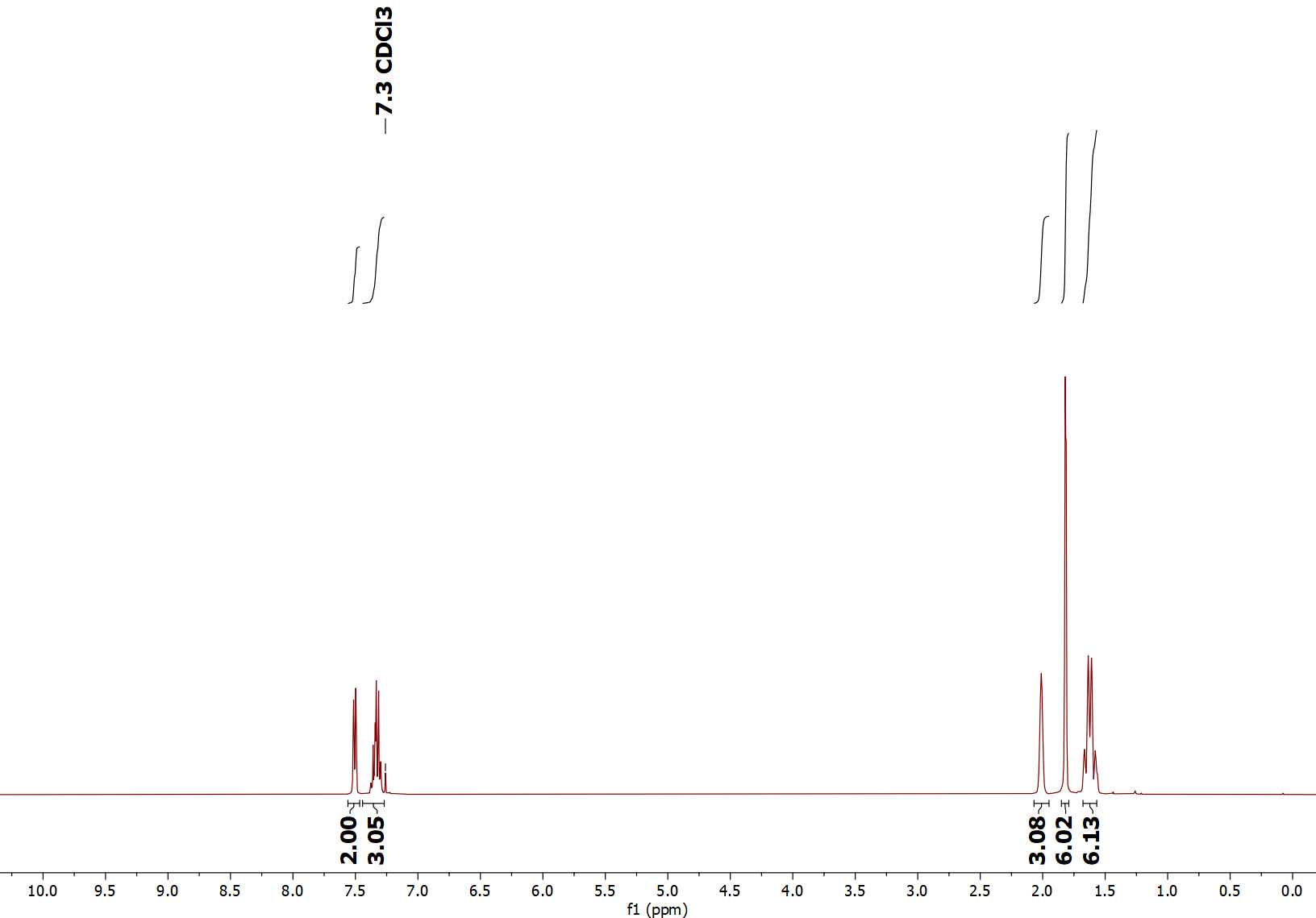


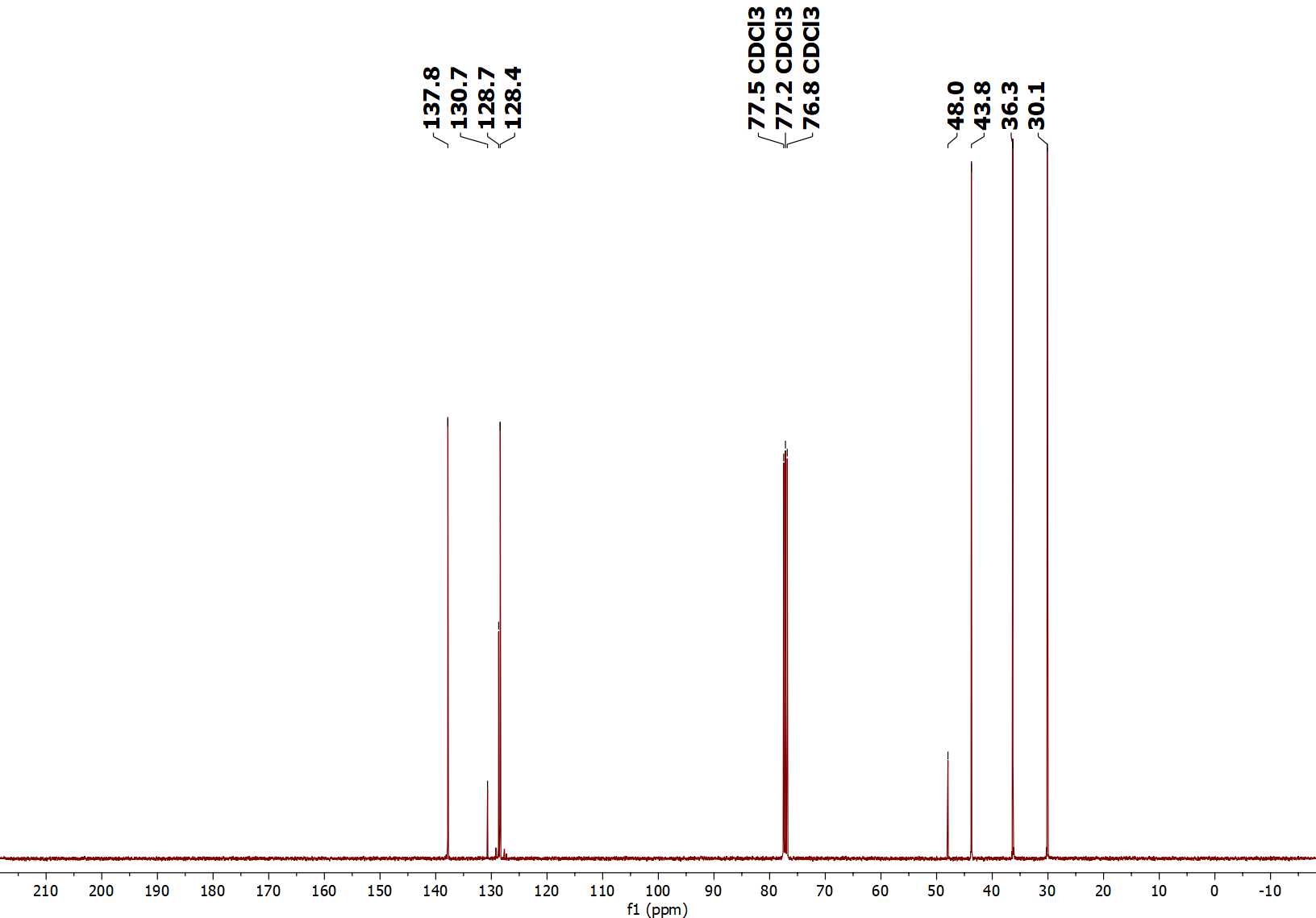


## (1-Methylcyclopentyl)(phenyl)sulfane (3ac)


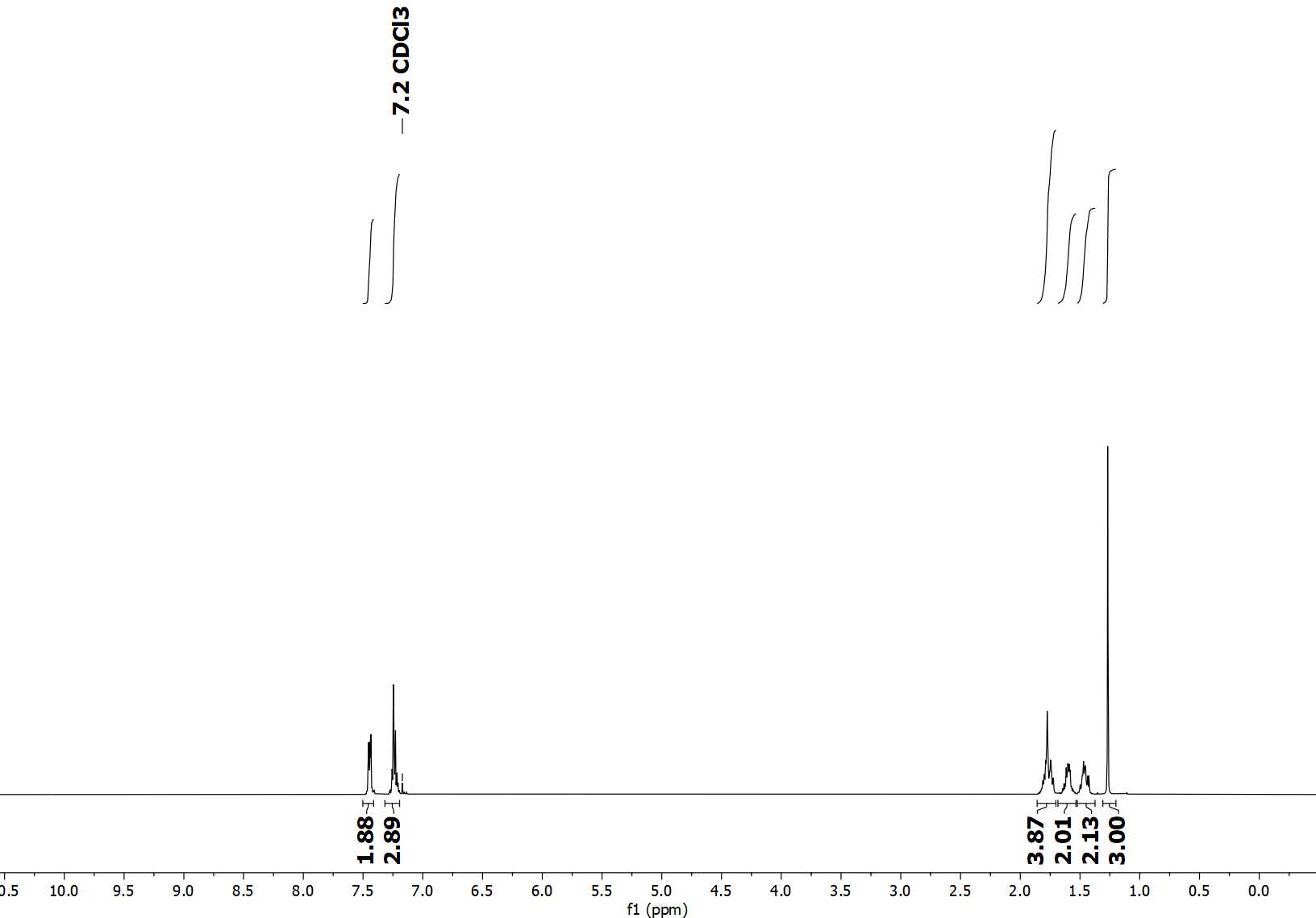


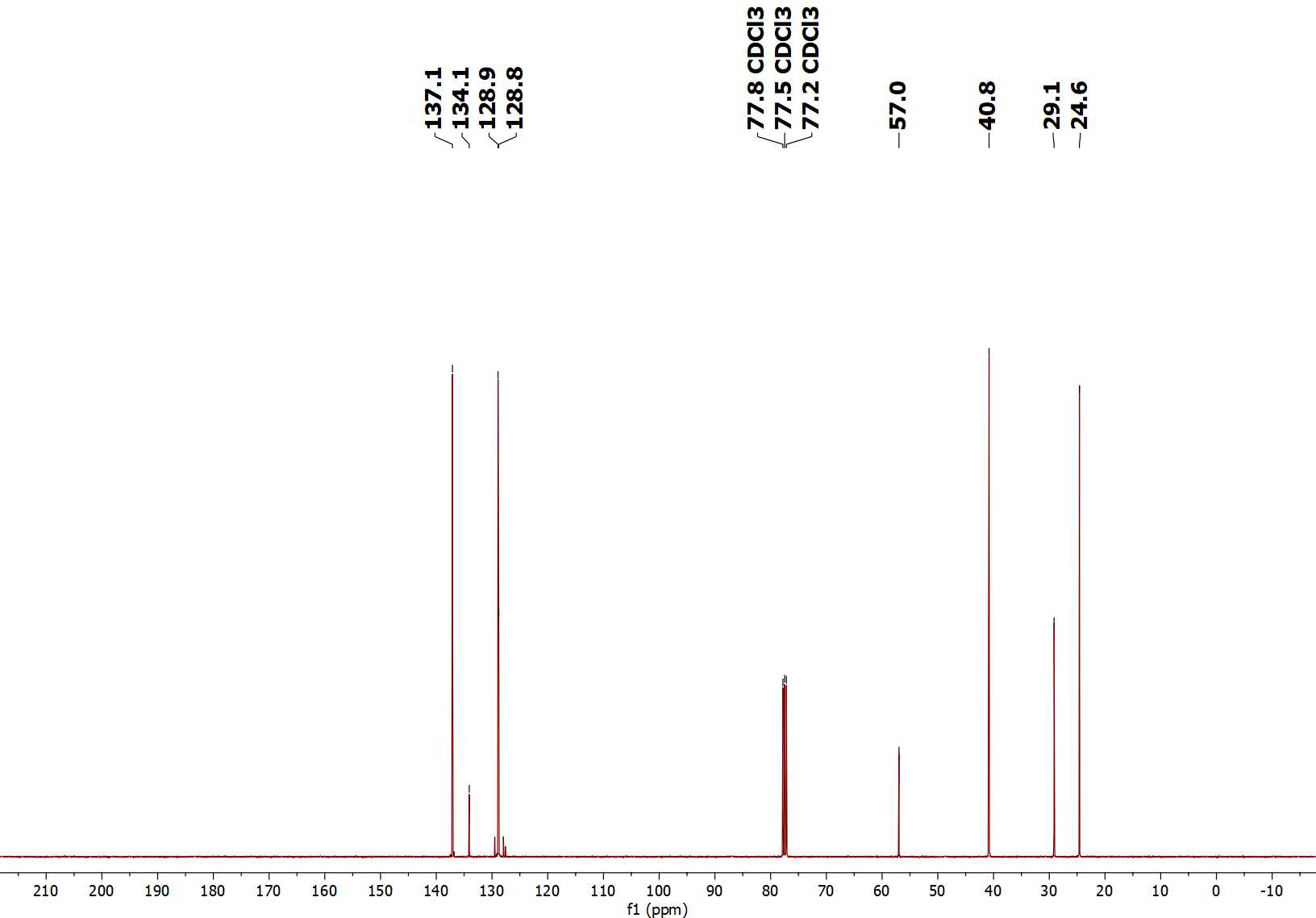


## 4-Methyl-4-(phenylthio)pentan-2-one (3ad)


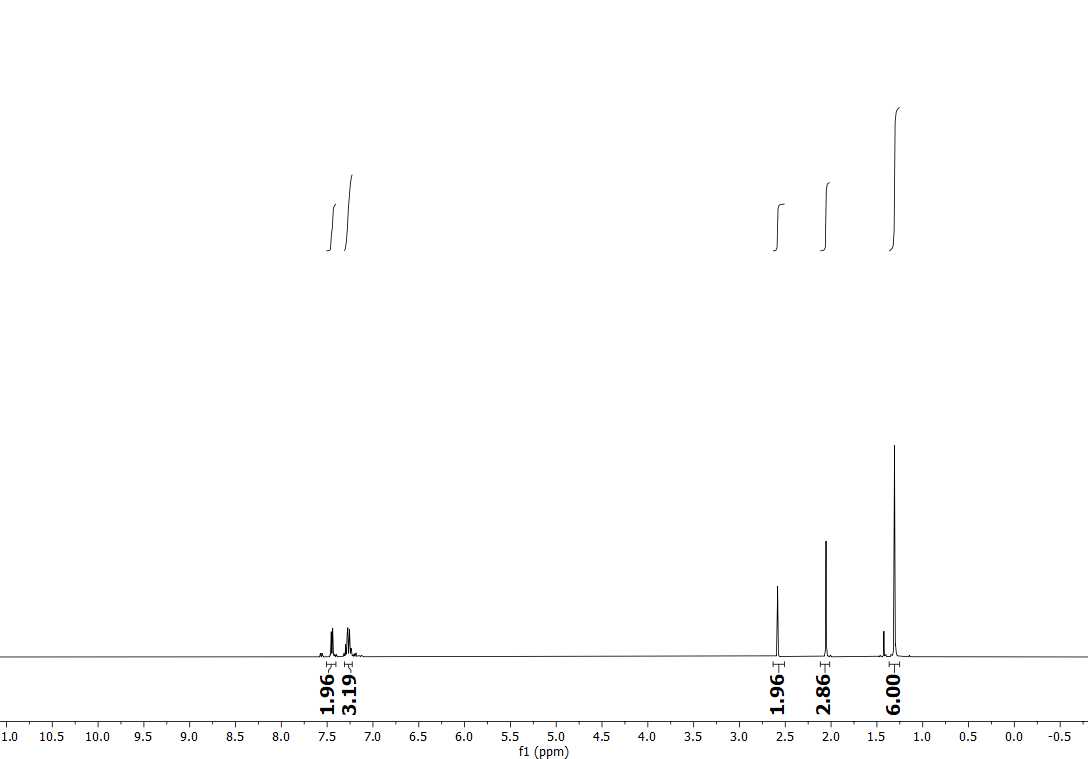


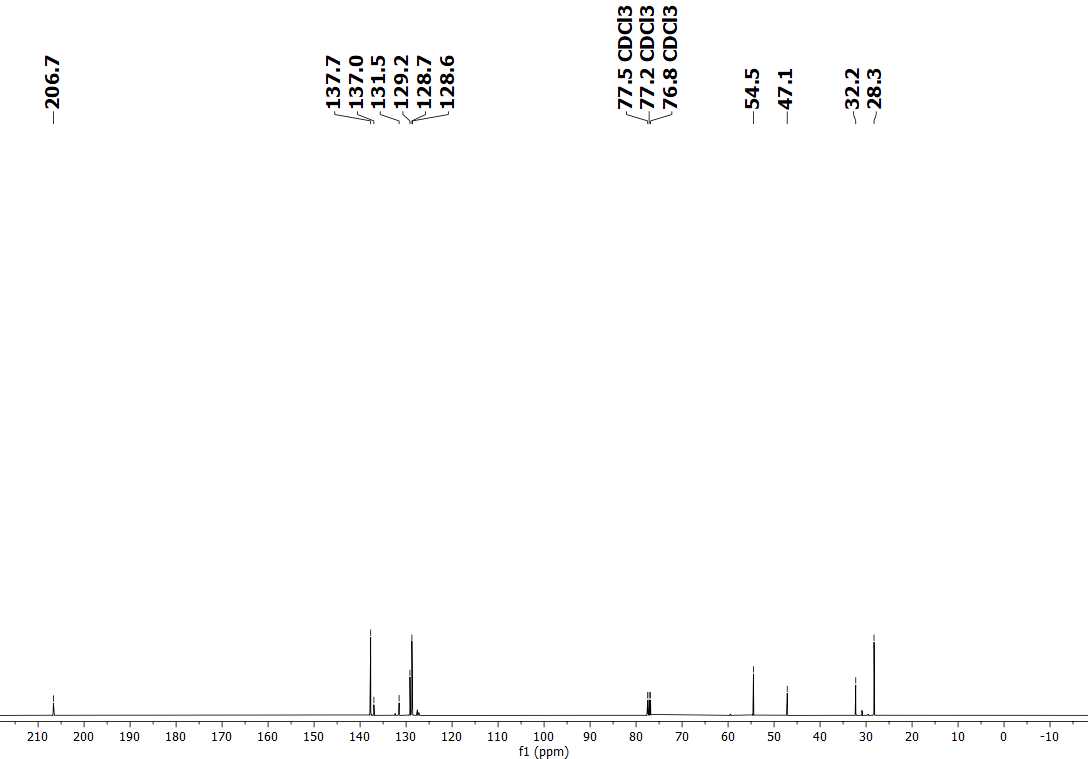


## Cyclohex-2-en-1-yl(phenyl)sulfane (3ae)


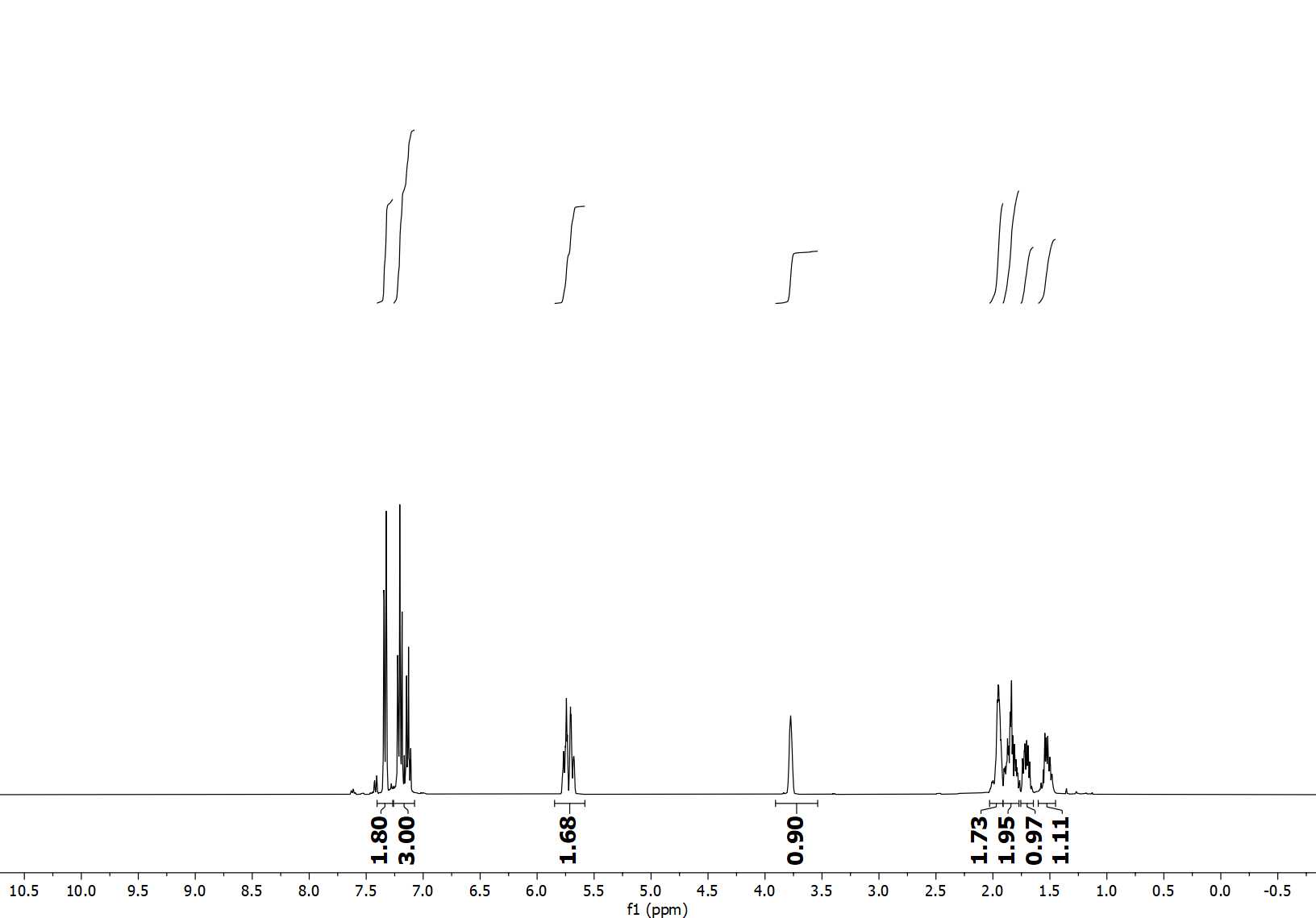


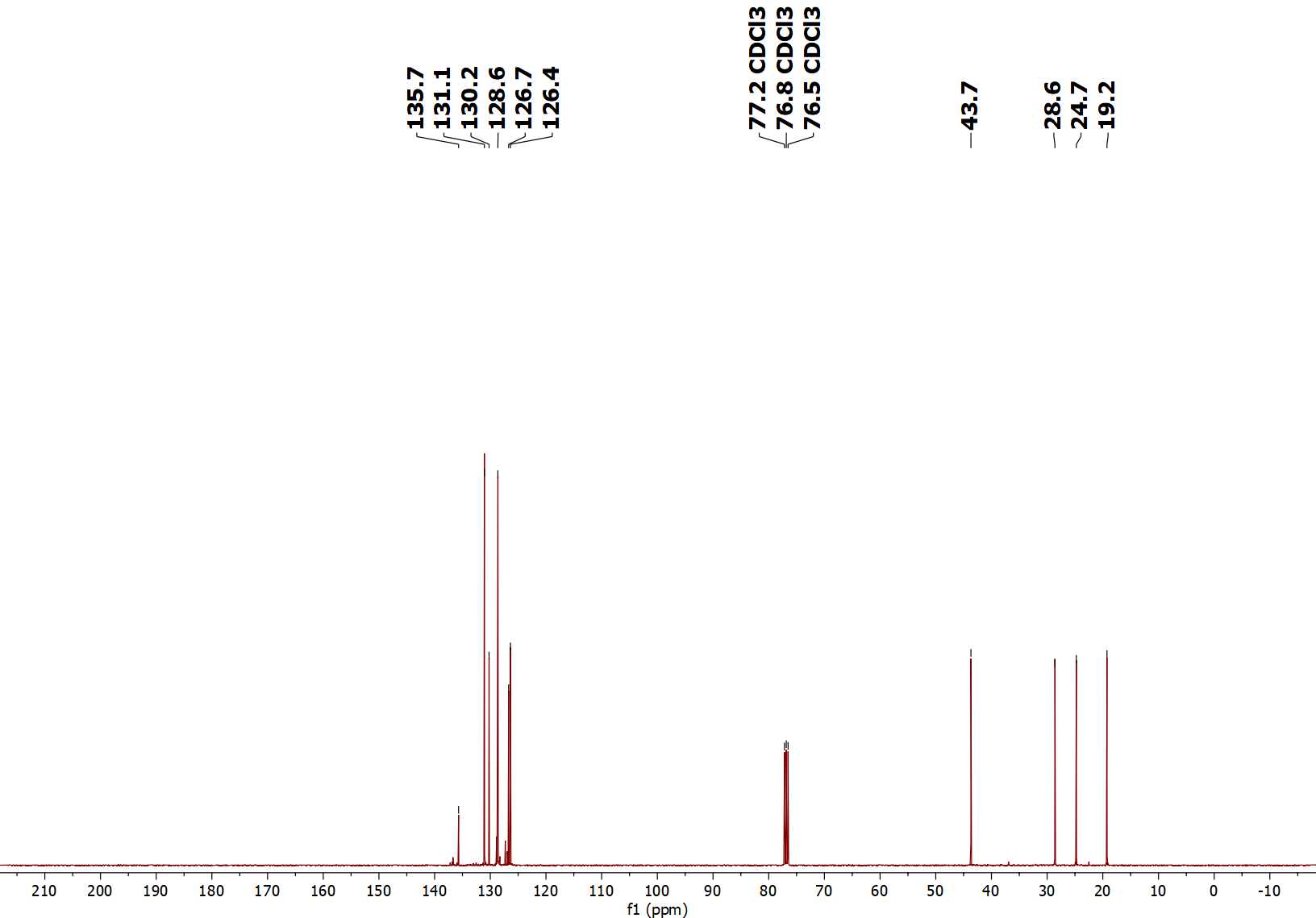


## (9H-fluoren-9-yl)(phenyl)sulfane (3af)


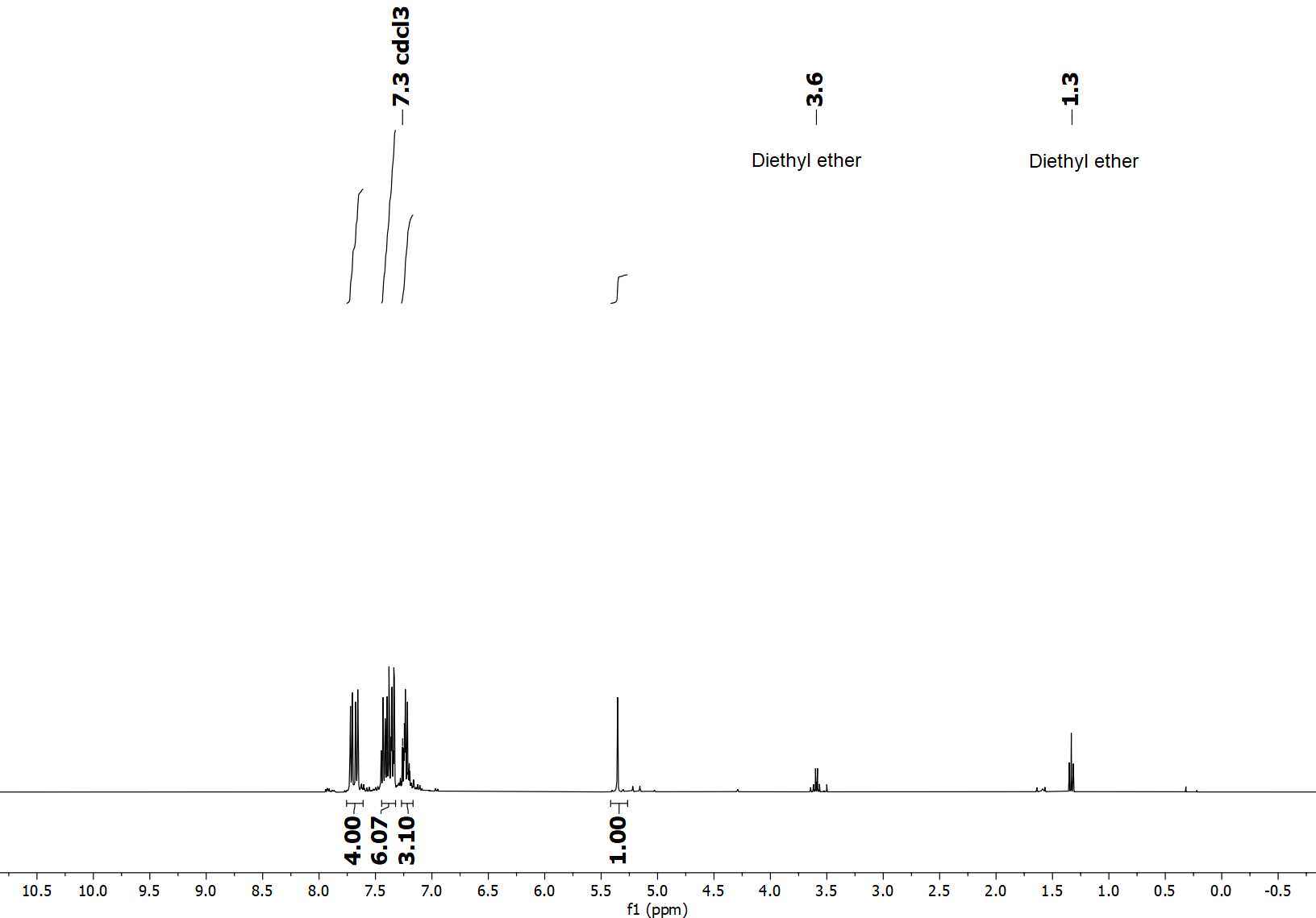


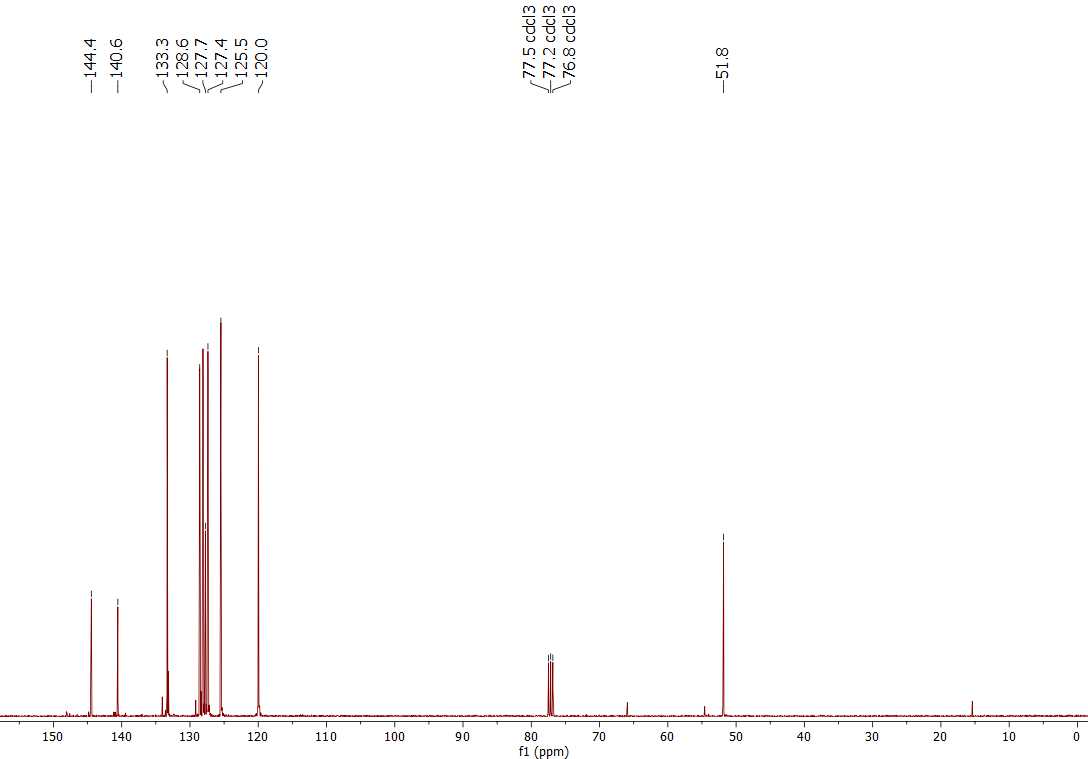


## Benzhydryl(phenyl)sulfane (3ag)


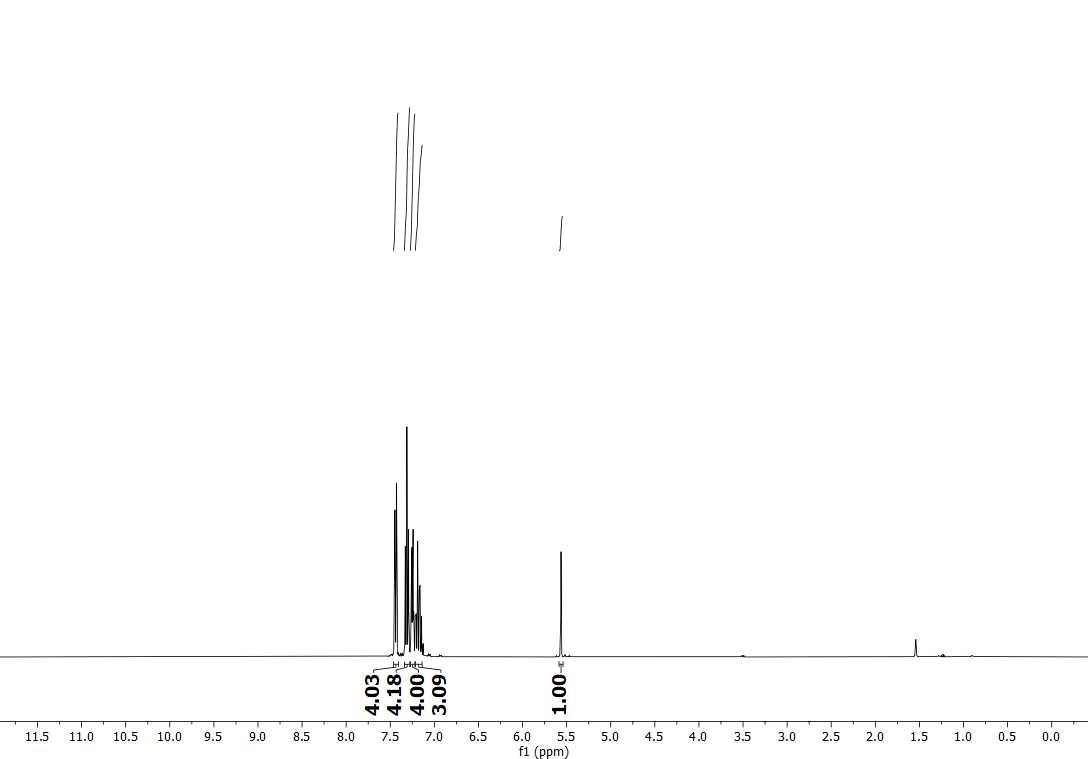


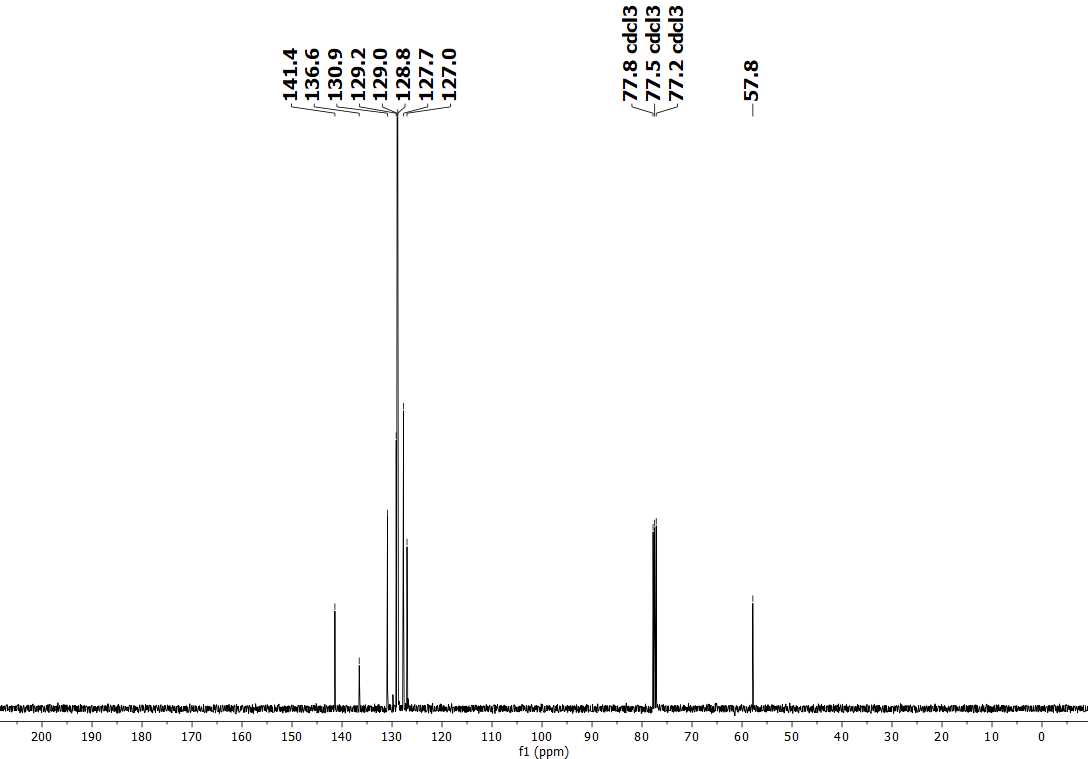


## Phenyl(1-phenylpropyl)sulfane (3ah)


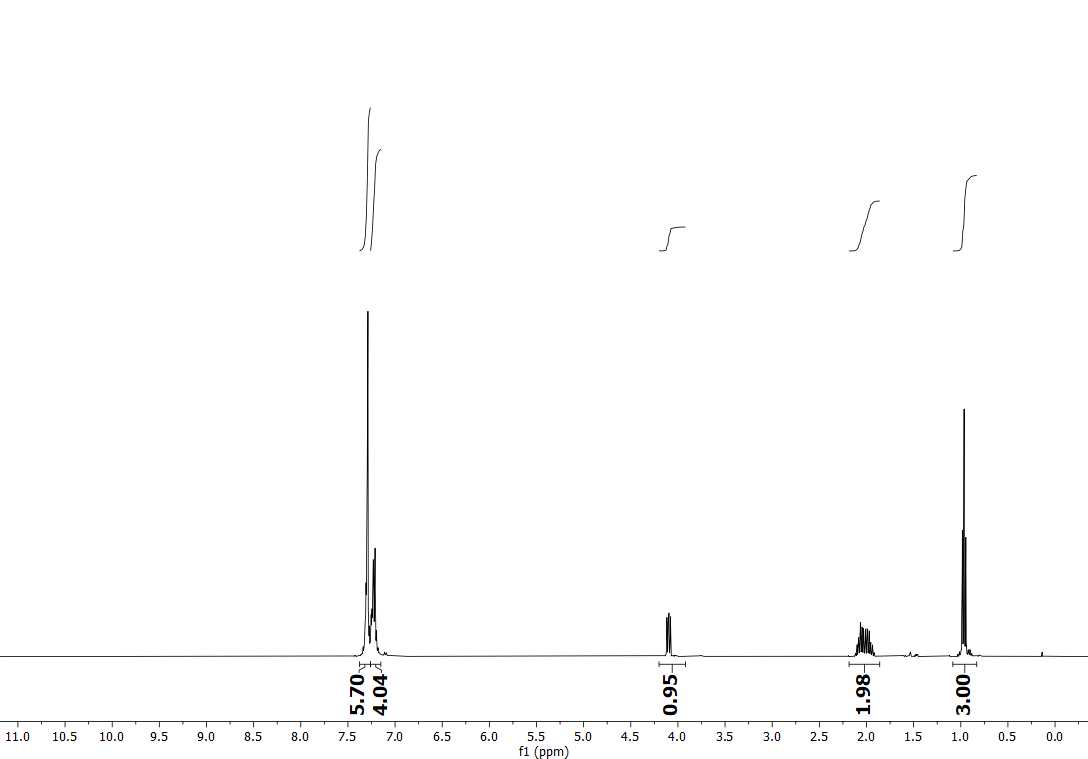


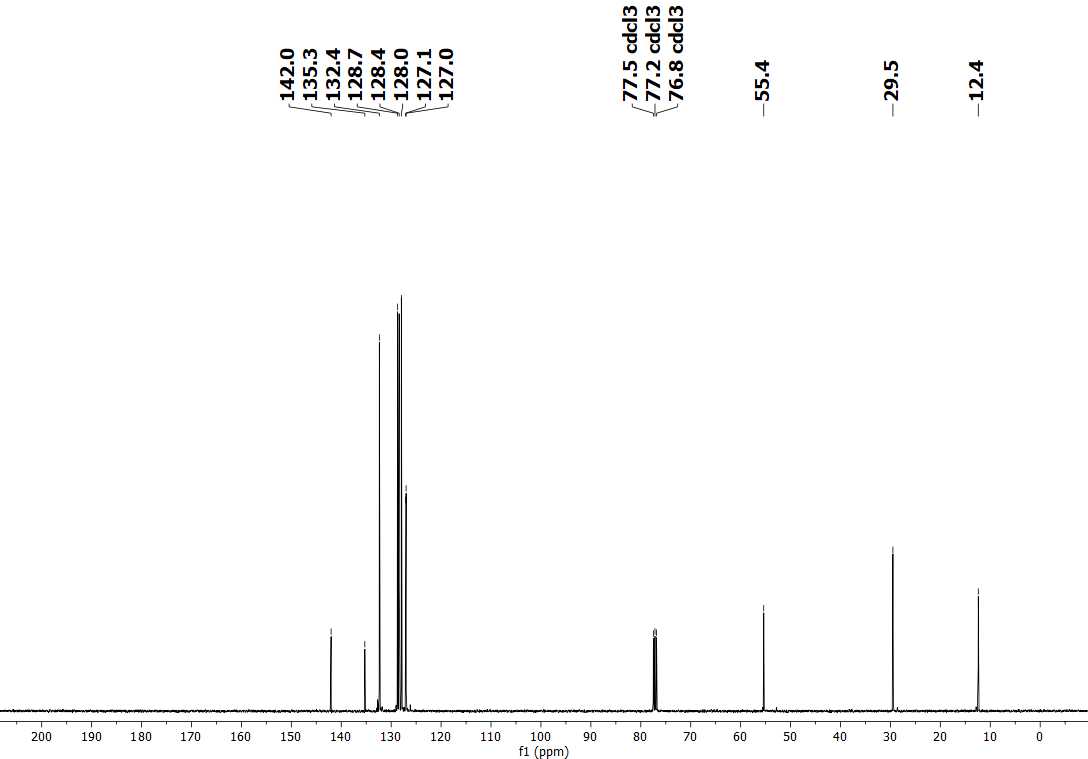


## Phenyl(1-phenylethyl)sulfane (3ai)


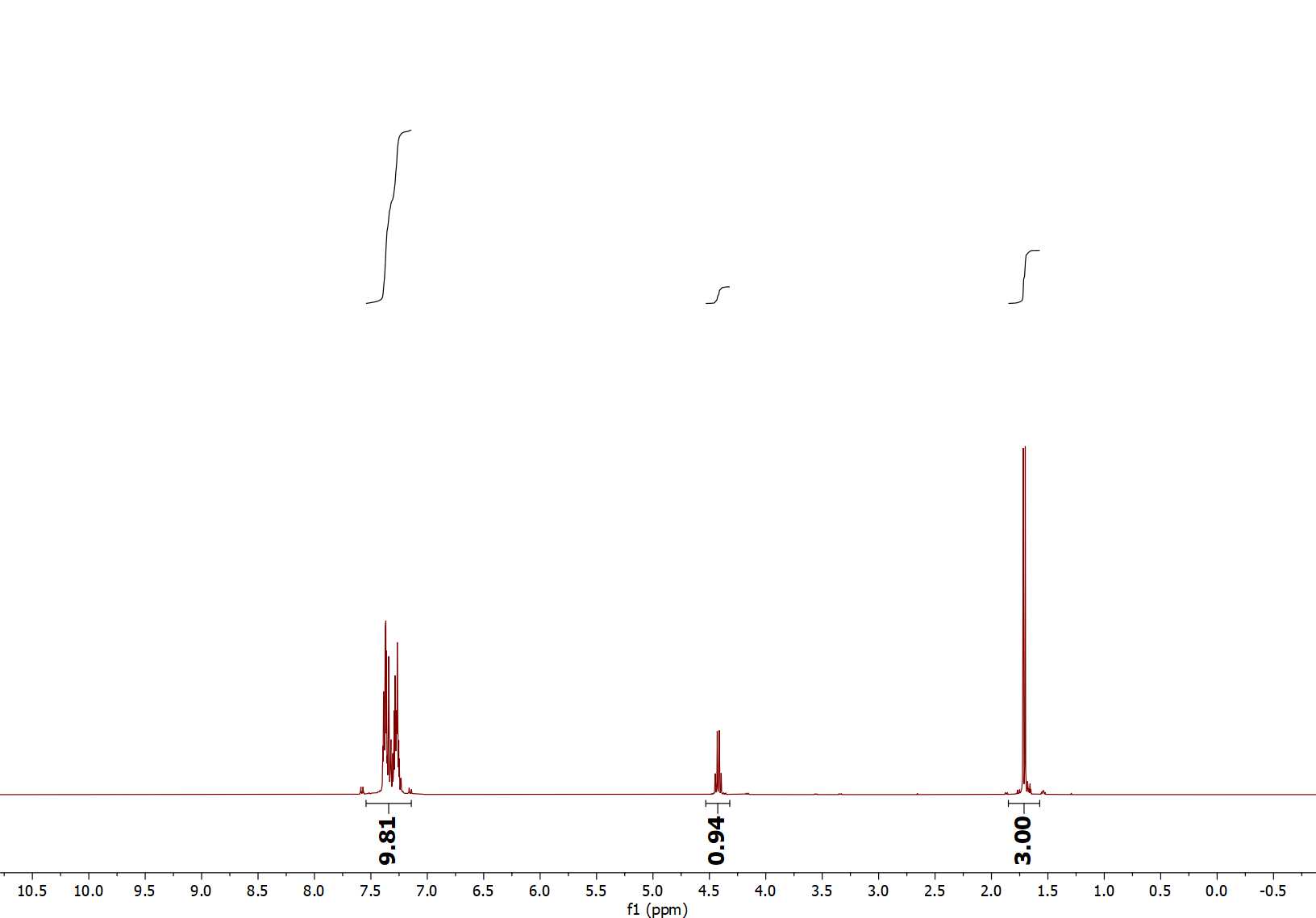


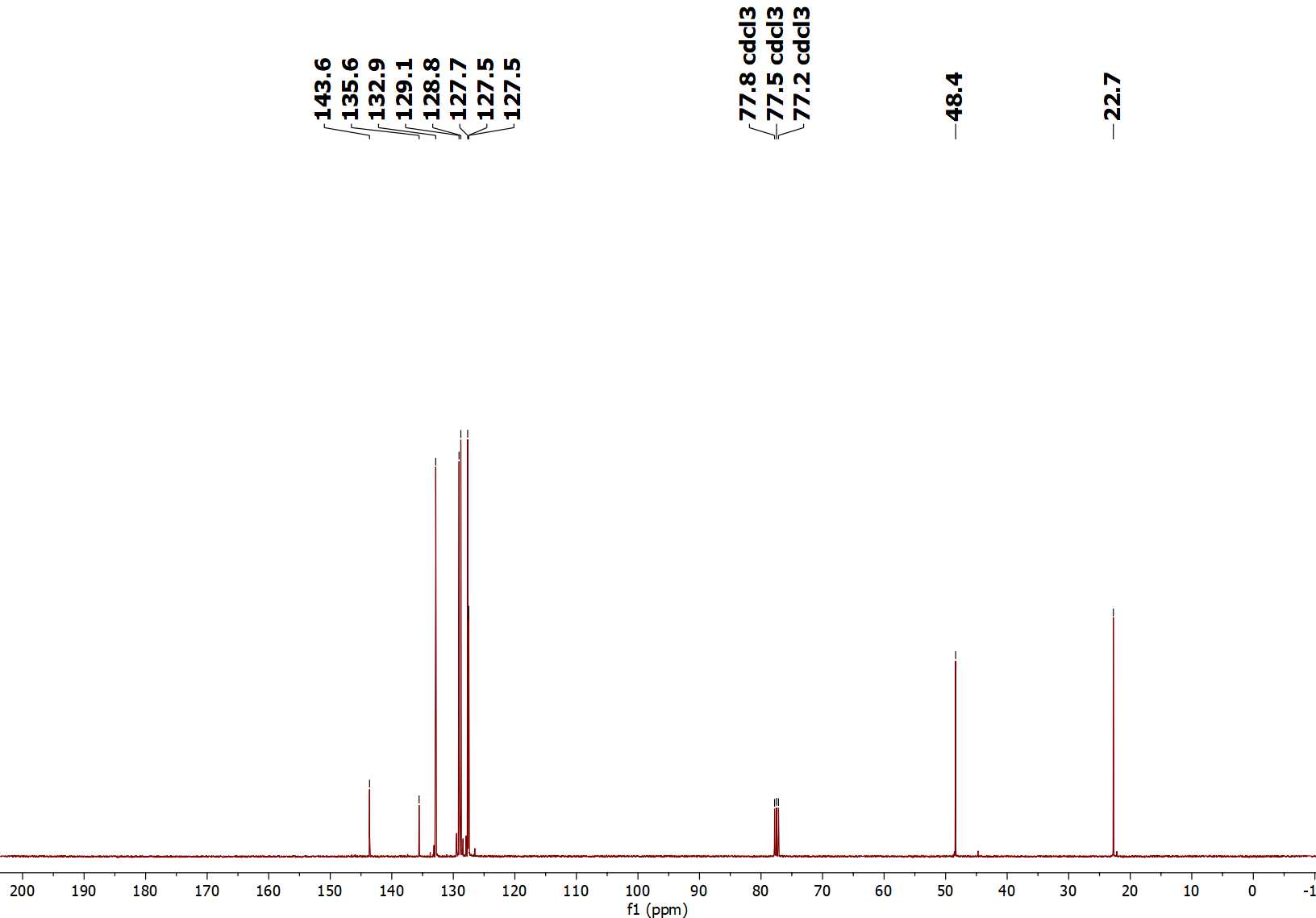


## Phenyl(1-(p-tolyl)ethyl)sulfane (3aj)


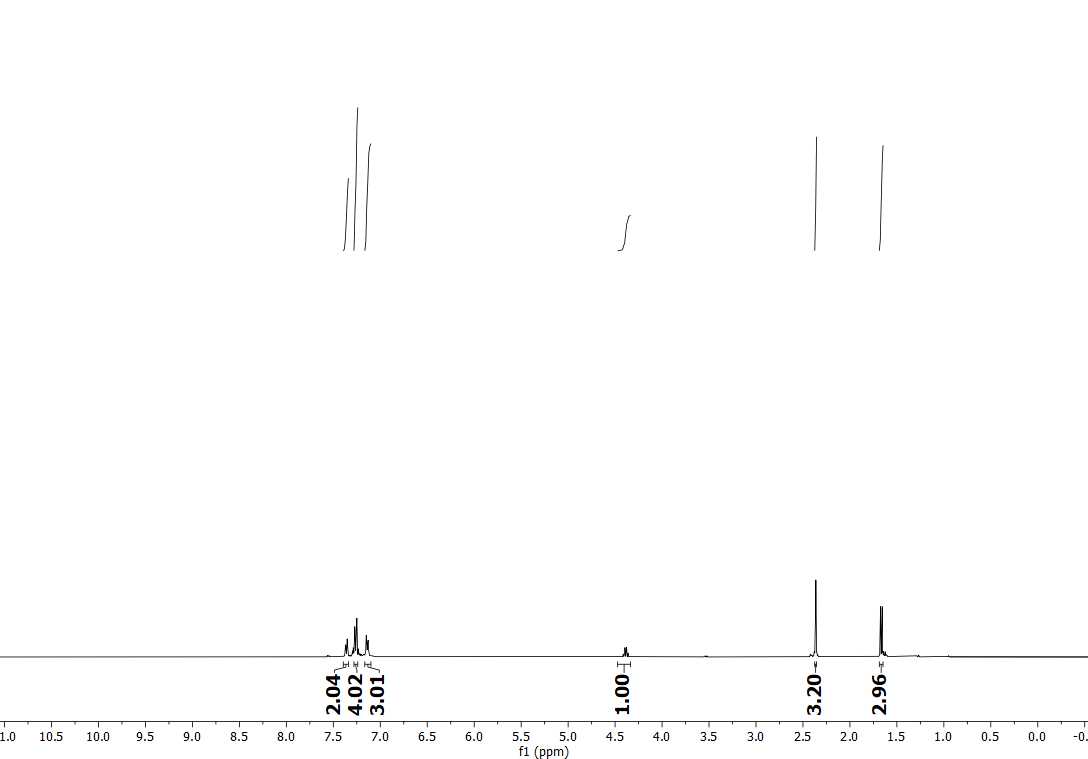


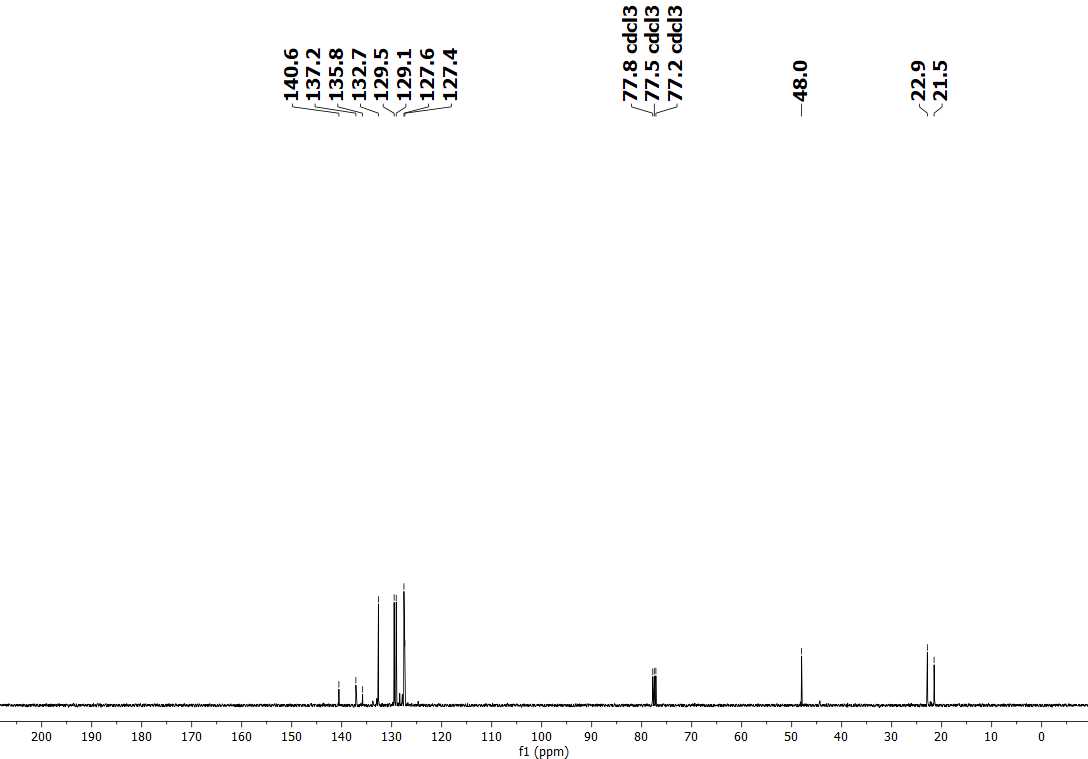


## (1-(4-Methoxyphenyl)ethyl)(phenyl)sulfane (3ak)


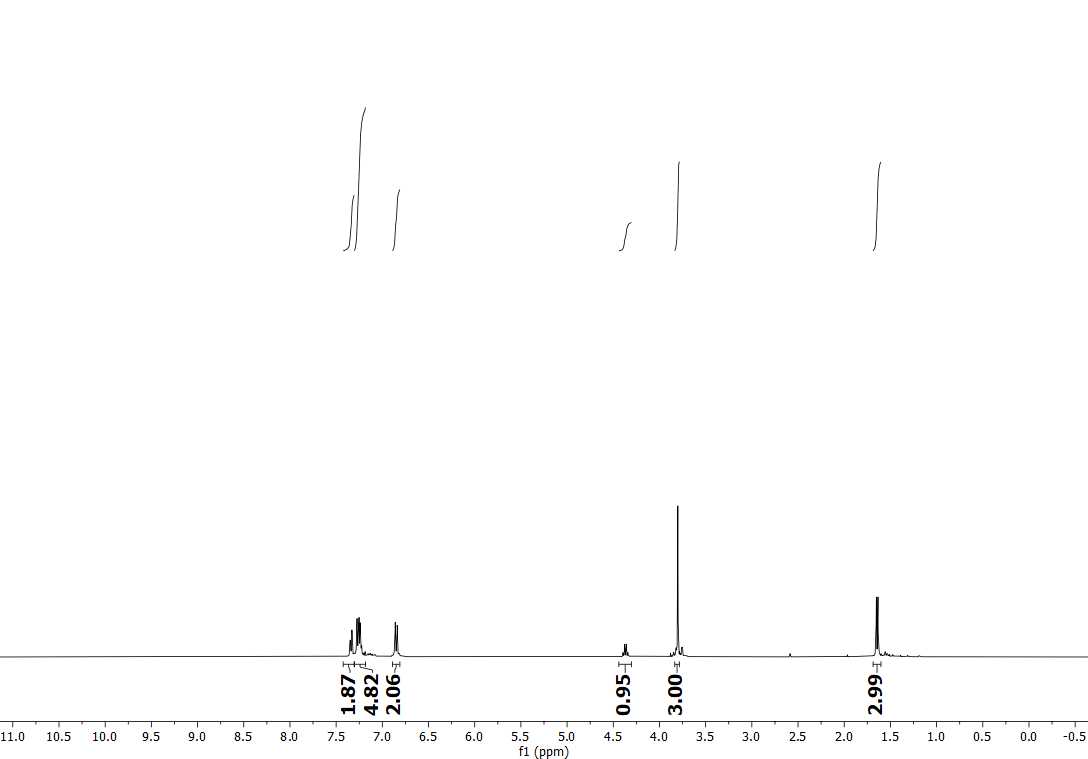


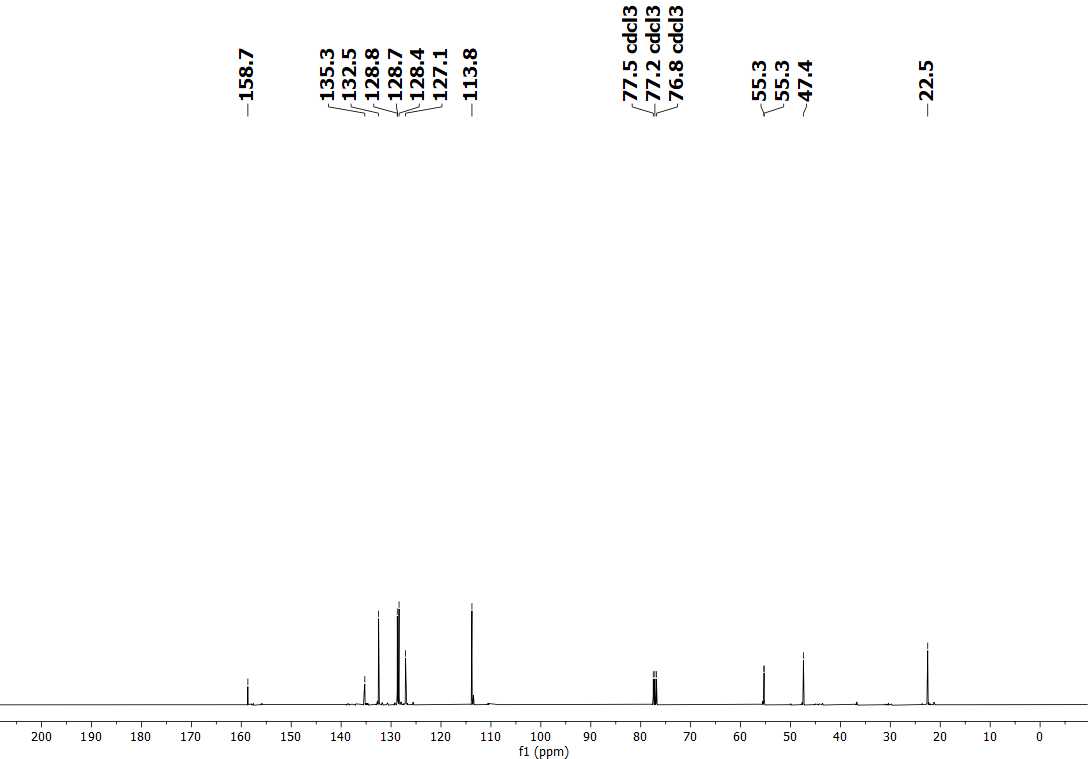


## (1-(4-Fluorophenyl)ethyl)(phenyl)sulfane (3al)


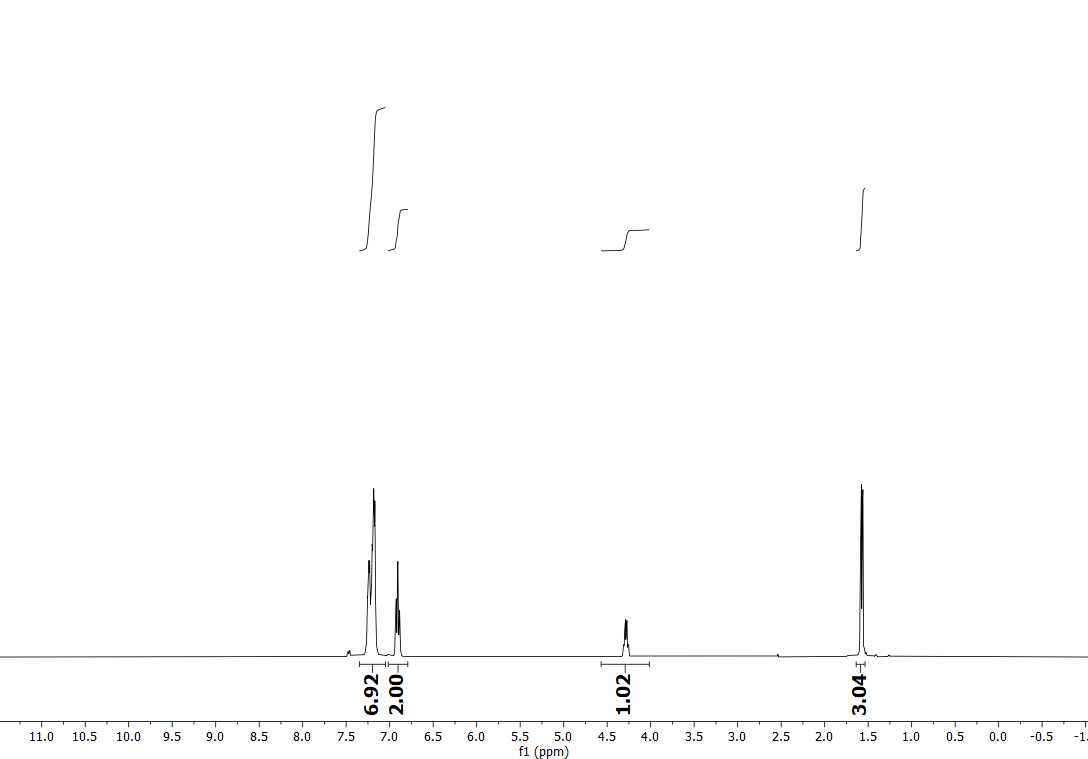


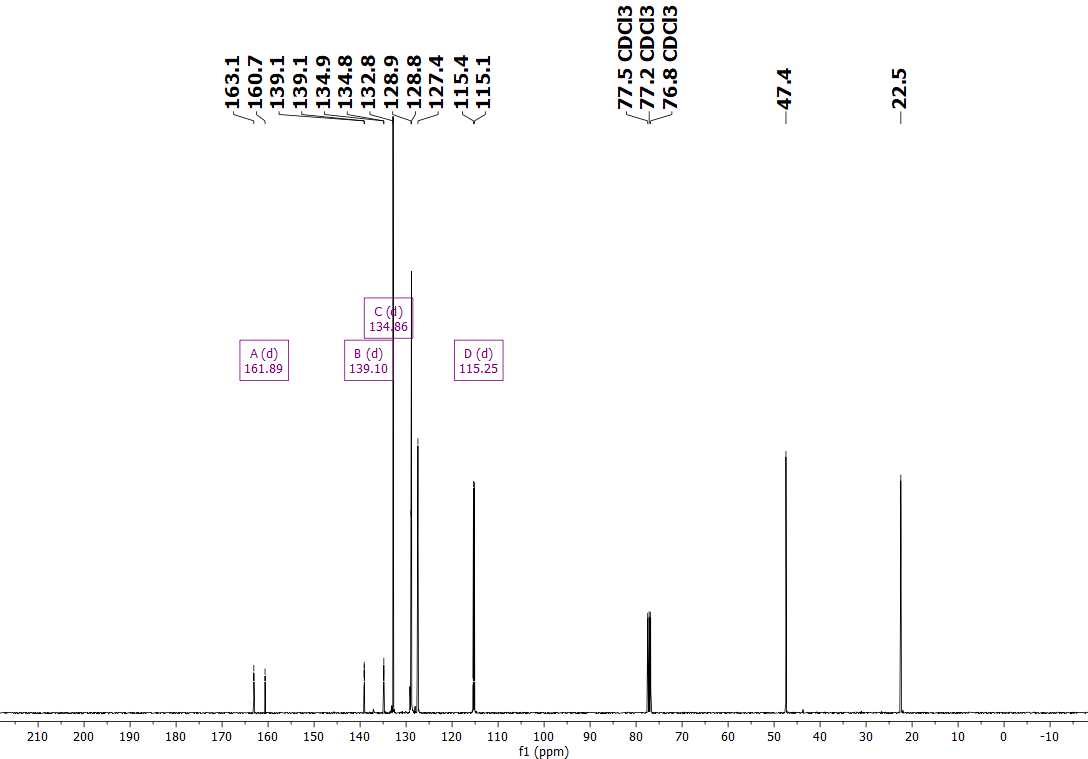


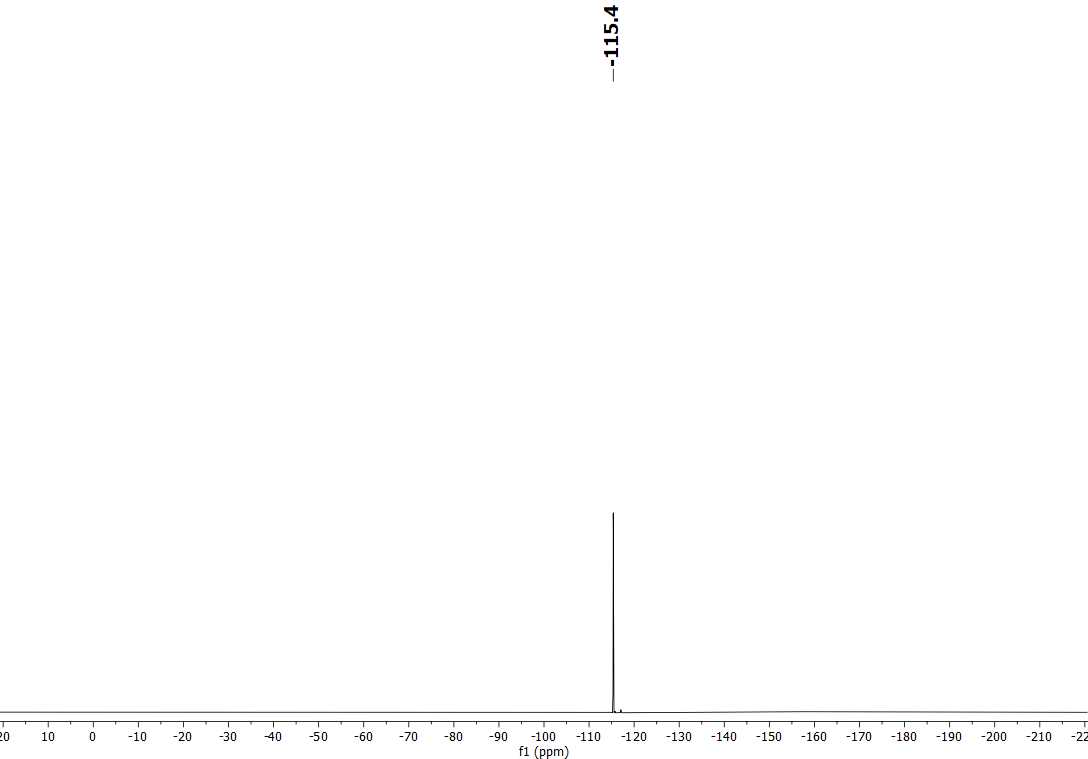


## (1-(4-Chlorophenyl)ethyl)(phenyl)sulfane (3am)


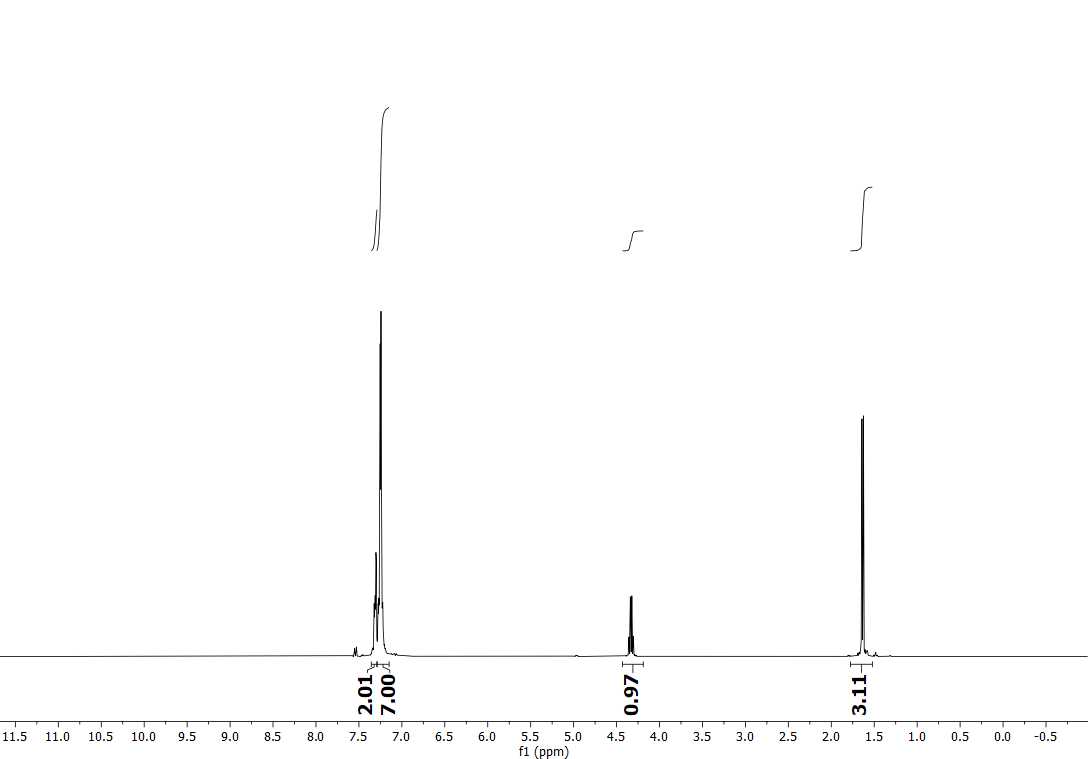


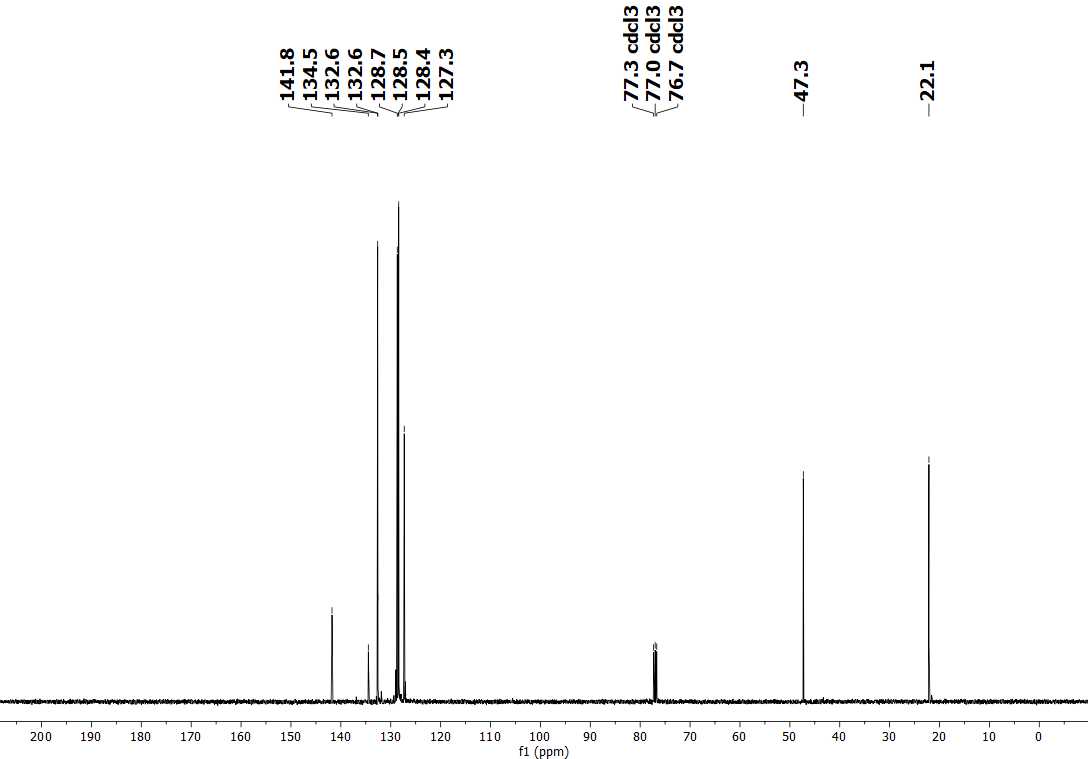


## Phenyl((1S,2R,4S)-1,7,7-trimethylbicyclo[2.2.1]heptan-2-yl)sulfane (3an)

## Benzyl(phenyl)sulfane (3ao)


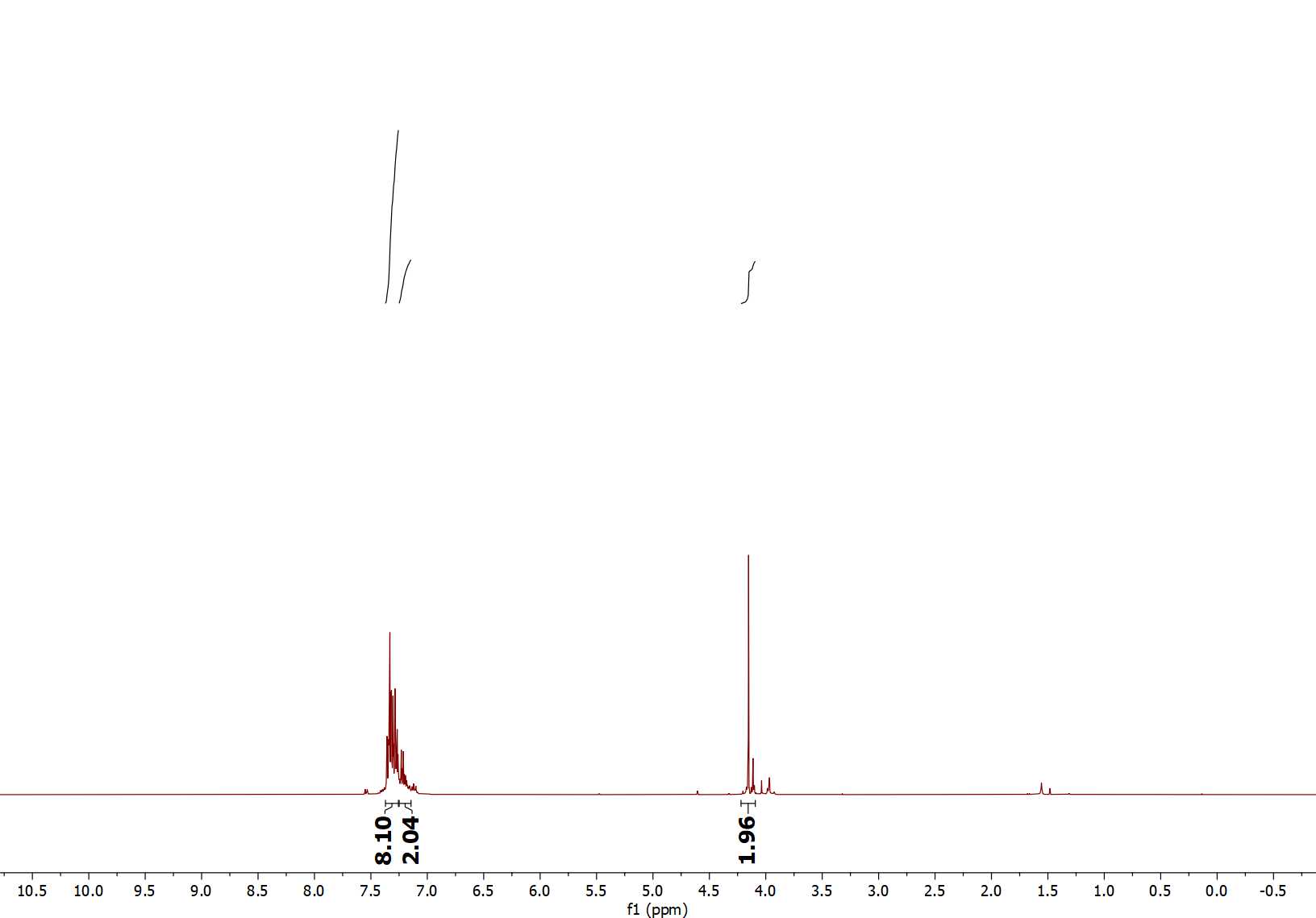


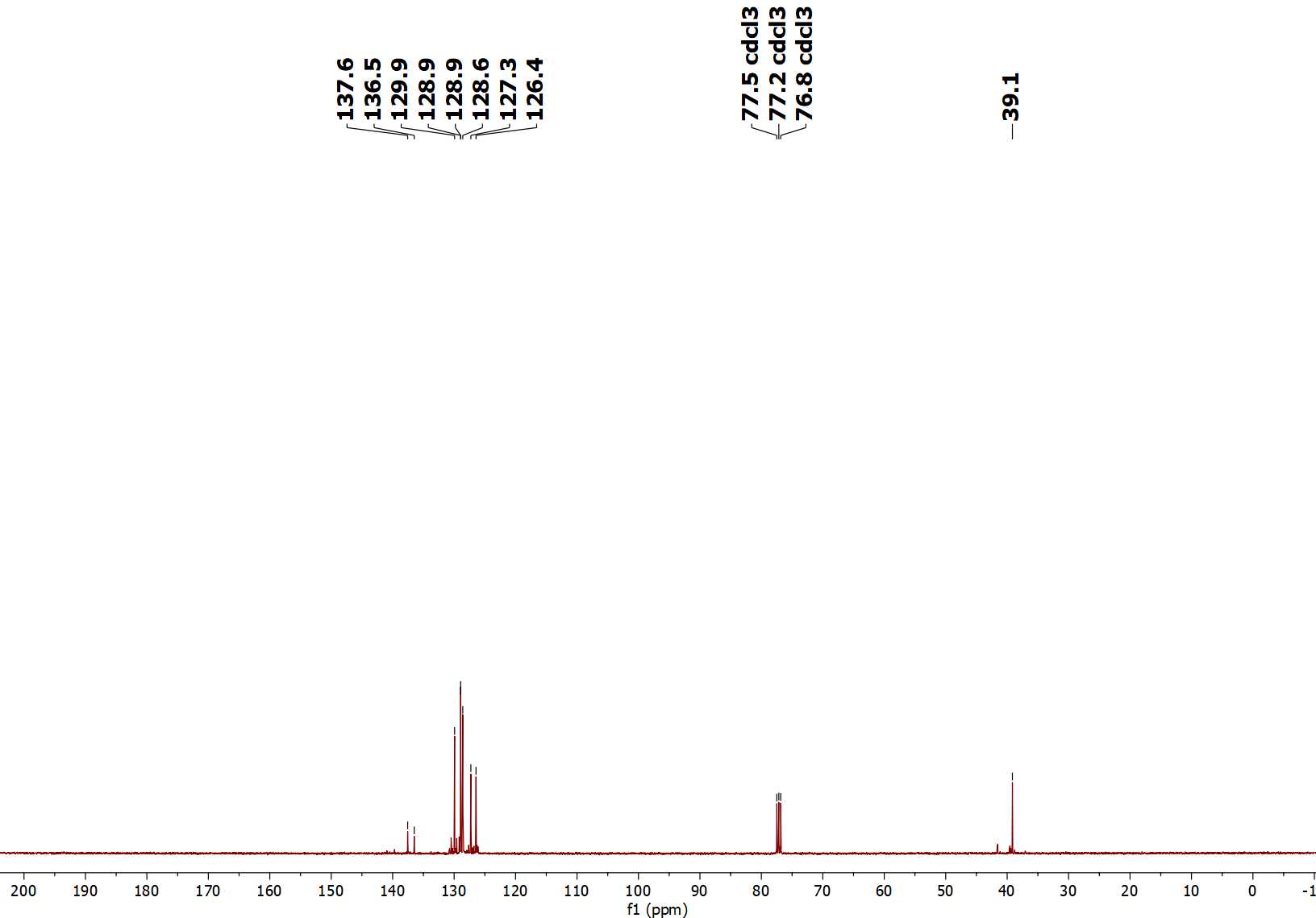


## Cinnamyl(phenyl)sulfane (3ap)


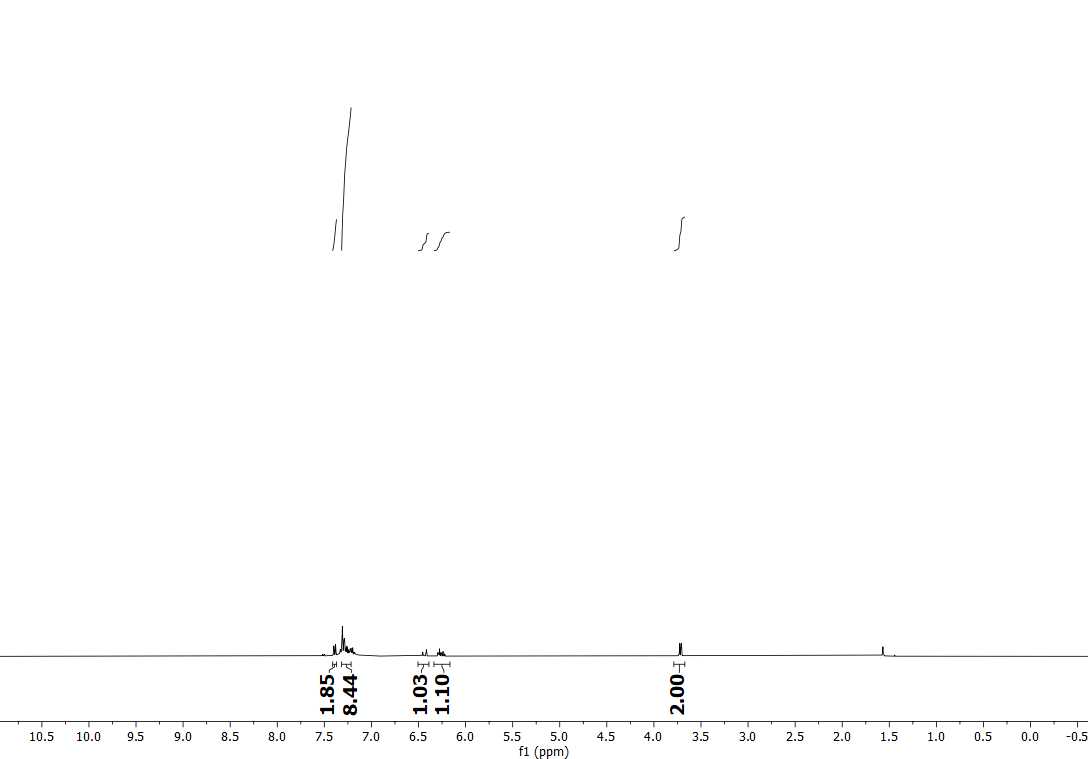


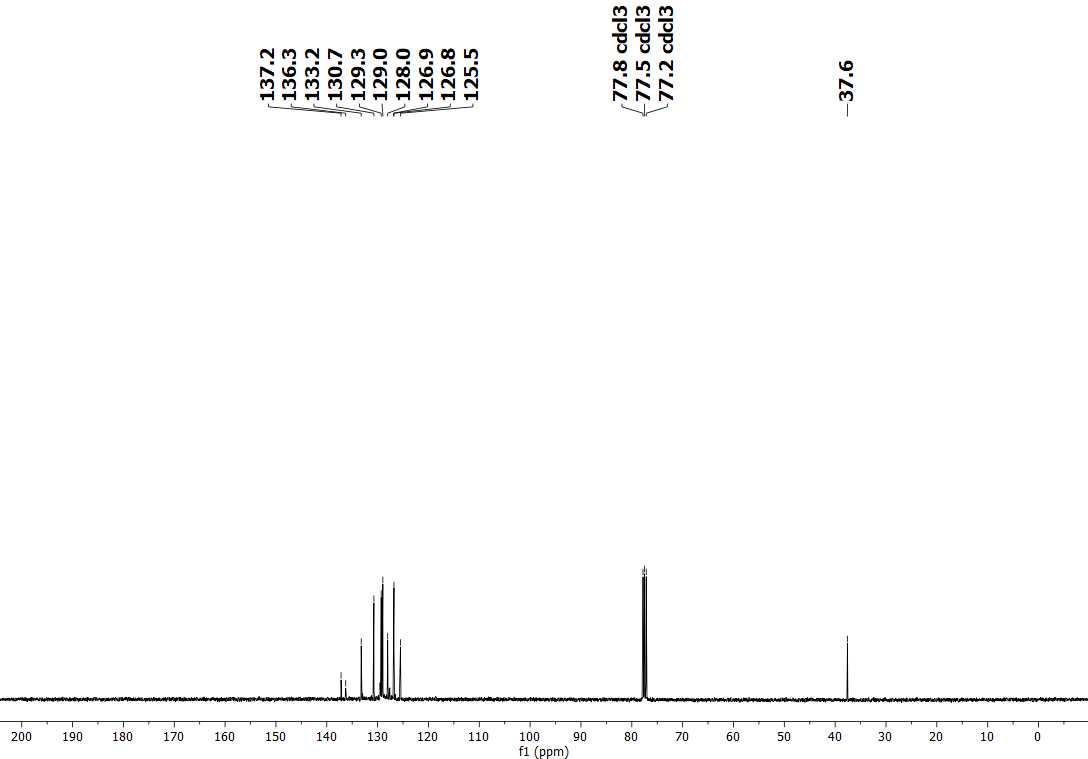


## (Cyclopropyl(phenyl)methyl)(phenyl)sulfane (3z)


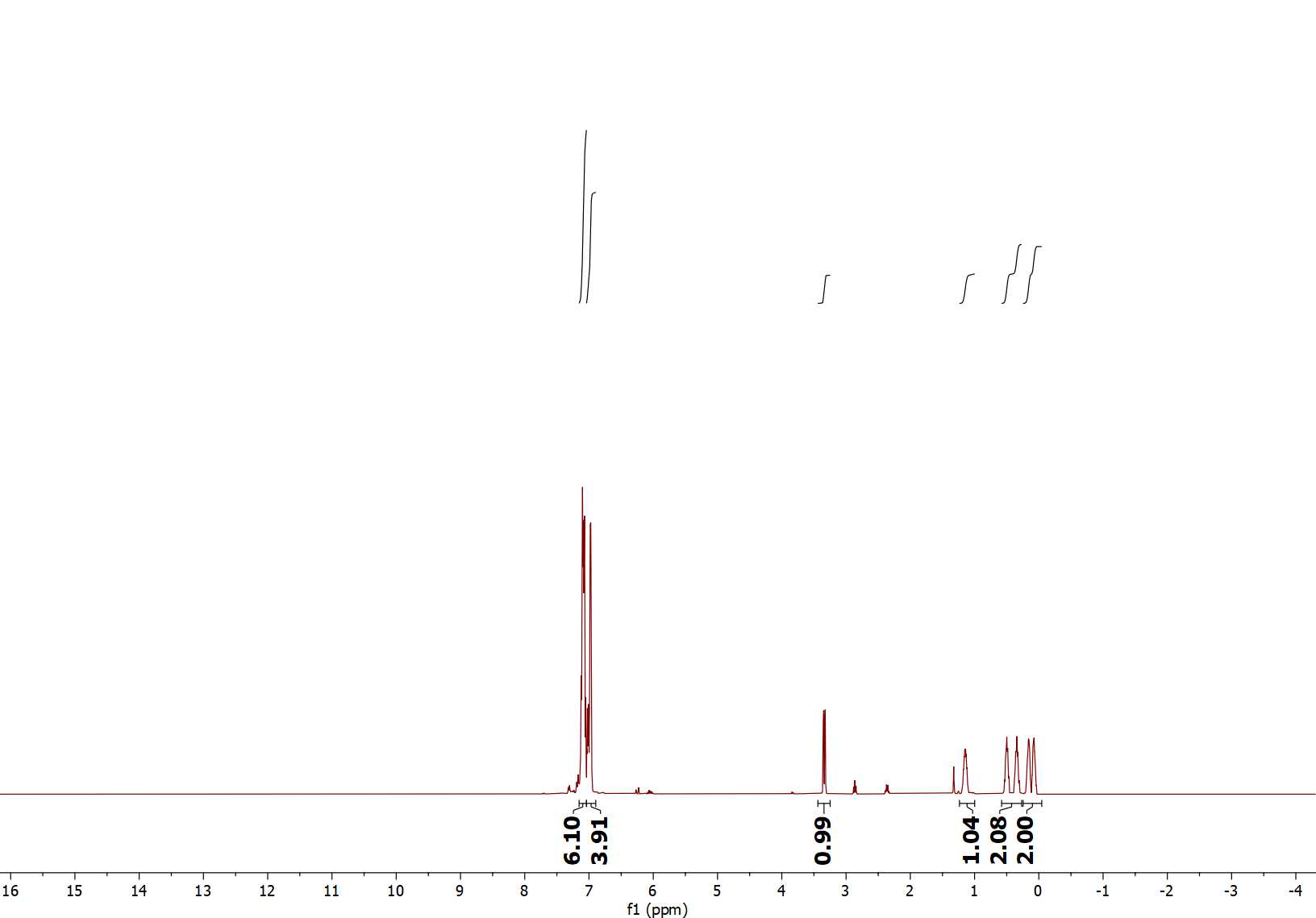


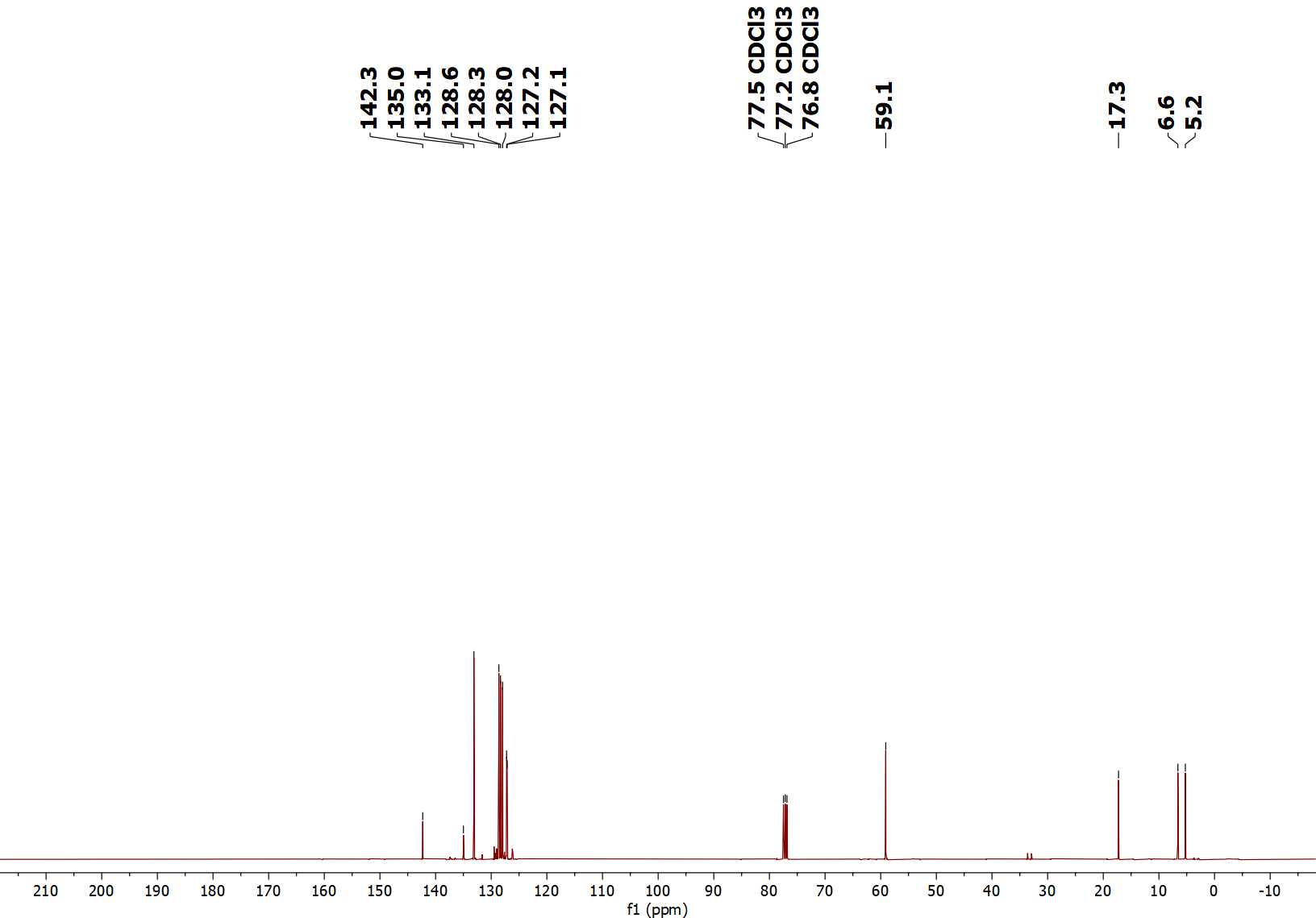

Supplement: Supplementary file 1 — Supplementary Information 1. [file 41598_2023_47938_MOESM1_ESM.docx]
